# New insights into paulomycin biosynthesis pathway in *Streptomyces albus* J1074 and generation of novel derivatives by combinatorial biosynthesis

**DOI:** 10.1186/s12934-016-0452-4

**Published:** 2016-03-21

**Authors:** Aránzazu González, Miriam Rodríguez, Alfredo F. Braña, Carmen Méndez, José A. Salas, Carlos Olano

**Affiliations:** Departamento de Biología Funcional e Instituto Universitario de Oncología del Principado de Asturias (I.U.O.P.A), Universidad de Oviedo, C/Julian Claveria s/n, 33006 Oviedo (Asturias), Spain

**Keywords:** Acyl migration, Deoxysugar, Glycosyltranferase, Paulic acid, Paulomenol, Structural analogue

## Abstract

**Background:**

*Streptomyces albus* J1074 produces glycosylated antibiotics paulomycin A, B and E that derive from chorismate and contain an isothiocyanate residue in form of paulic acid. Paulomycins biosynthesis pathway involves two glycosyltransferases, three acyltransferases, enzymes required for paulic acid biosynthesis (in particular an aminotransferase and a sulfotransferase), and enzymes involved in the biosynthesis of two deoxysugar moieties: D-allose and L-paulomycose.

**Results:**

Inactivation of genes encoding enzymes involved in deoxysugar biosynthesis, paulic acid biosynthesis, deoxysugar transfer, and acyl moieties transfer has allowed the identification of several biosynthetic intermediates and shunt products, derived from paulomycin intermediates, and to propose a refined version of the paulomycin biosynthesis pathway. Furthermore, several novel bioactive derivatives of paulomycins carrying modifications in the L-paulomycose moiety have been generated by combinatorial biosynthesis using different plasmids that direct the biosynthesis of alternative deoxyhexoses.

**Conclusions:**

The paulomycins biosynthesis pathway has been defined by inactivation of genes encoding glycosyltransferases, acyltransferases and enzymes involved in paulic acid and L-paulomycose biosynthesis. These experiments have allowed the assignment of each of these genes to specific paulomycin biosynthesis steps based on characterization of products accumulated by the corresponding mutant strains. In addition, novel derivatives of paulomycin A and B containing L-paulomycose modified moieties were generated by combinatorial biosynthesis. The production of such derivatives shows that L-paulomycosyl glycosyltransferase Plm12 possesses a certain degree of flexibility for the transfer of different deoxysugars. In addition, the pyruvate dehydrogenase system form by Plm8 and Plm9 is also flexible to catalyze the attachment of a two-carbon side chain, derived from pyruvate, into both 2,6-dideoxyhexoses and 2,3,6-trideoxyhexoses. The activity of the novel paulomycin derivatives carrying modifications in the L-paulomycose moiety is lower than the original compounds pointing to some interesting structure–activity relationships.

**Electronic supplementary material:**

The online version of this article (doi:10.1186/s12934-016-0452-4) contains supplementary material, which is available to authorized users.

## Background

**Fig. 1:**
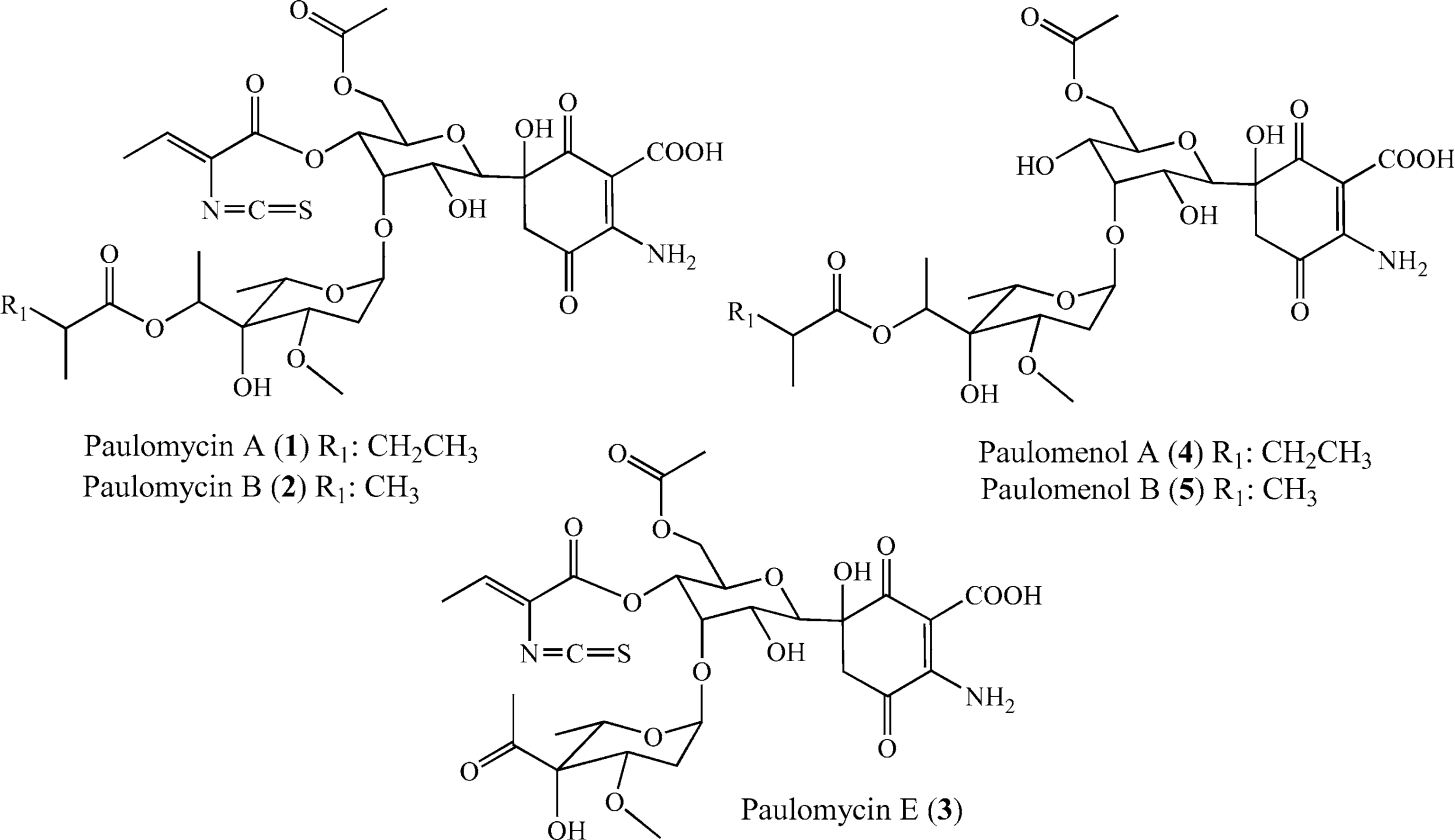
Chemical structures of paulomycins and paulomenols

Bacterial genomes sequencing and mining has become a powerful tool to identify biosynthesis gene clusters leading to produce novel secondary metabolites [1–3] and to study the biosynthesis pathway of known compounds [4–6]. Actinomycetes, in particular genus *Streptomyces*, are now one of the most highly sequenced microorganisms because of their industrial and pharmaceutical relevance as producers of enzymes and bioactive compounds [7]. The growing availability of actinomycete genome sequences has allowed intra- and inter-species comparison of genomes and secondary metabolite biosynthesis gene clusters leading to identify novel pathways and to gather increasing insights about evolution of genomes and secondary metabolism [8–11]. Intensive genome mining studies has been applied to *Streptomyces albus* strains isolated from diverse environmental niches resulting in the identification of a total of 48 unique biosynthetic gene clusters harbored by seven strains [12]. In particular, *S. albus* J1074 is the one drawing more attention since it is widely use as host for heterologous production of bioactive natural products [13, 14]. *S. albus* J1074, derivative of *S. albus* G defective in both restriction and modification enzymes of the SalI system [15], has been shown to produce, under different conditions and manipulation techniques, several carotenoids [16], hybrid polyketide-non-ribosomal peptides antimycins and 6-*epi*-alteramides, type I polyketides candicidins and non-ribosomal peptide indigoidine [14]. In addition, *S. albus* J1074 produces glycosylated compounds paulomycin A, B and E, and their derivatives paulomenol A and B (Fig. 1) [14], generated by the spontaneous loss of the paulic acid moiety.

Paulomycins A and B antibiotics containing an isothiocyanate group (paulic acid) and mainly active against Gram-positive bacteria, were initially isolated from *Streptomyces paulus* strain 273 [17, 18]. Later on, the absolute stereochemistry of these compounds was reported [19, 20] and they were found to be closely related to antibacterial compounds senfolomycins A and B, only differing in the stereochemistry of methoxy group present in their respective deoxysugar moieties [21]. *Streptomyces paulus* produces paulomycins as a family of compounds that include paulomycin A, A2, B, C, D, E and F [22], *O*-demethylpaulomycins A and B, paulomenol A and B, hydrogen sulfide adducts of paulomycin A and B [23], paldimycin A and B, and 273a_2α_ and 273a_2β_, derivatives of paulomycin A and paulomycin B containing one or two *N*-acetyl-l-cysteine groups, respectively [24, 25]. Antibiotic activity of paldimycins was assessed in vitro against 215 Gram-positive bacteria and found comparable to that of vancomycin [26]. In addition, paulomycins and paldimycins were found to be able of killing *Staphylococcus aureus* intracellular cells surviving into polymorphonuclear leukocytes [27, 28]. On the other hand, paulomenol A and B lack antibacterial activity pointing to paulic acid as determinant of the antibiotic properties of paulomycins and paldimycins [23]. Paulomycin A and B were also found to be produced by *S. albus* G [29]. Regarding the biosynthetic origin of paulomycins, addition of some precursors was found to increase production yields and they included valine, isoleucine, isobutyric acid, 2-methylburytic acid [30], l-methionine, l-threonine and α-ketoburyric acid [31, 32]. The gene cluster involved in paulomycins biosynthesis has been recently reported in *S. albus* J1074 [14], *S. paulus* NRRL8115 and *Streptomyces* sp. YN86 [33] sharing between them an overall identity of 99 %. A pathway for paulomycin biosynthesis in *S. paulus* NRRL8115, based on in silico gene analysis, has been recently proposed [33].

In this work, we characterize several steps of paulomycins biosynthesis in *S. albus* J1074 including the glycosylation and acylation steps and the biosynthesis of the paulic acid moiety. Inactivation experiments allowed to determine genes (and enzymes) involved in these steps and to identify several biosynthetic intermediates and some products accumulated by the corresponding mutants which provided insights on the biosynthesis pathway. One of the genes studied in this work, and shown to be involved in paulomycin biosynthesis, has not been previously annotated at *S. paulus* NRRL8115 and *Streptomyces* sp. YN86 paulomycin biosynthesis gene clusters. We propose a refined version of the paulomycin biosynthesis pathway based on experimental evidence. In addition, we have generated by combinatorial biosynthesis several novel bioactive derivatives of paulomycin A and B carrying modifications in the L-paulomycose moiety using different plasmids that direct the biosynthesis of different deoxyhexoses.

## Results

### Delimiting the boundaries of paulomycin biosynthesis cluster

**Fig. 2:**
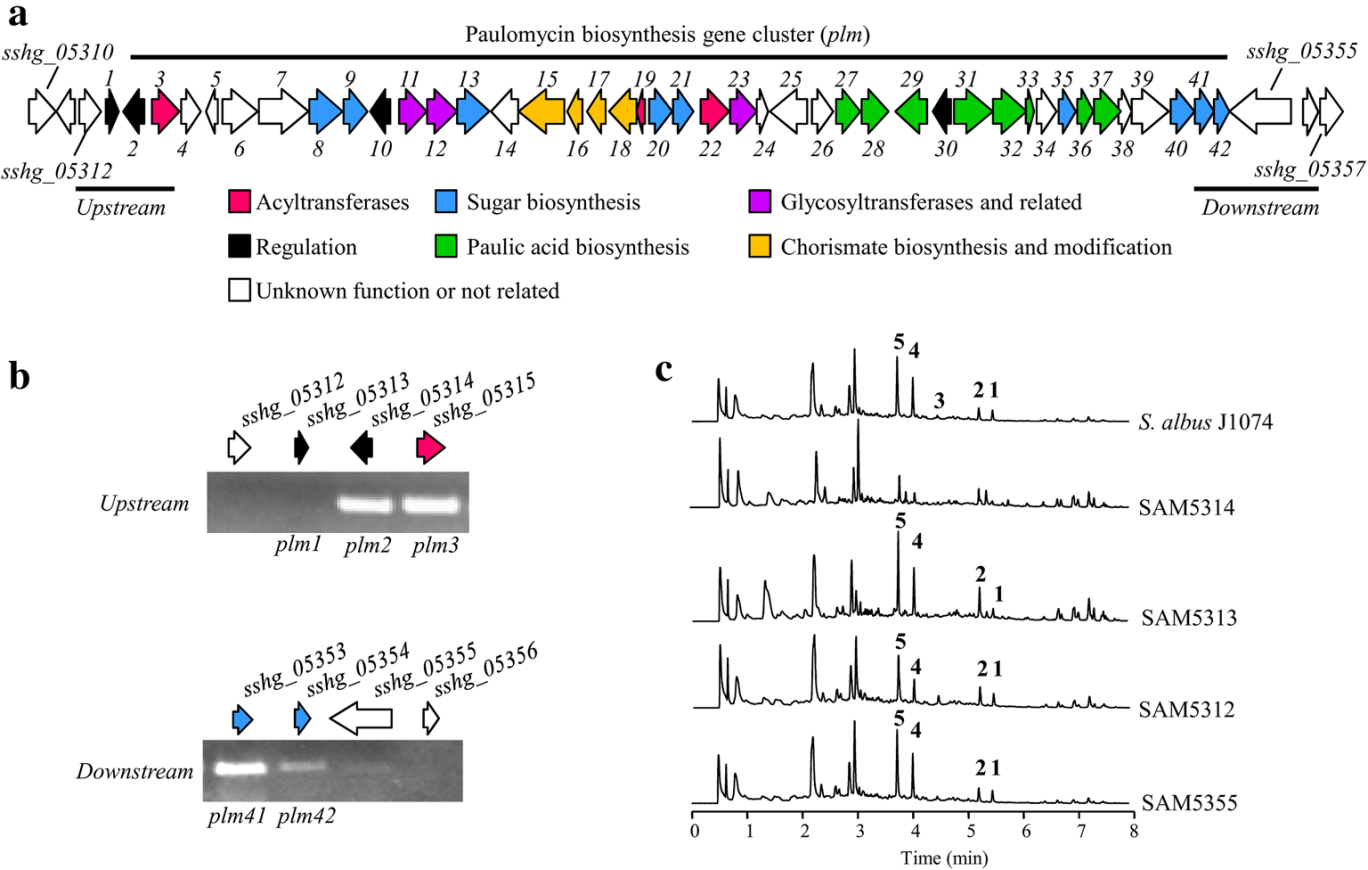
Paulomycin biosynthesis gene cluster. **a** *S. albus* J1074 genome region containing the paulomycin biosynthesis gene cluster. *Bars* represent genes at cluster boundaries analyzed by RT-PCR. **b** RT-PCR gene expression analysis of genes delimiting paulomycin biosynthesis gene cluster boundaries: *sshg_05312, sshg_05313* (*plm1*)*, sshg_05314* (*plm2*), *sshg_05315* (*plm3*), *sshg_05353* (*plm41*), *sshg_05354* (*plm42*) *sshg_05355* and *sshg_05356*. **c** UPLC chromatograms, monitored at 244 nm, of *S. albus* J1074 and SAM5314, SAM5312, SAM5313 and SAM5355 mutant strains. Labeled peaks correspond to paulomycins A (**1**), B (**2**) and E (**3**), and paulomenols A (**4**) and B (**5)**

Paulomycin biosynthesis gene cluster (Fig. 2a, Table 1) was initially delimited by *sshg_05313* and *sshg_05354* using an in silico analysis [14]. Boundaries of the cluster have now been further defined by RT-PCR gene expression analysis using *S. albus* J1074 total RNA as a template, isolated at 48 h from cultures grown in R5A that were producing paulomycins and paulomenols. Under conditions of paulomycin production, in the upstream region only *sshg_05314* and *sshg_05315* were transcribed, while in the downstream region were *sshg_05354* and *sshg_05355* (Fig. 2b). These results pointed to *sshg_05314* and *sshg_05355* as gene cluster boundaries. These results were verified by inactivation of several genes. Inactivation of *sshg_05314*, encoding a LuxR-family transcriptional regulator, led to SAM5314 mutant strain, which was still able to produce paulomycin A (**1**), B (**2**), E (**3**), paulomenol A (**4**) and B (**5**) but at considerably lower yield (20 %) than *S. albus* J1074 (Fig. 2c). This result points to *sshg_05314* as involved in regulation of paulomycin biosynthesis. Complementation of SAM5314 using pEM4HT5314 restored paulomycin production to wild type strain levels (Additional file 1: Figure S5). On the contrary, inactivation of *sshg_05312*, SAM5312 mutant strain, had no effect on paulomycin production (Fig. 2c). Otherwise, inactivation of *sshg_05313* encoding a TetR-family transcriptional regulator, led to SAM5313 mutant strain, which accumulates more paulomycin B (**2**) and paulomenol B (**5**), 2- and 1.5-fold respectively, than the wild type strain (Fig. 2c). This result points to *sshg_05313* as involved in regulation of paulomycin biosynthesis as a repressor. Considering that under conditions of paulomycin production *sshg_05313* is not transcribed it might be acting as a repressor of the pathway at early stages of *S. albus* growth when paulomycins are not produced. These results led to establish the left-hand limit of the cluster at *sshg_05313* (Fig. 2a). Inactivation of *sshg_05355* led to SAM5355 mutant strain that produced paulomycins and paulomenols at the same level than *S. albus* J1074 wild type strain (Fig. 2c). Since *sshg_05354* encodes a dTDP-4-keto-6-deoxyhexose 3,5-epimerase, which has been proposed to be involved in 2,6-deoxysugar paulomycose biosynthesis [14] and is expressed during paulomycins biosynthesis (Fig. 2b), it should be the left-hand limit of this cluster (Fig. 2a). According to these results, from here on out, we will designate the paulomycin biosynthesis genes as *plm1* (corresponding to *sshg_05313*) to *plm42* (corresponding to *sshg_05354*) (Fig. 2a, Table 1). The boundaries of the paulomycin cluster at *S. paulus* NRRL8115 have been previously delimited to *pau4* and *pau44* [33], genes that correspond to *S. albus* orthologues *plm1* and *plm42* (Table 1).

**Table 1:** Proposed functions of paulomycin biosynthesis cluster deduced proteins from *S. albus* J1074

*S. albus* J1074^a^	Cluster proteins	Proposed function	*S. paulus* NRRL8115^b^	*Streptomyces* sp. YN86^c^
SSHG_05310		RNA polymerase sigma factor	Pau1 (98/98)	PauY1 (98/99)
SSHG_05311		M23 family peptidase	Pau2 (99/100)	PauY2 (99/100)
SSHG_05312		Putative aminotransferase	Pau3 (100/100)	PauY3 (99/99)
SSHG_05313	Plm1	TetR-family transcriptional regulator	Pau4 (99/100)	PauY4 (100/100)
SSHG_05314	Plm2	LuxR-family transcriptional regulator	Pau5 (94/94)	PauY5 (96/96)
SSHG_05315	Plm3	Isovaleryltransferase	Pau6 (100/100)	PauY6 (99/99)
SSHG_05316	Plm4	Oxidoreductase	Pau7 (100/100)	PauY7 (98/99)
SSHG_05317	Plm5	Conserved hypothetical protein	Pau8 (100/100)	PauY8 (100/100)
SSHG_05318	Plm6	EmrB/QacA subfamily transporter	Pau9 (100/100)	PauY9 (99/99)
SSHG_05319	Plm7	Elongation factor G 1	Pau10 (99/99)	PauY10 (99/99)
SSHG_05320	Plm8	Dehydrogenase E1 alpha subunit	Pau11 (100/100)	PauY11 (99/99)
SSHG_05321	Plm9	Dehydrogenase E1 beta subunit	Pau12 (99/99)	PauY12 (99/99)
SSHG_05322	Plm10	SARP-family transcriptional regulator	Pau13 (99/100)	PauY13 (100/100)
SSHG_05323	Plm11	Cytochrome P450-like	Pau14 (99/99)	PauY14 (98/99)
SSHG_05324	Plm12	Glycosyltransferase	Pau15 (99/99)	PauY15 (99/99)
SSHG_05325	Plm13	NDP-hexose 2,3-dehydratase	Pau16 (99/99)	PauY16 (99/99)
SSHG_05326	Plm14	3-hydroxybenzoate 6-hydroxylase	Pau17 (99/100)	PauY17 (99/99)
SSHG_05327	Plm15	Anthranilate synthase	Pau18 (99/99)	PauY18 (99/99)
SSHG_05328	Plm16	Isochorismatase	Pau19 (99/99)	PauY19 (99/99)
SSHG_05329	Plm17	2,3-dihydro-2,3-dihydroxybenzoate dehydrogenase	Pau20 (99/99)	PauY20 (99/99)
SSHG_05330	Plm18	3-deoxy-D-arabino-heptulosonate-7-phosphate synthase	Pau21 (99/99)	PauY21 (99/99)
SSHG_05331	Plm19	Predicted GCN5-related N-acetyltransferase	–	–
SSHG_05332	Plm20	dTDP-glucose 4,6-dehydratase	Pau22 (99/99)	PauY22 (99/99)
SSHG_05333	Plm21	D-glucose-1-phosphate synthase	Pau23 (99/99)	PauY23 (99/99)
SSHG_05334	Plm22	Isovaleryltransferase	Pau24 (99/99)	PauY24 (99/99)
SSHG_05335	Plm23	C-glycosyltransferase	Pau25 (100/100)	PauY25 (99/99)
SSHG_05336	Plm24	Glyoxalase	Pau26 (99/100)	PauY26 (99/100)
SSHG_05337	Plm25	FAD-binding monooxygenase	Pau27 (99/100)	PauY27 (99/99)
SSHG_05338	Plm26	Hypothetical protein, Reductase	Pau28 (99/99)	PauY28 (99/99)
SSHG_05339	Plm27	3-oxoacyl-ACP synthase III	Pau29 (99/99)	PauY29 (100/100)
SSHG_05340	Plm28	Putative sulfotransferase	Pau30 (100/100)	PauY30 (99/98)
SSHG_05341	Plm29	Aminotransferase	Pau31 (99/100)	PauY31 (98/98)
SSHG_05342	Plm30	LuxR-family transcriptional regulator	Pau32 (100/100)	PauY32 (100/100)
SSHG_05343	Plm31	Oxidoreductase	Pau33 (99/99)	PauY33 (98/98)
SSHG_05344	Plm32	Acyl-CoA synthase	Pau34 (99/99)	PauY34 (100/100)
SSHG_05345	Plm33	Acyl-carrier protein	Pau35 (100/100)	PauY35 (100/100)
SSHG_05346	Plm34	Dihydrodipicolinate reductase	Pau36 (100/100)	PauY36 (100/100)
SSHG_05347	Plm35	Ribulose-5-phosphate 4-epimerase	Pau37 (99/99)	PauY37 (100/100)
SSHG_05348	Plm36	4′-phosphopantetheinyl transferase	Pau38 (99/99)	PauY38 (100/100)
SSHG_05349	Plm37	Acyl-CoA dehydrogenase	Pau39 (99/100)	PauY39 (99/99)
SSHG_05350	Plm38	Hypothetical protein	Pau40 (98/100)	PauY40 (97/99)
SSHG_05351	Plm39	Pyranose oxidase	Pau41 (100/100)	PauY41 (100/100)
SSHG_05352	Plm40	dTDP-4-keto-6-deoxy-L-hexose 2,3-reductase	Pau42 (99/99)	PauY42 (99/99)
SSHG_05353	Plm41	dTDP-6-deoxy-L-hexose 3-*O*- methyltransferase	Pau43 (100/100)	PauY43 (100/100)
SSHG_05354	Plm42	dTDP-4-keto-6-deoxyhexose 3,5-epimerase	Pau44 (100/100)	PauY44 (100/100)
SSHG_05355		Malate synthase	Pau45 (99/100)	PauY45 (99/100)
SSHG_05356		Oxidoreductase	Pau46 (97/98)	PauY46 (98/98)
SSHG_05357		Oxidoreductase	Pau47 (100/100)	PauY47 (99/99)

The correlation of *S. albus* J1074 proteins with orthologues from *S.* *paulus* NRRL8115 and *Streptomyces* sp. YN86 paulomycin biosynthesis gene clusters is shown

^a^ Proteins corresponding to nucleotide accession number NZ_DS999645.1

^b^ Proteins corresponding to nucleotide accession number KJ721164.1. % identity/similarity (in parenthesis)

^c^ Proteins corresponding to nucleotide accession number KJ721165.1. % identity/similarity (in parenthesis)

### Regulation of paulomycin biosynthesis gene cluster

**Fig. 3:**
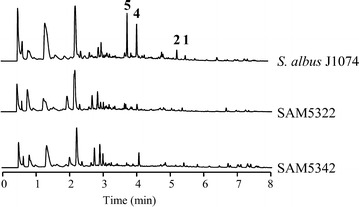
Characterization of regulatory genes. UPLC chromatograms, monitored at 244 nm, of *S. albus* J1074 and SAM5322 and SAM5342 mutant strains. All extracts were generated by culturing mutant strains in R5A liquid medium during 96 h. Labeled peaks correspond to paulomycin A (**1**), paulomycin B (**2**), paulomenol A (**4**) and paulomenol B (**5**)

In addition to *plm1* and *plm2*, two more genes, *plm10* and *plm30*, might be involved in regulation of paulomycin biosynthesis. Plm10 shows similarity to transcriptional regulators of SARP-family and Plm30 might correspond to the LuxR-family of transcriptional regulators. In both cases, these putative regulatory proteins present orthologues (Pau13/PauY13 and Pau32/PauY32, respectively) into paulomycin biosynthesis clusters in *S. paulus* (KJ721164.1) and *Streptomyces* sp.YN86 (KJ721165.1). Independent inactivation of *plm10* and *plm30* led to SAM5322 and SAM5342 mutant strains, respectively. Both mutants were unable to produce paulomycins A and B or their derivatives paulomenols A and B (Fig. 3), confirming the role of *plm10* and *plm30* as positive regulators in paulomycin biosynthesis. Both mutant strains were growing at the same rate as the wild type strains measured by dry weight. Complementation of SAM5322 and SAM5342 using pEM4HTSARP and pEM4HTLuxR, respectively, partially restored (20 % in each case) paulomycins and paulomenols production (Additional file 1: Figure S5).

### Glycosyltransferases involved in paulomycin biosynthesis

Two glycosyltransferases must be involved in the incorporation of D-allose and L-paulomycose during paulomycin biosynthesis. Plm12 and Plm23 show similarities to *O*- and *C*-glycosyltransferases from different streptomycetes including Pau15/PauY15 and Pau25/PauY25, respectively, of paulomycin biosynthesis clusters in *S. paulus* (KJ721164.1) and *Streptomyces* sp.YN86 (KJ721165.1). In addition, Plm11 shows similarity to cytochrome P450-like enzymes that lack the conserved Cys necessary to bind heme prosthetic group. These enzymes have been shown to participate in glycosylation by activating their counterpart glycosyltransferase [34].

**Fig. 4:**
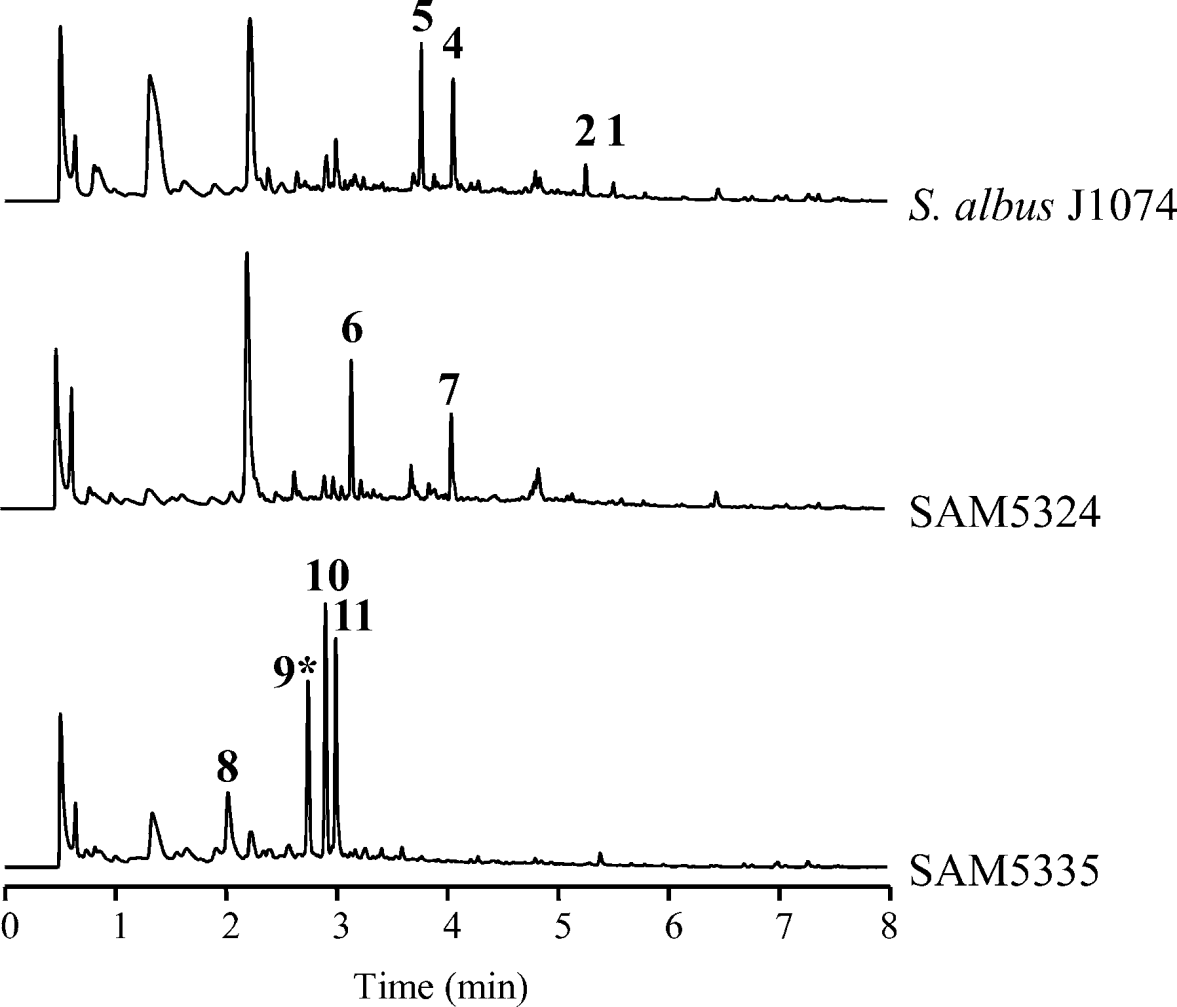
Characterization of genes encoding glycosyltransferases. UPLC chromatograms, monitored at 244 nm, of *S. albus* J1074 mutant strains altered in paulomycins biosynthesis by inactivation of glycosyltransferase coding genes *plm12* (SAM5324) and *plm23* (SAM5335). All extracts were generated by culturing mutant strains in R5A liquid medium during 96 h. Labeled peaks correspond to paulomycin A (**1**), paulomycin B (**2**), paulomenol A (**4**), paulomenol B (**5**), 6-hydroxyl-paulinone (**6**), (*2E*)-17-(4′-aminophenyl)-3,11,15-trihydroxy-10,12,14-trimethyl-17-oxo-heptadeca-4,6,8-trienoic acid (**7**), 2-aminobenzoic acid (anthranilic acid) (**8**), compound not characterized (9*), *N*-acetyl-*orto*-aminobenzoic acid (**10**), deoxydehydrochorismic acid (**11**)

**Fig. 5:**
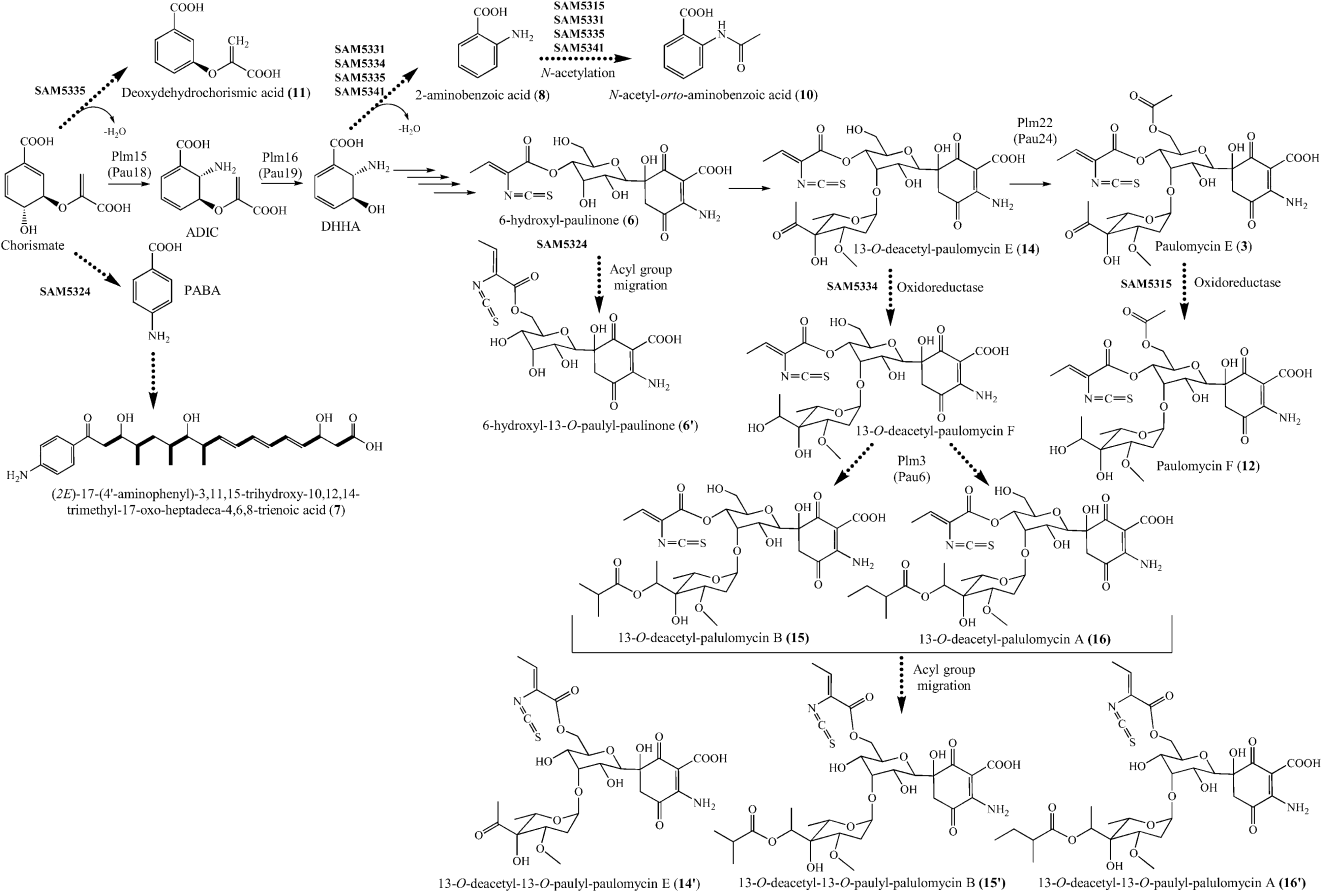
Proposed origin of compounds accumulated by *S. albus* J1074 mutant strains altered in paulomycins biosynthesis. Paulomycin biosynthesis orthologues from *S. paulus* NRRL8115 [33] are depicted in parenthesis. Compounds are pointed by *dotted arrows* starting from the compound they might derive. ADIC, 2-amino-2-deoxyisochorismate; DHHA, 2,3-dihydro-3-hydroxyanthranilate; PABA, *p*-aminobenzoic acid; PKS, polyketide synthase

Inactivation of *plm12* led to SAM5324 mutant strain (Fig. 4), unable to produce paulomycins A or B but producing instead a compound (**6**) with UPLC retention time of 3.17 min, showing the characteristic paulomycin absorption spectrum with maxima at 236, 275 and 323 nm, and a mass of *m/z* 473 [*M* + H]^+^. The similarity of compound **6** and paulomycins absorption spectra pointed to the presence of paulic acid in **6** structure. Furthermore, compound **6** mass is in concordance with paulomycin intermediate 6-hydroxyl-paulinone (Fig. 5). In consonance with this, compound **6** was converted into paulomycin A (**1**) and B (**2**) by co-culturing *S. albus* B29 strain, a mutant altered at early steps in paulomycin biosynthesis [14], and SAM5324. The same result was obtained by co-culturing SAM5335 (see below) and SAM5324 mutant strains (Additional file 2: Figure S6). 6-hydroxyl-paulinone (**6**) suffers, during its purification and analysis, an acyl migration of the paulic acid moiety. Thus, characterization of this compound by NMR (Additional file 3: Figures S8–S14, Table S2) identified it as 6-hydroxyl-13-*O*-paulyl-paulinone (**6′**) (Fig. 5), which in fact contains a paulic acid moiety that has migrated from its normal position at C11 hydroxyl group (D-allose C4) to C13 hydroxyl moiety (D-allose C6). 6-hydroxyl-13-*O*-paulyl-paulinone (**6′**) is not a real paulomycin biosynthetic intermediate since no conversion to paulomycin A and B was observed by feeding **6′** to *S. albus* B29 mutant strain. SAM5324 produced, in addition, a second compound (**7**) with UPLC retention time of 4.07 min, absorption spectrum with maxima at 269 and 316 nm, and a mass of *m/z* 460 [*M* + H]^+^. This compound was characterized by NMR (Additional file 3: Figures S15–S19, Table S3) as (*2E*)-17-(4′-aminophenyl)-3,11,15-trihydroxy-10,12,14-trimethyl-17-oxo-heptadeca-4,6,8-trienoic acid (Fig. 5), showing no structural relationship with paulomycins and corresponding to an incomplete intermediate of candicidin. This compound might result from the accumulation of chorismate and its conversion into *p*-aminobenzoic acid (PABA), direct precursor of candicidins (Fig. 5).

SAM5335 mutant strain, which was generated by deletion of *plm23*, produces four compounds (Fig. 4) with UPLC retention times of 2.0, 2.7, 2.9 and 3.0 min, respectively. Compound **8** with UPLC retention time of 2.0 min, showed maxima of absorbance at 223 and 329 nm, and a mass of *m/z* 138 [*M* + H]^+^. It was identified as 2-aminobenzoic acid (anthranilic acid) (Fig. 5) by NMR (Additional file 3: Figures S20–S23, Table S4). Compounds **9** and **10** with UPLC retention times of 2.7 and 2.9 min, respectively, shared a similar absorption spectrum with maxima at 223, 251 and 302 nm. Compound **10**, revealed by mass analysis an ion of *m/z* 180 [*M* + H]^+^, and was identified by NMR (Additional file 3: Figures S24–S26) as *N*-acetyl-*orto*-benzoic acid (Fig. 5). Compound **11** with UPLC retention times of 3.0 min, a mass of *m/z* 209 [*M* + H]^+^ and maxima of absorption at 207 and 288 nm, was characterized by NMR (Additional file 3: Figures S27–S29) as deoxydehydrochorismic acid (Fig. 5). These compounds, 2-aminobenzoic acid (**8**), *N*-acetyl-*orto*-benzoic acid (**10**) and deoxydehydrochorismic acid (**11**), are not paulomycin intermediates since they failed restoring paulomycins biosynthesis in bioconversion experiments using *S. albus* B29 mutant strain. Complementation of SAM5324 and SAM5335 using pEM4HT5324 and pEM4HT5335 respectively, partially restored (20 % and 10 %, respectively) paulomycins and paulomenols production (Additional file 1: Figure S5).

### Acyltransferases involved in paulomycin biosynthesis

Three acyltransferases might be involved in the incorporation of paulyl-CoA, acyl-CoA and 2-methylbutyryl-CoA or isobutyryl-CoA during paulomycin A and B biosynthesis, respectively. Plm3 and Plm22 show similarity to *O*-acyltransferases MegY (AAG13909.1) and TcaM (ACB37733.1) involved in megalomicin and tetrocarcin A biosynthesis, respectively. In addition, they show similarity to isovaleryltransferases from different origins including Pau6/PauY6 and Pau24/PauY24, respectively, in *S. paulus* (KJ721164.1) and *Streptomyces* sp.YN86 (KJ721165.1) paulomycin biosynthesis clusters. On the other hand, Plm19 show similarity to GCN5-related *N*-acetyltransferase bthur0008_41520 (ZP_04104064.1). However, Plm19 has no orthologues annotated in paulomycin biosynthesis clusters in *S. paulus* (KJ721164.1) and *Streptomyces* sp.YN86 (KJ721165.1) [33], but the corresponding gene is present in both sequences between *pau21* and *pau22* in *S. paulus* NRRL8115 and *pauY21* and *pauY22* in *Streptomyces* sp.YN86.

**Fig. 6:**
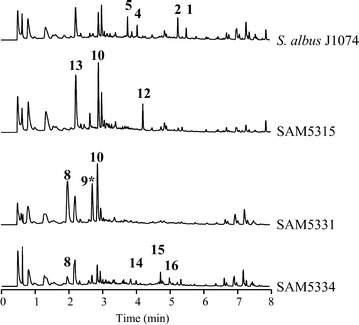
Characterization of genes encoding acyltransferases. UPLC chromatograms, monitored at 244 nm, of *S. albus* J1074 mutant strains altered in paulomycins biosynthesis by inactivation of acyltransferase coding genes *plm3* (SAM5315), *plm19* (SAM5331) and *plm22* (SAM5334). Extracts were generated by culturing mutant strains in R5A liquid medium during 96 h. Labeled peaks correspond to paulomycin A (**1**), paulomycin B (**2**), paulomenol A (**4**), paulomenol B (**5**), 2-aminobenzoic acid (anthranilic acid) (**8**), compound not characterized (9*), *N*-acetyl-*orto*-aminobenzoic acid (**10**), deoxydehydrochorismic acid (**11**), paulomycin F (**12**), antimycic acid (**13**), 13-*O*-deacetyl-paulomycin E (**14**), 13-*O*-deacetyl-paulomycin B (**15**) and 13-*O*-deacetyl-paulomycin A (**16**)

Inactivation of *plm3* led to SAM5315 mutant strain that produced (Fig. 6) compound **10** and novel compound **12** with UPLC retention time of 4.2 min, which shows a paulomycin absorption spectrum with maxima at 238, 275 and 318 nm, and a mass of *m/z* 703 [*M* + H]^+^. Characterization of compound **12** by NMR (Additional file 3: Figures S30–S37, Table S5) identified it as paulomycin F (Fig. 5), a paulomycin E derivative previously described [22] that contain a reduced keto group at paulomycose moiety. Paulomycin F (**12**) is a paulomycin intermediate since it was converted into paulomycin A and B by feeding it to *S. albus* SAM3335 mutant strain (data not shown). In addition, SAM5315 also accumulated, in higher amounts than the wild type strain, a compound with UPLC retention time of 2.2 min previously observed in *S. albus* J1074 [14] that corresponds to antimycins biosynthetic intermediate antimycic acid (**13**) (Fig. 6).

**Fig. 7:**
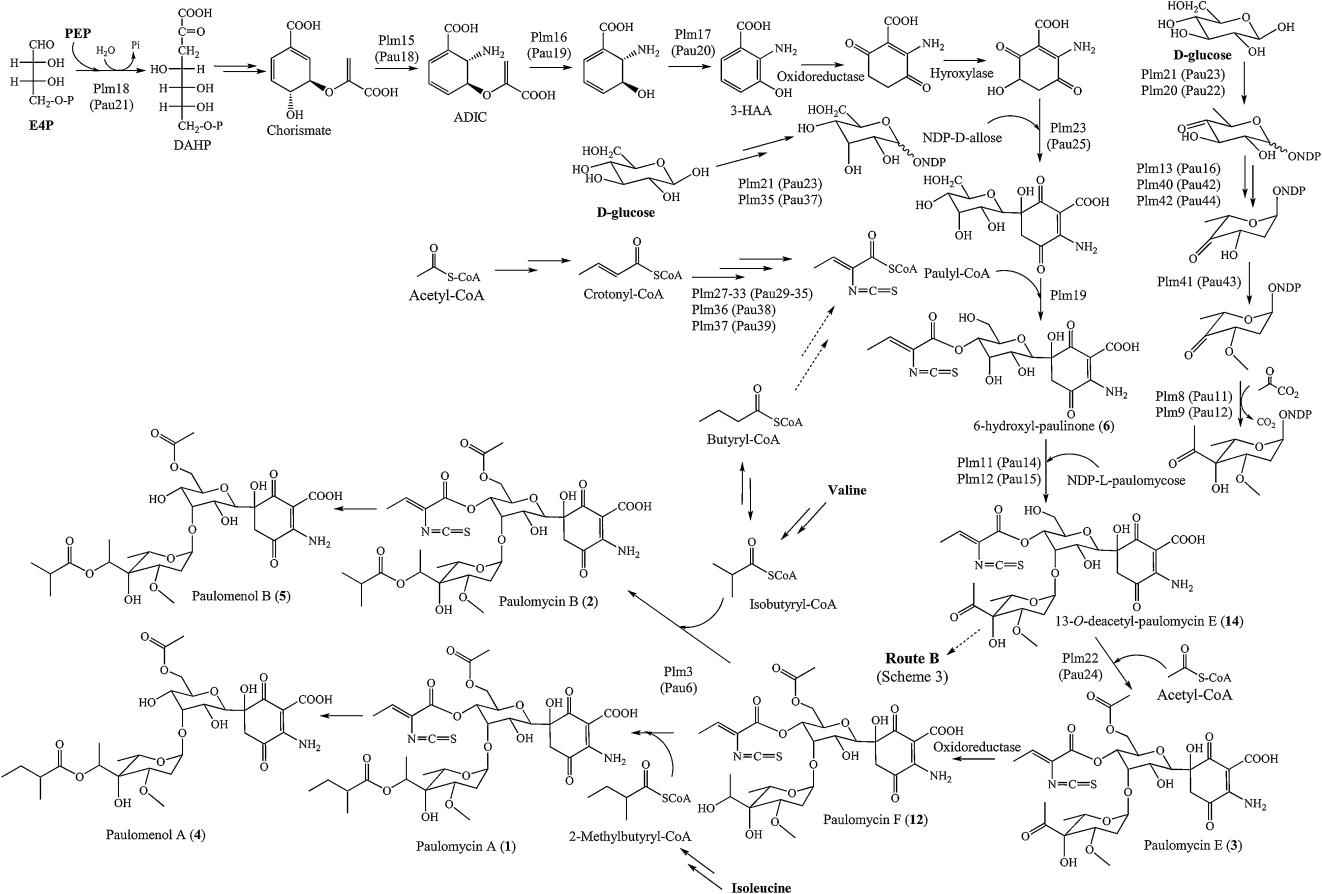
Proposed paulomycin A and B biosynthesis pathway (Route A). Orthologues from *S. paulus* NRRL8115 [33] are depicted in parenthesis. Last step in paulomycose by incorporation of a pyruvate unit has been previously characterized biochemically in C4-branched 3,6-dideoxyhexose yersiniose A biosynthesis [38]. ADIC, 2-amino-2-deoxyisochorismate; DAHP, 3-deoxy-D-arabinose-heptulosonic 7-phosphate; DHHA, 2,3-dihydro-3-hydroxyanthranilate; E4P, erythrose-4-phosphate; 3-HAA, 3-hydroxyanthranilic acid; PEP, phosphoenolpyruvate

**Fig. 8:**
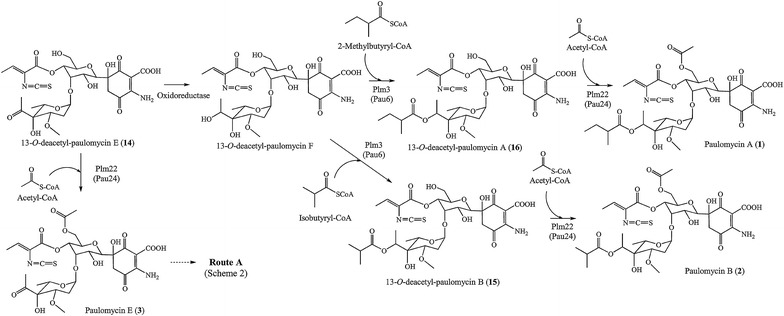
Proposed paulomycin A and B biosynthesis pathway from 13-*O*-deacetyl-paulomycin E (Route B)

SAM5331 mutant strain, generated by deleting *plm19*, was also unable to produce paulomycins (Fig. 6) and instead produced *N*-acetyl-*orto*-benzoic acid (**10**) and 2-aminobenzoic acid (**8**) (Fig. 5). In addition, SAM5334 mutant strain, generated by deleting *plm22*, was unable to produce paulomycins (Fig. 6) but it produced instead low amounts of 2-aminobenzoic acid (**8**) and three novel compounds, **14**, **15** and **16** with UPLC retention times of 3.9, 4.7 and 5.0 min, paulomycin absorption spectrum with maxima at 234, 275 and 323 nm, and masses of *m/z* 659, 731 and 745 [*M* + H]^+^, respectively. These masses are in concordance with paulomycin E, B and A derivatives 13-*O*-deacetyl-paulomycin E (**14**), 13-*O*-deacetyl-paulomycin B (**15**) and 13-*O*-deacetyl-paulomycin A (**16**) (Fig. 5). Co-culture of SAM5335 and SAM5334 showed the conversion of 13-*O*-deacetyl-paulomycin E (**14**), 13-*O*-deacetyl-paulomycin B (**15**) and 13-*O*-deacetyl-paulomycin A (**16**) into paulomycin A (**1**) and B (**2**) (Additional file 2: Figure S7), result that implies there are two alternative pathways for paulomycin A and B biosynthesis starting from 13-*O*-deacetyl-paulomycin E (**14**) (Figs. 7 and 8). As in the case of 6-hydroxyl-paulinone (**6**) described above, 13-*O*-deacetyl-paulomycin E (**14**), 13-*O*-deacetyl-paulomycin B (**15**) and 13-*O*-deacetyl-paulomycin A (**16**) suffered, during their purification and analysis, an acyl migration of the paulic acid moiety. Thus, characterization of these compounds by NMR (Additional file 3: Figures S38–S61, Tables S1–S8) identified them as 13-*O*-deacetyl-13-*O*-paulyl-paulomycin E (**14′**), 13-*O*-deacetyl-13-*O*-paulyl-paulomycin B (**15′**) and 13-*O*-deacetyl-13-*O*-paulyl-paulomycin A (**16′**) (Scheme 2). Compounds **14′**, **15′** and **16′** are not real paulomycin biosynthetic intermediates since no conversion to paulomycin A and B was observed by feeding to *S. albus* SAM5335 mutant strain. Complementation of SAM5315, SAM5331 and SAM5334 using pEM4HT5315, pEM4HT5331 and pEM4HT5334, respectively, partially restored (70, 50 and 50 %, respectively) paulomycins and paulomenols biosynthesis (Additional file 1: Figure S5).

### Enzymes involved in paulic acid biosynthesis

Several activities might be involved in the biosynthesis of the paulic acid moiety. Paulic acid contains an isothiocyanate residue (−N = C = S) that determines the paulomycin characteristic absorbance peak at 275 nm and confers antibiotic activity to paulomycins [23]. Since the isothiocyanate moiety presents nitrogen and sulfur atoms there must be in the cluster, genes encoding an aminotransferase and a sulfotransferase involved in the introduction of these components. In the biosynthesis cluster, *plm28* encodes a protein containing an UBA/THIF-type NAD/FAD binding fold (IPR000594) that might act as a sulfotransferase based on the similarity to MoeZ-like enzymes, which transfer sulfur during molybdopterin biosynthesis [35]. On the other hand, *plm29* encodes a putative aminotransferase. For both enzymes, Plm28 and Plm29, there are orthologues (Pau30/PauY30 and Pau31/PauY31) annotated in paulomycin biosynthesis clusters of *S. paulus* (KJ721164.1) and *Streptomyces* sp.YN86 (KJ721165.1), respectively.

**Fig. 9:**
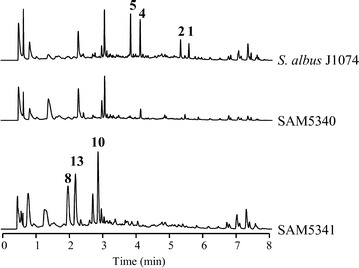
Characterization of genes involved in paulic acid biosynthesis. UPLC chromatograms, monitored at 244 nm, of *S. albus* J1074 mutant strains altered in paulomycins biosynthesis by inactivation of *plm28* (SAM5340) and *plm29* (SAM5341) involve in paulic acid biosynthesis. Extracts were generated by culturing mutant strains in R5A liquid medium during 96 h. Labeled peaks correspond to paulomycin A (**1**), paulomycin B (**2**), paulomycin E (**3**), paulomenol A (**4**), paulomenol B (**5**), 2-aminobenzoic acid (anthranilic acid) (**8**), *N*-acetyl-*orto*-aminobenzoic acid (**10**), deoxydehydrochorismic acid (**11**)

Inactivation of *plm28* led to SAM5340 mutant strain, unable to produce paulomycins or any other related compound (Fig. 9). Similarly, deletion of *plm29* led to SAM5341 mutant, unable to produce paulomycins but that accumulated compounds **8**, **10** and antimycic acid (**13**) (Fig. 9). Complementation of SAM5340 and SAM5341 using pEM4HT5340 and pEM4HT5341, respectively, restored paulomycins and paulomenols production at different levels: to wild type yield in the case of mutant SAM5340 and to 40 % of wild type levels in the case of mutant SAM5341 (Additional file 1: Figure S5).

### Biosynthesis of L-paulomycose and generation of novel paulomycin derivatives

The paulomycin biosynthesis gene cluster contains all genes necessary for the biosynthesis of L-paulomycose. An intermediate in the biosynthesis of L-olivose, NDP-4-keto-L-olivose [36], might be generated by the activity of d-glucose-1-phosphate synthase Plm21, dTDP-glucose 4,6-dehydratase Plm20, NDP-hexose 2,3-dehydratase Plm13, dTDP-4-keto-6-deoxy-L-hexose 2,3-reductase Plm40 and dTDP-4-keto-6-deoxyhexose 3,5-epimerase Plm42. The intermediate NDP-4-keto-L-olivose might then suffer an *O*-methylation, most probably by dTDP-6-deoxy-L-hexose 3-*O*-methyltransferase Plm41, and the incorporation of a two-carbon side chain at C4 to generate L-paulomycose (Fig. 7). Two enzymes from the pathway with high similarity to pyruvate dehydrogenase E1 α and β subunits, Plm8 and Plm9, might participate in the incorporation of such side chain. A similar mechanism has been shown to participate in the biosynthesis of kosinostatin [37], it has been biochemically characterized in yersinose A biosynthesis [38], and it has been genetically elucidated in avilamycin A pathway [39]. In all those cases the mechanism involves the transfer of a two-carbon side chain into the deoxysugar from pyruvate. All enzymes involved in L-paulomycose biosynthesis mentioned in this section present orthologues into paulomycin biosynthesis clusters in *S. paulus* and *Streptomyces* sp.YN86 (Table 1).

**Fig. 10:**
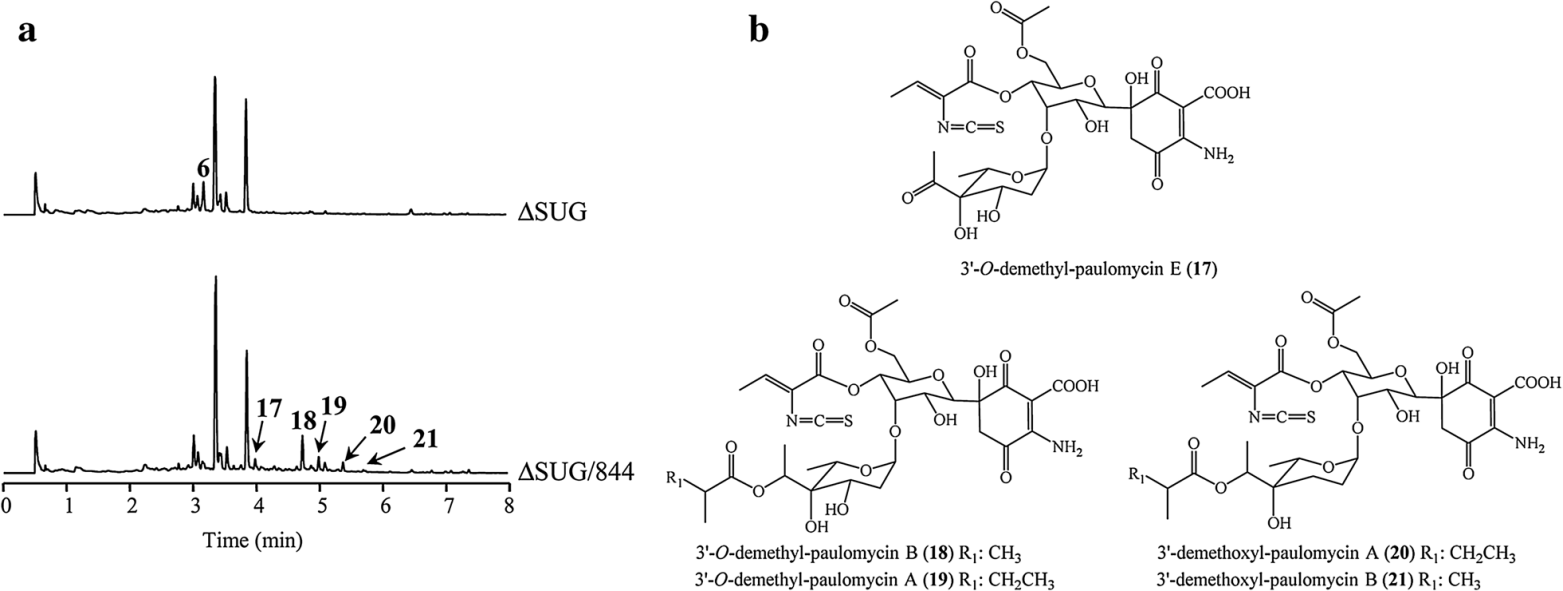
Generation of novel paulomycin derivatives. **a** UPLC chromatograms, monitored at 244 nm, of *S. albus* J1074 mutant strain ∆SUG, altered in L-paulomycose biosynthesis by deletion of *plm40*, *plm41* and *plm42*, and ∆SUG strain carrying pFL844T (∆SUG/844). **b** Chemical structures of novel paulomycin derivatives

An *S. albus* J1074 mutant strain (∆SUG) was generated by simultaneous deletion of three genes that might be involved in L-paulomycose biosynthesis: *plm40*, *plm41* and *plm42*, encoding a dTDP-4-keto-6-deoxy-L-hexose 2,3-reductase, a dTDP-6-deoxy-L-hexose 3-*O*-methyltransferase and a dTDP-4-keto-6-deoxyhexose 3,5-epimerase, respectively. As expected, UPLC analysis of the products accumulated by ∆SUG (Fig. 10a) showed it was unable to produce paulomycins A or B but that produced instead 6-hydroxyl-paulinone (**6**) (Fig. 5). This compound has been previously observed in SAM5324 mutant strain (Fig. 4) that lacks L-paulomycose glycosyltansferase Plm12.

∆SUG mutant strain was used as recipient to host plasmid pFL844T [40] that contains all genes required for the biosynthesis of L-amicetose and L-olivose. Plasmid pFL844T contains, in addition, a 3-*O*-methyltransferase coding gene (*oleY*). OleY is able to introduce an *O*-methyl group into L-olivose and other deoxysugar moieties [41]. The resultant strain ∆SUG/844 showed the appearance of five new UPLC peaks (Fig. 10a), with retention times of 4.0 min (**17**), 4.7 min (**18**), 4.9 min (**19**), 5.3 min (**20**) and 5.7 min (**21**). These compounds showed the characteristic paulomycin absorption spectrum with maxima at 236, 275 and 323 nm, and possessed masses of *m/z*, 687 [*M* + H]^+^ (**17**), 759 [*M* + H]^+^ (**18**), 773 [*M* + H]^+^ (**19**), 757 [*M* + H]^+^ (**20**) and 743 [*M* + H]^+^ (**21**). NMR characterization of these paulomycin derivatives (Additional file 3: Figures S62–S95, Tables S9–S10) showed they correspond to: 3′-*O*-demethyl-paulomycin E (**17**), 3′-*O*-demethyl-paulomycin B (**18**), 3′-*O*-demethyl-paulomycin A (**19**), 3′-demethoxyl-paulomycin A (**20**) and 3′-demethoxyl-paulomycin B (**21**) (Fig. 10b). All these compounds are novel derivatives of paulomycin A, B and E with modifications at the C3′ position of the deoxysugar, lacking the methoxy group (compounds **20** and **21**) or the *O*-methyl group (compounds **17**, **18** and **19**). Curiously, no compounds that present a 3′-*O*-methylation, showing the effect of OleY, have been identified. *O*-methyltransferase OleY has been previously reported to methylate the C3′ hydroxyl group of different deoxysugars, including L-olivose, when attached to macrolide or anthracycline type compounds [41, 42].

### Biological activity of novel paulomycins

The antibacterial activity of paulomycin A (**1**), paulomycin B (**2**), paulomycin E (**3**), paulomycin F (**12**), 13-*O*-deacetyl-13-*O*-paulyl-paulomycin E (**14′**), 13-*O*-deacetyl-13-*O*-paulyl-paulomycin B (**15′**), 13-*O*-deacetyl-13-*O*-paulyl-paulomycin A (**16′**), 3′-*O*-demethyl-paulomycin B (**18**), 3′-*O*-demethyl-paulomycin A (**19**), 3′-demethoxyl-paulomycin A (**20**) and 3′-demethoxyl-paulomycin B (**21**) was monitored against Gram-positives *Micrococcus luteus*, *Staphylococcus aureus*, *S. epidermidis*, and *Streptococcus agalactiae*, and Gram-negatives *Escherichia coli*, *Pseudomonas aeruginosa*, *Serratia marcenscens* and *Klebsiella pneumonia*.

Paulomycins A (**1**), B (**2**), E (**3**) and F (**12**) showed a good antibacterial activity against all Gram-positive bacteria tested, being more active against *S. agalactiae* with inhibition halos of 21, 21, 19 and 18 mm, respectively (Additional file 4: Figure S96). Paulomycin derivatives carrying modifications in the L-paulomicose moiety (compounds **18**, **19**, **20** and **21**) retain antibacterial activity but were less active than paulomycins A and B. The best activity observed was also against *S. agalactiae*, being the most active 3′-*O*-demethyl-paulomycin A (**19**) with an inhibition halo of 20 mm. Paulomycin derivatives carrying an acyl migration of paulic acid to the C13 hydroxyl group (compounds **14′**, **15′** and **16′**) showed no antibacterial activity against the Gram-positive bacteria tested (Additional file 4: Figure S96). All paulomycins and their derivatives were found inactive against the all Gram-negative bacteria tested.

## Discussion

Genome mining of *S. albus* J1074 chromosome sequence predicted the presence of 27 gene clusters putatively involved in secondary metabolites biosynthesis [14]. Five of these clusters were found to direct the biosynthesis of different metabolites: blue pigment indigoidine, polycyclic tetramate macrolactam 6-*epi*-alteramides, polyene candicidins, non-ribosomal peptide antimycins and glycosylated antibiotic paulomycins [14]. Paulomycin biosynthesis gene cluster was initially reported to comprise a region of approximately 60 kb from *sshg_05313* (*plm1*) to *sshg_05354* (*plm42*) based on in silico analysis [14]. These limits have been confirmed in this work by analysis of gene expression and gene inactivation experiments. Production of paulomycins by *S. albus* J1074 was previously reported to be very variable and a reproducible pattern for production of these compounds was not achieved using different batches of media or changing the culture conditions [14]. Furthermore, paulomenols, initially proposed to be paulomycins intermediates [23], were found to be paulomycins degradation products [14]. More recently, additional biosynthesis gene clusters involved in paulomycins biosynthesis have been reported at *S. paulus* NRRL8115 and *Streptomyces* sp. YN86, and a putative biosynthesis pathway has been proposed base on in silico analysis [33].

In addition to a clear instability of paulomycins, which turn into paulomenols by loss of the paulic acid moiety [14], paulomycins intermediates also showed structural instability since products expected to be accumulated by mutant strains generated in this work were mostly degraded or modified into shunt products. According to their respective accumulated products, these mutant strains can be divided in two groups: (i) one group including mutant strains affected in steps occurring before paulic acid incorporation (SAM5331, SAM5335, SAM5340 and SAM5341) and (ii) a second group including mutant strains defective in enzymes acting in late steps once paulic acid has been incorporated into the corresponding intermediate (SAM5315, SAM5324, SAM5334 and ∆SUG). On the basis of all these mutants a pathway for paulomycins biosynthesis is proposed (Fig. 7).

The first set of mutant strains are affected in genes coding for: acyltransferase Plm19, which we propose to be involved in the incorporation of paulic acid while Li and coworkers [33] proposed to be performed by 3-oxoacyl-ACP synthase III Pau29 (Plm27); *C*-glycosyltrasferase Plm23; sulfotransferase Plm28; and aminotransferase Plm29, the last two enzymes likely involved in paulic acid biosynthesis. These mutants accumulate shunt products that do not contain paulic acid: 2-aminobenzoic acid (**8**), *N*-acetyl-*orto*-aminobenzoic (**10**) and deoxydehydrochorismic acid (**11**). 2-aminobenzoic acid (anthranilic acid, **8**), might derive from DHHA dehydration (Fig. 5). Anthranilic acid should also be the intermediate leading to *N*-acetyl-*orto*-aminobenzoic (**10**) by *N*-acetylation. Aminotransferase Plm29 might be involved in paulic acid biosynthesis and acyltransferase Plm19 should transfer the paulic acid moiety to an early paulomycin intermediate in form of *C*-glycosylated quinone (Fig. 7). This putative glycosylated intermediate has not been identified in the aminotransferase and acyltransferase mutants. Perhaps it is unstable and therefore leads to the accumulation of early precursors that are then transformed into anthranilic acid (**8**) and *N*-acetyl-*orto*-aminobenzoic (**10**). The fact that inactivation of *plm23* led also to accumulate *N*-acetyl-*orto*-aminobenzoic (**10**) points to Plm23 *C*-glycosyltransferase acting at early stages of paulomycin biosynthesis on a quinone intermediate that has not been detected in SAM5322 mutant strain. Thus, this unidentified quinone intermediate might, in addition, be unstable (Fig. 5). The same interpretation can be applied to the accumulation of deoxydehydrochorismic acid (**11**), probably generated by chorismate dehydration, by SAM5335 mutant strain. Other compounds not characterized, such as **9**, produced by SAM5331, SAM5341 and SAM5335 strains, shared similar absorption spectrum to *N*-acetyl-*orto*-aminobenzoic (**10**), 2-aminobenzoic acid (anthranilic acid) (**8**) and deoxydehydrochorismic acid (**11**) and possess masses lower than 150 Daltons pointing to possible modification of other paulomycin intermediates such as 2-amino-2-deoxyisochorismate (ADIC) or 3-hydroxyanthranilic acid (3-HAA) (Figs. 5, 7).

The second set of mutant strains mentioned above are defective at: acyltransferase Plm3, responsible for conversion of paulomycin F (**12**) into paulomycin A (**1**) and B (**2**) by incorporation of 2-methylbutyrate and isobutyrate, respectively; glycosyltransferase Plm12, involved in incorporation of paulomycose; acyltransferase Plm22 (Pau24), which incorporates acetate into 13-O-deacetyl-paulomycin E (**14**) to generate paulomycin E (**3**) in a different way as it was proposed previously [33] involving the incorporation of an acetyl group into TDP-D-allose prior to its attachment to the quinone moiety; and enzymes involved in L-paulomycose biosynthesis: dTDP-4-keto-6-deoxy-L-hexose 2,3-reductase Plm40, dTDP-6-deoxy-L-hexose 3-*O*-methyltransferase Plm41, and dTDP-4-keto-6-deoxyhexose 3,5-epimerase Plm42 (Fig. 7). These mutants accumulated compounds containing paulic acid. SAM5324 and ∆SUG mutant strains produce 6-hydroxyl-paulinone (**6**). SAM5315 mutant strain produces intermediate paulomycin F that might derive of paulomycin E by reduction of paulomycose keto group leading to a hydroxyl group (Fig. 5). On the other hand, SAM5334 produces 13-*O*-deacetyl-paulomycin A (**16**) and 13-*O*-deacetyl-paulomycin B (**15**) that might originate from 13-*O*-deacetyl-paulomycin E (**14**) (paulomycin intermediate also identified at SAM5334) by incorporation of 2-methylbutyrate and isobutyrate, respectively (Fig. 5). These results point acyltranferase Plm3 is a flexible enzyme, capable of introducing 2-methylbutyrate and isobutyrate either on paulomycin F (**12**) (Fig. 7) or on13-*O*-deacetyl-paulomycin F (Fig. 8). Furthermore, since 13-*O*-deacetyl-paulomycin E (**14**), 13-*O*-deacetyl-paulomycin A (**16**) and 13-*O*-deacetyl-paulomycin B (**15**) can be converted into paulomycin E, A and B, respectively, acyltransferase Plm22 must also be flexible enough to introduce acetate into those compounds (**14**, **15** and **16**), acting in an alternative pathway (Route B) for the transformation of 13-*O*-deacetyl-paulomycin E (**14**) into paulomycin A (**1**) and B (**2**) (Fig. 8). Compounds **6**, **14**, **15** and **16**, lacking the acetate moiety at C13 hydroxyl group, suffer a paulic acid migration to that position, thus becoming shunt products: 6-hydroxyl-13-*O*-paulyl-paulinone (**6′**), 13-*O*-deacetyl-13-*O*-paulyl-paulomycin E (**14′**), 13-*O*-deacetyl-13-*O*-paulyl-paulomycin B (**15′**) and 13-*O*-deacetyl-13-*O*-paulyl-paulomycin A (**16′**). Migration of acyl groups in aqueous solutions has been demonstrated to occur in other compounds such as betacyanins where glucose 6′-*O*-position is always favored [43], thuggacins that suffer acyl migrations of their lactone group [44], and chloramphenicol that undergoes an intra molecular rearrangement of an acetyl group from 3-hydroxyl to 1-hydroxyl group [45]. It has been demonstrated that acyl migration occurs to primary hydroxyl groups, which is the most stable position for acyl moieties [46, 47]. Stabilization of paulic acid to its natural position at C11 might be determined by transferring acetate at C13 hydroxyl group by acyltransferase Plm22.

Regarding paulic acid biosynthesis, in addition to sulfotransferase Plm28 and aminotransferase Plm29, some other activities are necessary for its biosynthesis, (Fig. 7). However, the lack of compounds containing incomplete versions of paulic acid being produced by SAM5340 or SAM5341 mutants suggest a high specificity of acyltransferase Plm19 for paulic acid and makes characterization of this subpathway a complex issue that might be addressed in a different work. A related issue is paulic acid biosynthetic origin. Based on structural similarities, paulic acid might derive from butyrate or crotonate (Fig. 7). If butyrate corresponds to the real precursor then feeding experiments using valine or isoburyrate should increase the production of all paulomycins (A, B, C and E), but only enhanced production of paulomycin B has been reported [30].

The expression of pFL844T, containing a set of deoxysugar biosynthesis genes for the biosynthesis of L-amicetose and L-olivose, into ∆SUG mutant strain, affected in the biosynthesis of L-paulomycose, led to the generation of new glycosylated forms of paulomycins. The production of such derivatives shows that L-paulomycosyl glycosyltransferase Plm12 possesses a certain degree of flexibility for the transfer of different deoxysugars. In addition, the pyruvate dehydrogenase system form by Plm8 and Plm9 is also flexible to catalyze the attachment of a two-carbon side chain, derived from pyruvate, into both 2,6-dideoxyhexoses and 2,3,6-trideoxyhexoses. On the other hand, *O*-methyltransferase OleY, which has been previously shown to be flexible for modification of deoxysugar moieties attached into macrolide or anthracycline type aglyca [41, 42], is not apparently able to methylate 3′-*O*-demethyl-paulomycin E (**17**), 3′-*O*-demethyl-paulomycin B (**18**) or 3′-*O*-demethyl-paulomycin A (**19**), which will lead to recover the production of paulomycin A, B and E. Bioactivity testing of this paulomycin derivaties showed that the removal of either the L-paulomycose moiety C3′ methoxy group (compounds **20** and **21**) or the *O*-methyl group (compounds **18** and **19**) clearly decreases the antibacterial activity of the compounds with respect to paulomycin A and B. In contrast, the acyl migration of paulic acid to the C13 hydroxyl group due to the absence of the characteristic acetate moiety (compounds **14′**, **15′** and **16′**) render these paulomycin derivatives inactive.

Paulomycin biosynthesis has been shown to be repressed by *afsA*-*y*, γ-butyrolactone synthase gene in *Streptomyces* sp. YN8 [48]. However, there is not an *afsA* homologue present in *S. albus* J1074 or *S. paulus* NRRL 8115 [48], indicating that the upper (pleiotropic) level of paulomycin regulation is strain-specific. Regulation of paulomycins biosynthesis is controlled in *S. albus* J1074 by at least three pathway-specific regulatory systems: LuxR-family Plm2, SARP-family Plm10 and LuxR-family Plm30. Inactivation of genes encoding the last two transcriptional regulators abrogates paulomycin production, while inactivation of *plm2* led to a considerable reduction of paulomycins yields (20 %). On the other hand, *plm1* encoding a TetR-family transcriptional regulator acts as a repressor of the pathway since its inactivation lead to an increased production of paulomycin B and paulomenol B.

## Conclusions

We have unraveled paulomycins biosynthesis pathway by inactivation of genes encoding glycosyltransferases, acyltransferases and enzymes involved in paulic acid biosynthesis. These experiments have allowed the assignment of each of these genes to specific paulomycin biosynthesis steps based on characterization of products accumulated by the corresponding mutant strains. In addition, novel derivatives of paulomycin A, B and E containing L-paulomycose modified moieties were generated by combinatorial biosynthesis. The production of such derivatives shows that L-paulomycosyl glycosyltransferase Plm12 possesses a certain degree of flexibility for the transfer of different deoxysugars. Furthermore, the pyruvate dehydrogenase system formed by Plm8 and Plm9 is also flexible to catalyze the attachment of a two-carbon side chain, derived from pyruvate, into both 2,6-dideoxyhexoses and 2,3,6-trideoxyhexoses. Bioactivity testing of paulomycin derivatives showed that the L-paulomycose moiety C3′ methoxy group is important for the bacterial activity since those compounds lacking this group are less active than their corresponding counterparts. In addition, the paulic acid moiety is not only essential for paulomycins antibacterial activity but also its location is important. The lack of the D-allose acetate moiety and its substitution by a paulic acid moiety renders the corresponding paulomycin derivatives inactive as antibacterial agents.

## Methods

### Strains and culture conditions

Bacterial strains used in this work were *S. albus* J1074 [15] and *S. albus* B29 [14]. *Escherichia coli* DH10B (Invitrogen) and ET12567 (pUB307) [49] were used for subcloning and intergeneric conjugation, respectively. Growth medium for *S. albus* was tryptone soya broth (TSB), MA medium was used for sporulation and R5A as regular production medium [50]. MFE medium (Glucose (10 g/L), Soy bean flour (5 g/L), MOPS (21 g/L), Yeast extract (0.2 g/L), MgSO_4_·7H_2_O (0.6 g/L), K_2_HPO_4_ (1.75 g/L), CaCl_2_ (5 mg/L), MnCl_2_ (1 mg/L), ZnSO_4_ (1 mg/L), FeSO_4_ (5 mg/L), pH 6.8) was used for production and purification of compounds **6**, **12**, **14**, **15,16, 17, 18, 19, 20** and **21**. *E. coli* media were those described in the literature (LB and TB) [51]. When plasmid-containing clones were grown, media were supplemented with appropriate antibiotics: ampicillin (100 µg/mL), tobramycin (20 µg/mL), apramycin (25 µg/mL), thiostrepton (50 µg/mL), tetracycline (10 µg/mL), chloramphenicol (25 µg/mL) and nalidixic acid (50 µg/mL).

### DNA manipulation and plasmids

DNA manipulations were performed according to standard procedures for *E. coli* [51] and *Streptomyces* [49]. PCR conditions used for all amplifications were 99.9 °C for 4 min; 20 cycles of 99.9 °C for 20 s, 65–45 °C touchdown for 20 s and 72 °C for 45 s followed by 10 cycles of 99.9 °C for 20 s, 60 °C for 20 s and 72 °C for 45 s. Final extension was performed at 68 °C for 10 min. Pfx DNA polymerase (Invitrogen) and 2.5 % dimethylsulphoxide (DMSO) were used for all amplifications. PCR products of the expected sizes were initially cloned into pCR-BLUNT for sequencing verification. All oligoprimers used for PCR amplifications are shown in Additional file 1: Table S1. Plasmids used in this work were: pOJ260 [52] for gene disruption; pEFBAoriT [53] and pHZ1358 [54] for gene replacement; pEM4T [55] was used for gene expression; pLHyg [56] was the source of the hygromycin resistance gene *hyg*; and pCR-BLUNT (Invitrogen) was used for cloning PCR products. Plasmid pFL844T [40] was used for the generation of novel paulomycin derivatives by combinatorial biosynthesis.

Methods regarding the construction of plasmids for gene inactivation and ectopic expression and the generation of *S. albus* J1074 mutant strains are provided in Additional file 1. Methods for co-culture experiment are provided in Additional file 2.

### Isolation of total RNA and gene expression analysis

Mycelium from R5A liquid cultures of *S. albus* J1074 was obtained at 48 h following a previously described procedure [57]. Transcript detection analysis was carried out by using the SuperScript one-step RT-PCR with Platinum^®^ Taq DNA polymerase (Invitrogen) with 100 ng of total RNA as a template. Dimethyl sulphoxide (5 % v/v, final) was added to all reactions along with RNAguard RNase inhibitor (32.2 U per reaction) (Amersham Pharmacia Biotech, Europe GmbH, Barcelona, Spain). Conditions were those described before [57] but using specific amplification temperatures depending of each set of primers. Primers listed in Additional file 1: Table S1 were used to generate PCR products of different lengths around 500 bp. Negative controls for each pair of primers were carried out with Platinum^®^ Taq DNA polymerase (Invitrogen) in the absence of reverse transcriptase to confirm that amplified products were not due to the presence of contaminating chromosomal DNA in RNA preparations. Oligonucleotides HRDB-GB1-F and HRDB-GB2-R for *hrdB* [57, 58], encoding a constitutively expressed housekeeping sigma factor, were used as an internal control to normalize RNA samples. RT-PCR analysis were carried out at least three times for each pair of primers and the RT-PCR products were separated in agarose gels and visualized by ethidium bromide staining. Identity of PCR products was verified by direct sequencing with one of the amplification primers.

### Analysis of metabolites by UPLC and HPLC–MS and isolation of compounds

Whole cultures of *S. albus* J1074 and mutants generated in this work were extracted with ethyl acetate containing formic acid (1 %), to enhance the extraction of compounds containing ionizing groups, and analyzed by UPLC and LC–MS for the production of paulomycins, following previously described methods [14, 59]. Reversed phase chromatography was performed in an Acquity UPLC instrument fitted with a BEH C18 column (1.7 µm, 2.1 × 100 mm, Waters). Samples were eluted with 10 % acetonitrile for 1 min, followed by a linear gradient from 10 to 100 % acetonitrile over 7 min, at a flow rate of 0.5 mL/min and a column temperature of 35 °C. For HPLC–MS analysis, an Alliance chromatographic system coupled to a ZQ4000 mass spectrometer and a SunFire C18 column (3.5 µm, 2.1 × 150 mm, Waters) was used. Solvents were the same as above and elution was performed with an initial isocratic hold with 10 % acetonitrile during 4 min followed by a linear gradient from 10 to 88 % acetonitrile over 26 min, at 0.25 mL/min. MS analysis were done by electrospray ionization in the positive mode, with a capillary voltage of 3 kV and a cone voltage of 20 V. Detection and spectral characterization of peaks was performed in both cases by photodiode array detection in the range from 200 to 500 nm, using Empower software (Waters) to extract bidimensional chromatograms at different wavelengths, depending on the spectral characteristics of the desired compound.

Isolation of compounds accumulated by *S. albus* J1074 mutants SAM5315, SAM5324, SAM5331, SAM5334, SAM5335 and ∆SUG, affected in the production of paulomycins was performed following the procedure previously described for isolation of paulomycins [59].

### Structural characterization of compounds

Compounds **6′**, **7**, **8**, **10**, **11**, **12**, **14′**, **15′**, **16′**, **17**, **18**, **19**, **20** and **21** corresponding to 6-hydroxyl-13-*O*-paulyl-paulinone (**6′**), (*2E*)-17-(4′-aminophenyl)-3,11,15-trihydroxy-10,12,14-trimethyl-17-oxo-heptadeca-4,6,8-trienoic acid (**7**), 2-aminobenzoic acid (anthranilic acid) (**8**), *N*-acetyl-*orto*-aminobenzoic acid (**10**), deoxydehydrochorismic acid (**11**), paulomycin F (**12**), 13-*O*-deacetyl-13-*O*-paulyl-paulomycin E (**14′**), 13-*O*-deacetyl-13-*O*-paulyl-paulomycin B (**15′**), 13-*O*-deacetyl-13-*O*-paulyl-paulomycin A (**16′**), 3′-*O*-demethyl-paulomycin E (**17**), 3′-*O*-demethyl-paulomycin B (**18**), 3′-*O*-demethyl-paulomycin A (**19**), 3′-demethoxyl-paulomycin A (**20**) and 3′-demethoxyl-paulomycin B (**21**) were subjected to LC/ESI-TOF analysis in order to determine their molecular formula. The structural elucidation of compounds **6′**, **7**, **8**, **10**, **11**, **12**, **14′**, **15′**, **16′**, **17**, **18**, **19**, **20** and **21** was carried out by analysis of a combination of 1D (^1^H and ^13^C), and 2D (^1^H-^1^H COSY, TOCSY, ^1^H-^13^C heteronuclear single-quantum correlation (HSQC)-edited and ^1^H-^13^C heteronuclear multiple-bond correlation (HMBC) NMR experiments and comparison of the spectra obtained with those described in the literature (supporting information). Solvents used in the NMR analyses were deuterated methanol (CD_3_OD) for compounds **8**, **10** and **11**, or deuterated DMSO (DMSO-*d*_*6*_) for compounds **6′**, **7**, **12**, **14′,15′**,**16′**, **17**, **18**, **19**, **20** and **21** (Additional file 3).

Methods regarding analysis of the antibacterial activity of paulomycins are provided in Additional file 4.

## Additional files

10.1186/s12934-016-0452-4 Methods. Construction of plasmids for gene inactivation and ectopic expression. Methods. Generation of *S. albus* J1074 mutant strains.** Table S1**. Primers used in this work.** Figure S1**. PCR analysis of SAM5355, SAM5312 and SAM5313 mutant strains.** Figure S2**. PCR analysis of SAM5314, SAM5315 and SAM5322 mutant strains.** Figure S3**. PCR analysis of SAM5324, SAM5331, SAM5334 and SAM5335 mutant strains.** Figure S4**. PCR analysis of SAM5340, SAM5341, SAM5342 and DSUG mutant strains.** Figure S5**. Genetic complementation of *S. albus* J1074 mutant strains. Format: PDF.
10.1186/s12934-016-0452-4 Methods. Co-culture experiments.** Figure S6**. Co-culture of SAM5335 and SAM5324 mutant strains.** Figure S7**. Co-culture of SAM5335 and SAM5334 mutant strains. Format: PDF.
10.1186/s12934-016-0452-4 Methods. LC/ ESI-TOF and NMR analyses and structural characterization of: 6-hydroxyl-13-*O*-paulyl-paulinone (6’) [**Figures S8**-**S14**, and** Table S2**]; (2E)-17-(4’-aminophenyl)-3,11,15-trihydroxy-10,12,14-trimethyl-17-oxo-heptadeca-4,6,8-trienoic acid (7) [**Figures S15**-**S19**, and** Table S3**]; 2-aminobenzoic acid (anthranilic acid) (8) [**Figures S20**-**S23**, and** Table S4**]; *N*-acetyl-*orto*-aminobenzoic acid (10) [**Figures S24**-**S26**]; deoxydehydrochorismic acid (11) [**Figures S27**-**S29**]; paulomycin F (12) [**Figures S30**-**S37**, and** Table S5**]; 13-*O*-deacetyl-13-O-paulyl-paulomycin E (14’) [**Figures S38**-**S45**, and** Table S6**]; 13-*O*-deacetyl-13-*O*-paulyl-paulomycin B (15’) [**Figures S46**-**S53**, and** Table S7**]; 13-*O*-deacetyl-13-*O*-paulyl-paulomycin A (16’) [**Figures S54**-**S61**, and** Table S8**]; 3’-*O*-demethyl-paulomycin E (17) [**Figures S62**-**S67**]; 3’-*O*-demethylpaulomycin B (18) [**Figures S68**-**S74**, and** Table S9**]; 3’-*O*-demethyl-paulomycin A (19) [**Figures S75**-**S81**, and** Table S10**]; characterization of 3’-demethoxyl-paulomycin A (20) [**Figure S82**-**S88**, and** Table S11**]; and 3’-demethoxyl-paulomycin B (21) [**Figure S89**-**S95**, and** Table S12**]. Format: PDF.
10.1186/s12934-016-0452-4 Methods. Bioactivity testing of paulomycins.** Figure S96**. Antibacterial activity test of paulomycins and their derivatives. Format: PDF.
