# Supplementary material for: New insights into paulomycin biosynthesis pathway in Streptomyces albus J1074 and generation of novel derivatives by combinatorial biosynthesis
Source: Microb Cell Fact. 2016 Mar 21;15:56. doi: 10.1186/s12934-016-0452-4 (PMC4802897; doi:10.1186/s12934-016-0452-4)

Construction of plasmids for gene inactivation and ectopic expression

Gene disruption of *sshg_05355* was accomplished using plasmid pOJ5355. For this purpose an *sshg_05355* internal fragment of 1 kb bp was amplified by PCR using primers 5355FW and 5355RV and *S. albus* J1074 chromosomal DNA as a template. The resultant fragment was digested EcoRI-HindIII and cloned into pOJ260 digested with the same enzymes.

Deletion of *sshg_05312* was accomplished by amplification of two DNA fragments of 2.7 and 2.6 kb, respectively. Fragment A, amplified using oligoprimers 5309RplFW and 5312RplRV, was cloned into SpeI-NsiI digested pEFBAoriT leading to pEFBA5312-1. Fragment B, amplified using oligoprimers 5312RplFW and 5315RplRV, was cloned as a BamHI-HpaI fragment into BamHI-EcoRV digested pEFBA5312-1, leading to pEFBA12AG. In this construction fragments A and B are flanking the apramycin resistance gene *aac(3)IV*. Finally, pEFBA12AG was digested with XbaI, and *hyg* from pLHyg was subcloned as a SpeI-NheI fragment to obtain plasmid pEFBA12AGH. The same approach was used for deletion of the following genes, using in each case the corresponding restriction enzymes incorporated into the oligoprimers specified at Table S1: fragments of 2.7 and 2.6 kb, respectively, were amplified for deletion of *plm1* using oligoprimer pairs 5310RplFW/5313RplRV and 5313RplFW/5315RplRV, finally leading to construct pEFBA13AGH; fragments of 2.1 and 2.0 kb, respectively, were amplified for deletion of *plm2* using oligoprimer pairs MRA-F/MRA-R and MRB-F/MRB-R, finally leading to construct pEFBATHMRAB; deletion of *plm3* was accomplished by amplifying two fragments of 2.3 and 2.2 kb using oligoprimer pairs 5312RplFW/5315RplRV and 5315RplFW/5318RplRV, to obtain pEFBA15AGH; fragments of 2.0 kb each for deletion of *plm10* were amplified using oligoprimer pairs MRS1-FW/MRS1-RV and MRS2-FW/MRS2-RV, finally

leading to construct pEFBA22AGH; fragments of 2.0 kb each for deletion of *plm12* were amplified using oligoprimer pairs MR1F/MR1R and MR2F/MR2R, finally leading to construct pEFBATHMR12; *plm19* was deleted by amplifying two fragments of 2.1 and 2.2 kb, respectively, using oligoprimer pairs 5333RplRV/5331RplFW and 5331RplRV/5329RplFW, leading to pEFBA31AGH; *plm22* was deleted by amplifying two fragments of 2.4 and 2.5 kb, respectively, using oligoprimer pairs 5330RplFW/5334RplRV and 5334RplFW/5337RplRV, leading to pEFBA34AGH; fragments of 2.0 kb flanking *plm23* were amplified using oligoprimer pairs MRG1F/MRG1R and MRG2F/MRG2R, leading to construct pEFBATHMRG12; deletion of *plm28* was obtained using plasmid pEFBA40AGH constructed using two fragments of 2.4 and 2.6 kb amplified using primer pairs Rpl5338FW/Rpl5340RV and Rpl5340FW/Rpl5342RV; *plm29* was deleted using plasmid pEFBA41AGH constructed using two fragments of 2.2 kb amplified using oligoprimer pairs 5341RplFW/5343RplRV and 5339RplFW/5341RplRV; *plm30* was deleted using plasmid pEFBATHMRL12 constructed using two fragments of 2.0 kb amplified using oligoprimer pairs MRL1-FW/MRL1-RV and MRL2-FW/MRL2-RV; finally, pEFBASUG for deletion of a DNA region containing *plm40*, *plm41* and *plm42* was generated amplifying two fragments of 2.8 and 2.6 kb, respectively, using oligoprimer pairs 5349RplFW/5352RplRV and 5354RplFW/5357RplRV.

Ectopic expression of selected genes (under the control of *ermE*p*), in *S. albus* J1074 or the appropriate mutant strain, was accomplished by PCR amplification of the corresponding genes using oligoprimers specified at Table S1. After verification by sequencing of each PCR fragment, these were subcloned into BamHI-EcoRI or EcoRI digested pEM4T using in each case the appropriate restriction enzymes sites incorporated into the corresponding oligoprimers (Table S1). Final pEM4T-derived

constructs were digested EcoRV and *hyg* was subcloned as an EcoRV fragment obtained from pLHyg. Constructs generated following this procedure were: pEM4HT5314 (to express *plm2*); pEM4HT5315 (*plm3*); pEM4HTSARP (*plm10*); pEM4HT5324 (*plm12*); pEM4HT5331 (*plm19*); pEM4HT5334 (*plm22*); pEM4HT5335 (*plm23*); pEM4HT5340 (*plm28*); pEM4HT5341 (*plm29*) and pEM4HTLuxR (*plm30*).

Generation of *S. albus* J1074 mutant strains

Construct pOJ5355 was introduced into *S. albus* J1074 by intergeneric conjugation form *E. coli* ET12567 (pUB307) leading to mutant strain SAM5355, which was selected by resistance to apramycin. Constructs pEFBA12AGH, pEFBA13AGH, pEFBATHMRAB, pEFBA22AGH, pEFBATHMR12, pEFBATHMRG12, pEFBA15AG, pEFBA31AGH, pEFBA34AGH, pEFBA40AGH, pEFBA41AGH, pEFBATHMRL12, and pEFBASUG were introduced into *S. albus* J1074 by intergeneric conjugation form *E. coli* ET12567 (pUB307) leading to mutant strains SAM5312, SAM5313, SAM5314, SAM5322, SAM5324, SAM5335, SAM5315, SAM5331, SAM5334; SAM5340, SAM5341, SAM5342, and ΔSUG. In all these cases an apramycin and hygromycin resistant mutant, obtained by a single crossover recombination, was grown in the absence of antibiotics and then screened for the lost of hygromycin resistance, keeping apramycin resistance because of a double crossover recombination.

The *S. albus* J1074 mutant strains generated in this work were analyzed by PCR amplification using oligoprimer pairs: 5312MFW/5312MRV (SAM5312); 5313SobFW/5313SobRV (SAM5313); ApraF/5314R (SAM5314); 5314FW/5316RV (SAM5315); 5322ComFW/5322ComRV (SAM5322); ApraF/MR2R (SAM5324); 5331MFW/5331MRV (SAM5331); 5333MFW/5335MRV (SAM5334); ApraF/5335CP

(SAM5335); 5339FW/5341RV (SAM5340); 5341MFW/5341MRV (SAM5341); 5342ComFW/5342ComRV (SAM5342); 5355RV/M13RP and 5355FW/M13FW (SAM5355); and 5352ComFW/5355ComRV (ΔSUG) (Figure S1 to S4). All PCR products generated were cloned into pCR-BLUNT for sequencing verification.

Plasmids pEM4HT5314, pEM4HT5315, pEM4HTSARP, pEM4HT5324, pEM4HT5331, pEM4HT5334, pEM4HT5335, pEM4HT5340 pEM4HT5341 and pEM4HTLuxR were introduced in *S. albus* J1074 mutant strains SAM5314, SAM5315, SAM5322, SAM5324, SAM5331, SAM5334, SAM5335, SAM5340, SAM5341 and SAM5342, respectively, by intergeneric conjugation and exconjugants were selected for resistance to apramycin and hygromycin. Production of paulomycins in all complemented mutant strains was evaluated by UPLC (Figure S5).

Table S1. Primers used in this work. Amplified PCR products were used in gene inactivation, gene expression and RT-PCR studies.

**Primer**	**Sequence (5´-3´)**	**Description**
5355FW	A**GAATTC**ACGATCAACGCCCTCAACTCCG	EcoRI[a,b]
5355RV	AAT**AAGCTT**GTAGCGGATGCCGACCTGG	HindIII[a,b]
5309RplFW	G**ACTAGT**AAGAAATCTCCCGGCGGATG	SpeI[a]
5312RplRV	AAT**ATGCAT**GGTGGCGGTGACTTGGCT	NsiI[a]
5312RplFW	AT**GGATCC**GGCATCTGGGGTGAGGC	BamHI[a]
5315RplRV	GC**GTTAAC**ACAAGGACCACGAGACCTC	HpaI[a]
5310RplFW	GC**ACTAGT**GTCCTCTACCTCGTCTTCAA	SpeI[a]
5313RplRV	GTT**ATGCAT**TGTACCGACCCAGTCCTTCT	NsiI[a]
5313RplFW	TATTCTA**CATATG**ACCAAGTCGGCGTGATGGA	NdeI[a]
5315RplRV	AG**GTTAAC**CTCGATGAAGGTGTGGACCA	HpaI[a]
MRA-F	GACG**ACTAGT**ATGCTGAGCCAAGTCACCGCCA	SpeI[a]
MRA-R	AATT**ATGCAT**CAACCCGGCACGAGAGGACTGC	NsiI[a]
MRB-F	GGCC**CATATG**GAACCCCTGAGAGAATCGCTTC	NdeI[a]
MRB-R	ATCT**GATATC**CGGAGGCGGTGTCGATCTTCCG	EcoRV[a]
MRS1-FW	AATT**ATGCAT**AGAGTTCGGCGCTGAGCGGTCC	NsiI[a]
MRS1-RV	ATAC**ACTAGT**AACGGATCGTTGCTGATCCCGG	SpeI[a]
MRS2-FW	ACGA**GATATC**CGCTGGTGGCAGAGCTTTACGG	EcorV[a]
MRS2-RV	ATAT**CATATG**GAGGCCATGCTGGCCGCCGACC	NdeI[a]
MR1F	AT**ACTAGT**AGGTATCCGCCCGACCGCGTCACG	SpeI[a]
MR1R	ATA**ATGCAT**ATGGCCGGCGACCCGGACCTCG	NsiI[a]
MR2F	ATTA**GGATCC**CCGCGGGGATACGCGCCGACC	BamHI[a]
MR2R	ACGT**GATATC**TTCGTGCACTACCCCATCGCCG	EcoRV[a]
MRG1F	AT**ACTAGT**CAGCAACGATGCGCTCGGCCACG	SpeI[a]
MRG1R	AT**CCTGCAGG**TTACGGGCGGCGGTTGCCAGC	SbfI[a]
MRG2F	AATT**GGATCC**CGCGATCGCTGGCCGAGGAGGT	BamHI[a]
MRG2R	GCAT**GATATC**TCGCCGAAGCCGACCTAGCCGC	EcoRV[a]
5312RplFW	CC**ACTAGT**CCTTCTTGGCGAAATCTCTGTC	SpeI[a]
5315RplRV	TCC**ATGCAT**CCTCAGCACGAAGAAGAAGCT	NsiI[a]
5315RplFW	TT**AGATCT**TCACGGTCTCCCTCGGCCTG	BglII[a]
5318RplFW	T**GATATC**AACGAGTGCAGGGCCACGCC	EcoRV[a]
5333RplRV	ATC**ACTAGT**ACACCATTCCCGCCGCCA	SpeI[a]
5331RplFW	CT**ATGCAT**GGCTTCATCGGTTCGCAC	NsiI[a]
5331RplRV	AT**GGATCC**CCGACATATCCGCACGCG	BamHI[a]
5329RplFW	CG**GTTAAC**TGATGTGGGCCGCGCGGT	HpaI[a]
5330RplFW	CC**ACTAGT**CTGATCCCATGCACGTGCTG	SpeI[a]
5334RplRV	GGC**ACTAGT**AAAGAATGACACGCCGACGG	SpeI[a]
5334RplFW	TT**AGATCT**TCGGCAGCGGCGGTGGCAAC	BglII[a]
5337RplRV	T**GATATC**CGGTCGGCGCCTTCGGCATG	EcoRV[a]
Rpl5338FW	T**ACTAGT**ACGGCGTTCACCGCGTCTG	SpeI[a]
Rpl5340RV	ATT**ATGCAT**GGTGGGGTCGAGGTCGGTA	NsiI[a]
Rpl5340FW	AT**GGATCC**TGAACCCGGACTGGCACCC	BamHI[a]
Rpl5342RV	TTT**GATATC**TCTGCTGGAGGCGGCGGAT	EcoRV[a]
5341RplFW	GAA**ATGCAT**ATCACCGGGAGAGGCGCC	NsiI[a]
5343RplRV	TC**ACTAGT**GGTAGGGAGCCACGGTCC	SpeI[a]

5339RplFW	AT**GATATC**AGGCTCCGCTCCGGCCAT	EcoRV[a]
5341RplRV	AA**GGATCC**GCGCTGACCGGCGTCC	BamHI[a]
MRL1-FW	ATT**GCATGC**GGCGAGGGCGAAGAGCTGGGCC	SphI[a]
MRL1-RV	GTC**ACTAGT**AGCAGCGCCCGGACGACGTTCG	SpeI[a]
MRL2-FW	AACT**GATATC**GATCGTCGCCTGGGCCGAGCGG	EcoRV[a]
MRL2-RV	ATAT**GGATCC**CGCCCGCACCCCTGAGCTGCTG	BamHI[a]
5349RplFW	AACTAGTCACGCTCGACGGCGCCAA	SpeI [a]
5352RplRV	AACATGCATTCCACCAGTAGGCCGCTG	NsiI [a]
5354RplFW	ATGGATCCCAAAGGCGCGTGCGGAGG	BamHI [a]
5357RplRV	GCGATATCCCCGGACGTAGGTGAAGG	EcoRV [a]
5312MFW	CGTAGCGAGTGCGCGAGCGG	SAM5312[b]
5312MRV	CCGCCGCCTGGGTAATCCTG	SAM5312[b]
5313SobrFW	CCTATACTGGACGCCTCTGCGG	SAM5313[b]
5313SobrRV	GGCAGGGACGGCGGAGAGG	SAM5313[b]
ApraF	TCATCGGTCAGCTTCTCAACCTT	SAM5314[b]
5314R	AAGGATCCATCGAAGTCCGAGCGGACATCG	SAM5314[b]
5314FW	GCCAGCTCCACCAACGCGTC	SAM5315[b]
5316RV	CGGTACCTGCCAGACGCCCA	SAM5315[b]
5322ComFW	CACCGCGAAGGCCAGCCTC	SAM5322[b]
5322ComRV	GAGAACAAGACAGCCGGGGCG	SAM5322[b]
MR2R	ACGTGAATTCTTCGTGCACTACCCCATCGCCG	SAM5324[b]
5331MFW	ATGCGTCCAAGGTGCAGTCCCG	SAM5331[b]
5331MRV	CGGGTTGCCGGCGTAGCTGAGT	SAM5331[b]
5333MFW	AAGCTGGCCGAGGGGCTGTC	SAM5334[b]
5335MRV	GAGACGCACACTCCCGGCAG	SAM5334[b]
5335CP	ATGTTTCCTCTTCGTCACGGGCGG	SAM5335[b]
5339FW	ACAGCCACCCCGTGACCGCG	SAM5340[b]
5341RV	GTCGAGGAGGGACGCTGCGT	SAM5340[b]
5341MFW	GGTGGATTCCTCTTGCCGCTTCC	SAM5341[b]
5341MRV	CCACCGTCCGACCGACTGGAGT	SAM5341[b]
5342ComFW	CGGGTGGTTGGGACGGCTCC	SAM5342[b]
5342ComRV	GCTCTGCTGGAGGCGGCGGA	SAM5342[b]
M13FW	TGTAAAACGACGGCCAGT	SAM5355[b]
M13RP	CAGGAAACAGCTATGACC	SAM5355[b]
5352ComFW	TACCGCGACGAGAGAGGATGTG	ΔSUG[b]
5355ComRV	GACGACTACGCCGACTTCCTCA	ΔSUG[b]
5314SobFW	TA**GGATCC**GCTAGCAGAACACCGCGCCATGAT	BamHI[a] *sshg_05314*[c]
5314SobRV	CG**CAATTG**TCGGTCCGCCTGATGTCCCG	MfeI[a] *sshg_05314*[c]
5315CFW	GT**GAATTC**GTTGATACGTTCGCCGCG	EcoRI[a] *sshg_05315*[c]
5315CRV	T**GAATTC**GGCAGGGACGGCGGAGA	EcoRI[a] *sshg_05315*[c]
SarP2FW	CATGAC**GGATCC**GATTCCCTGAGCGGAGTACG	BamHI[a] *sshg_05322*[c]
SarP2RV	CTAGCA**GAATTC**ACGACATCGCGAACGCCTGT	EcoRI[a] *sshg_05322*[c]
5324CFW	AT**AGATCT**CGTGTCGTGGGCTCCTGC	BglII[a]

		sshg_05324[c]
5324CRV	AC**GAATTC**AGCCGGTCAGCCGCAG	EcoRI[a] *sshg_05324*[c]
5331CFW	AT**GGATCC**TGCCGGCGTAGCTGAGTT	BamHI[a] *sshg_05331*[c]
5331CRV	GGC**GAATTC**GGATATGTCGGAGTTTCTGA	EcoRI[a] *sshg_05331*[c]
5334CFW	CG**GAATTC**GGCAGAGCATACTTTCGC	EcoRI[a] *sshg_05334*[c]
5334CRV	GC**GAATTC**GGATGGACAAGGAACAGG	EcoRI[a] *sshg_05334*[c]
5335FW	AA**GGATCC**ACGAGGCGTCACGCGTTC	BamHI[a] *sshg_05335*[c]
5335RV	GT**GAATTC**TTGCGTGACACCGCCCCC	EcoRI[a] *sshg_05335*[c]
5340CFW	AA**GGATCC**CCTAGGAGACCCTCCATGCCCCTG	BamHI[a] *sshg_05340*[c]
5340CRV	G**GAATTC**GCTAGCGGTGCCAGTCCGGGTTCA	EcoRI[a] *sshg_05340*[c]
5341CFW	AT**GGATCC**CCTAGGACCGAGTGCTCTGGCCT	BamHI[a] *sshg_05341*[c]
5341CRV	T**GAATTC**GCTAGCTCGCTTTCCCGTATTTGCTGGGC	EcoRI[a] *sshg_05341*[c]
LuxRFW	CATGAC**GGATCC**CGTGGCCGCCGACGGACATC	MunI[a] *sshg_05342*[c]
LuxRRV	CTAGCA**CAATTG**GACAGGCGCCGGCCGACCCG	BamHI[a] *sshg_05342*[c]
5312-F	AAGTCACCGCCACCCGCTAC	*sshg_05312*[d]
5312-R	GGAAGTCGTACCGCTTGGTCAC	*sshg_05312*[e]
5313-F	TTGACGGTCGACAGGATTACCC	*sshg_05313*[d]
5313-R	AGCAAATGCAGGCCCTTGATG	*sshg_05313*[e]
5314-F	TGCTCCTGATGTGGTGCTGATG	*sshg_05314*[d]
5314-R	TGATCCGGCGATTCCTGTCTC	*sshg_05314*[e]
5315-F	CTCTGTCCTTCAACGGCGACTG	*sshg_05315*[d]
5315-R	CGGGGAAGAGGTAGACGAACCA	*sshg_05315*[e]
5353-F	CGCGCGAACGGCTGACGAAGTTG	*sshg_05353*[d]
5353-R	GGCTGGTAGAGGTCCAGGTCGAAG	*sshg_05353*[e]
5354-F	GTGTTCACGCCCCAGGTCTTC	*sshg_05354*[d]
5354-R	CGCCAGGCAGGTCTCGTACTC	*sshg_05354*[e]
5355-F	TGGTCGACTTCGGCCTGTACTTC	*sshg_05355*[d]
5355-R	CGGTCCTTGTCGTCCTTGACCT	*sshg_05355*[e]
5356-F	GAGGAAGGGGACCTGTGTCTGC	*sshg_05356*[d]
5356-R	GTCGACGATGTTCGGGTAGGC	*sshg_05356*[e]

[a] Restriction site in bold in primer sequence
[b] Primers to verify mutant strains by PCR
[c] Primers to amplify genes used for ectopic expression
[d] Forward primer used for RT-PCR studies
[e] Reverse primer used for RT-PCR studies

Figure S1. PCR analysis of SAM5355, SAM5312 and SAM5313 mutant strains. Chromosomal DNA from each mutant was used to verify the correct location of the resistance marker. The oligoprimers used are listed at Table S1. Lambda DNA digested with *Pst*I has been used as molecular-weight size marker. All PCR products generated were sequenced.

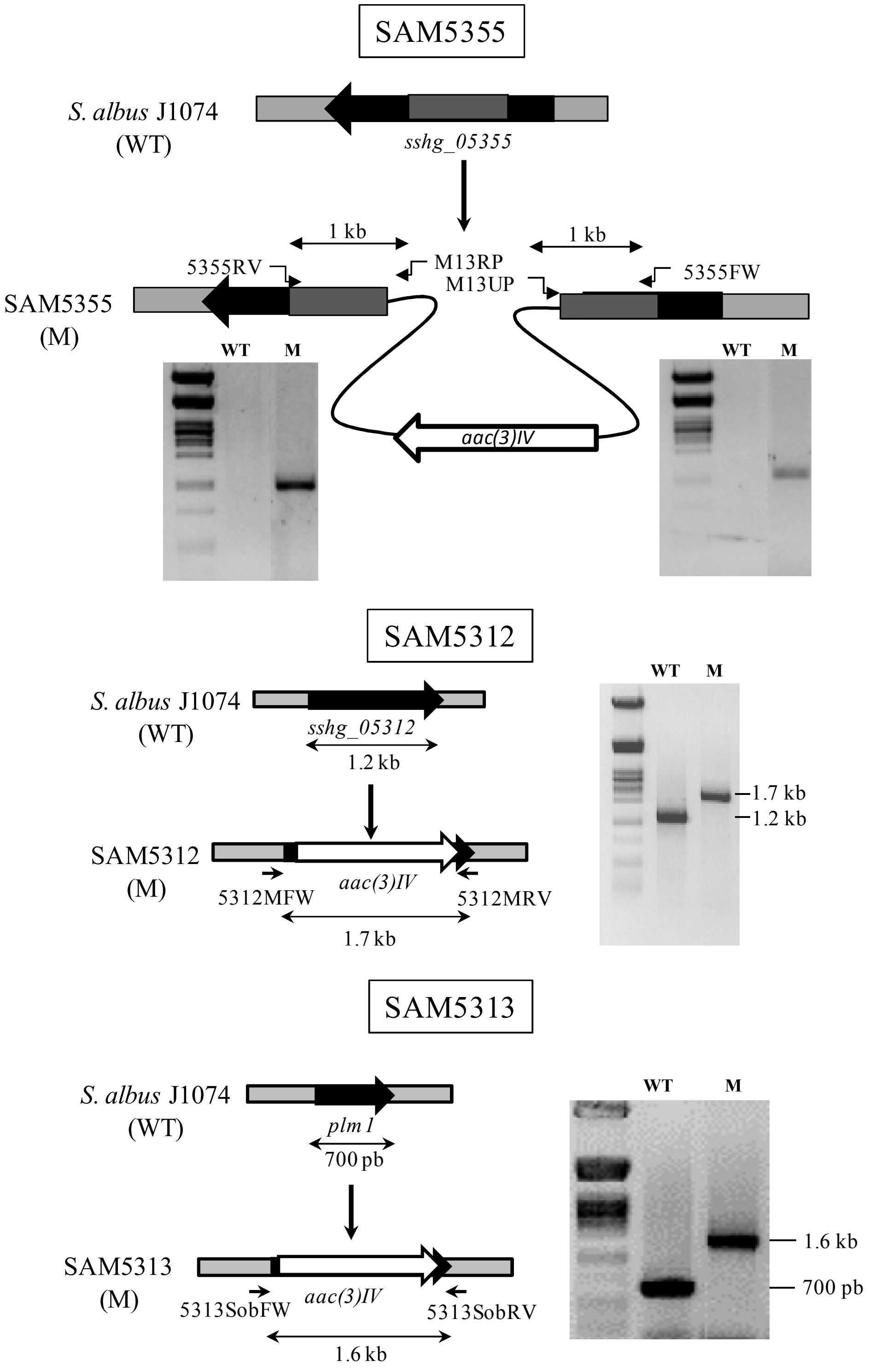

Figure S2. PCR analysis of SAM5314, SAM5315 and SAM5322 mutant strains. Chromosomal DNA from each mutant was used to verify the correct location of the resistance marker. The oligoprimers used are listed at Table S1. Lambda DNA digested with *Pst*I has been used as molecular-weight size marker. All PCR products generated were sequenced.

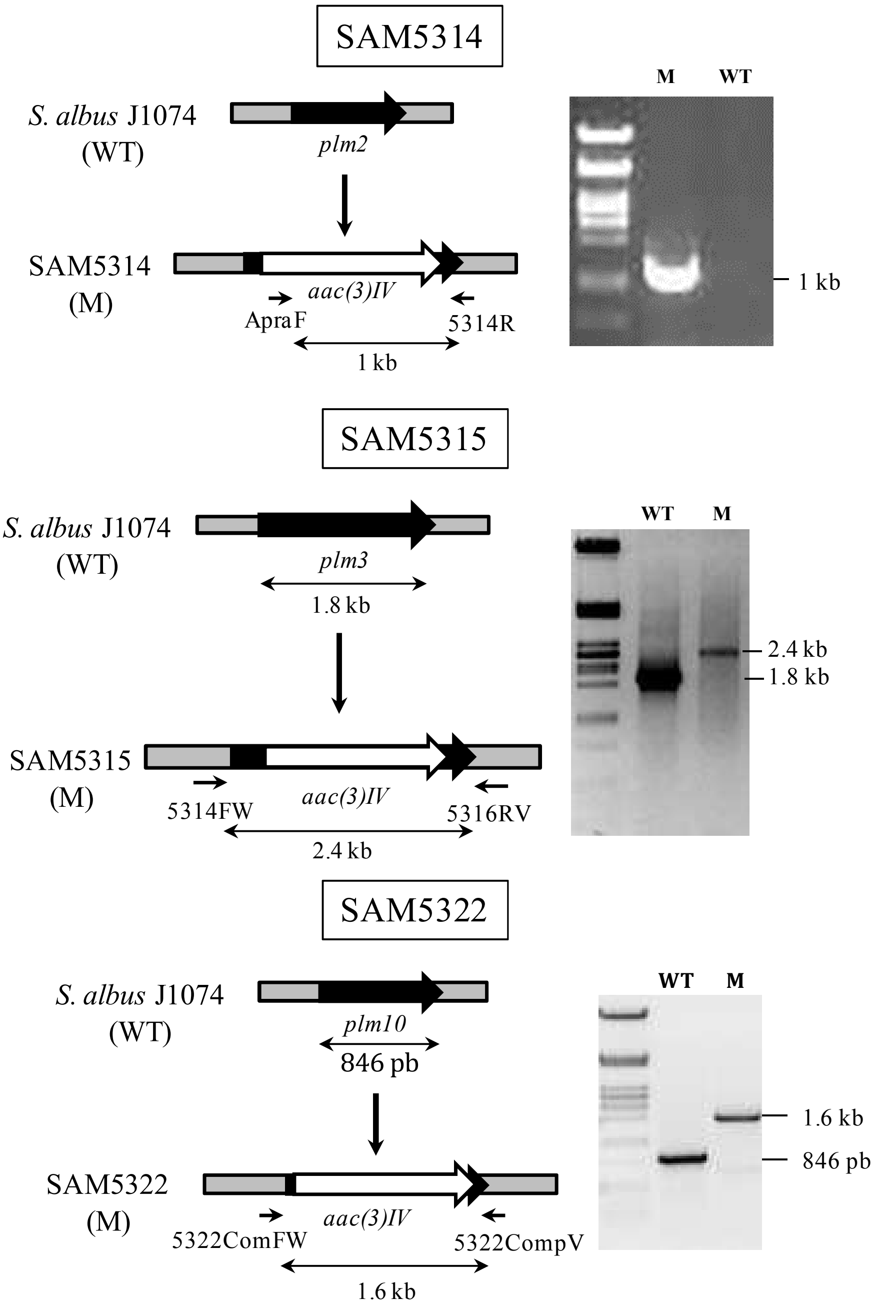

Figure S3. PCR analysis of SAM5324, SAM5331, SAM5334 and SAM5335 mutant strains. Chromosomal DNA from each mutant was used to verify the correct location of the resistance marker. The oligoprimers used are listed at Table S1. Lambda DNA digested with *Pst*I has been used as molecular-weight size marker. All PCR products generated were sequenced.

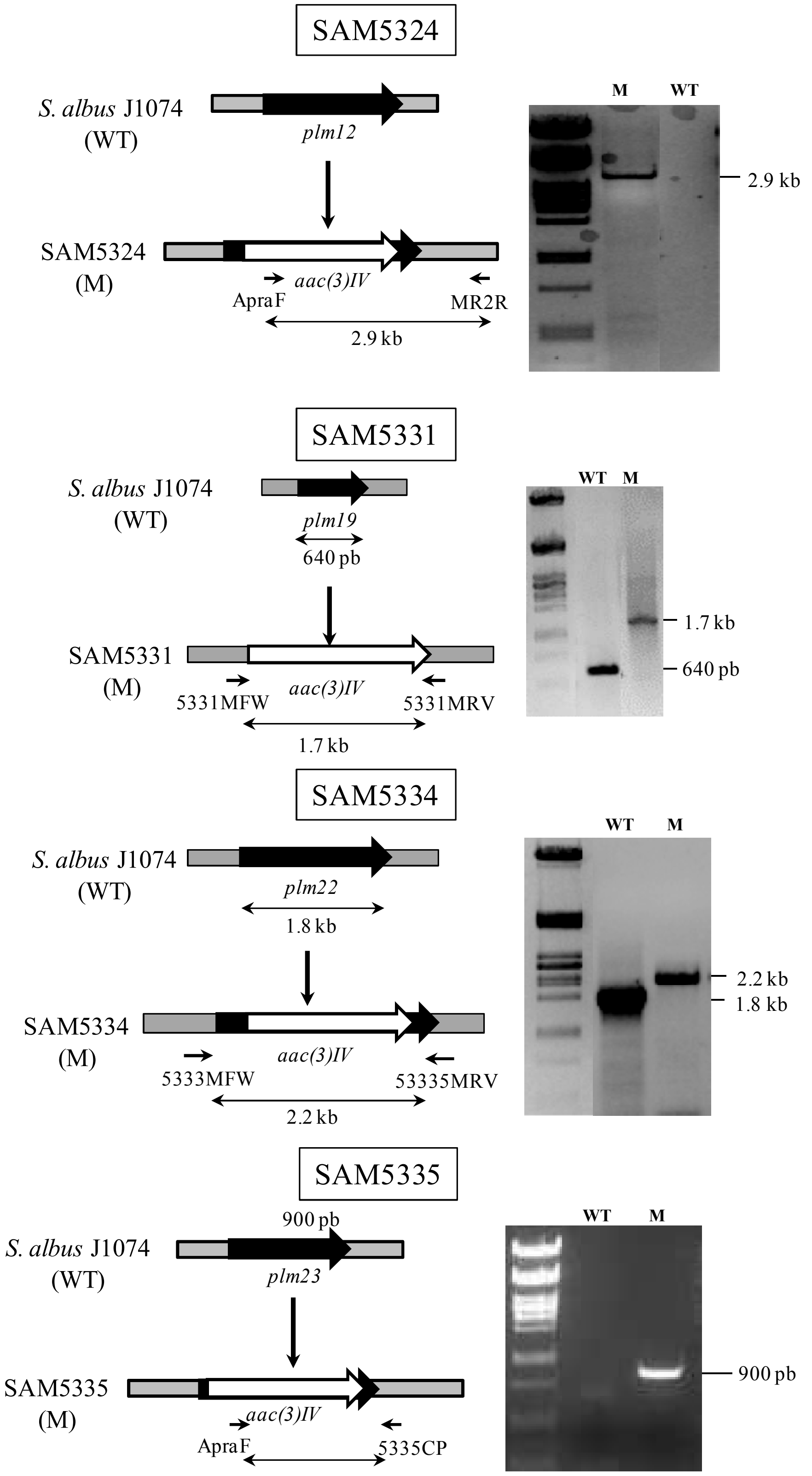

Figure S4. PCR analysis of SAM5340, SAM5341, SAM5342 and ΔSUG mutant strains. Chromosomal DNA from each mutant was used to verify the correct location of the resistance marker. The oligoprimers used are listed at Table S1. Lambda DNA digested with *Pst*I has been used as molecular-weight size marker. All PCR products generated were sequenced.

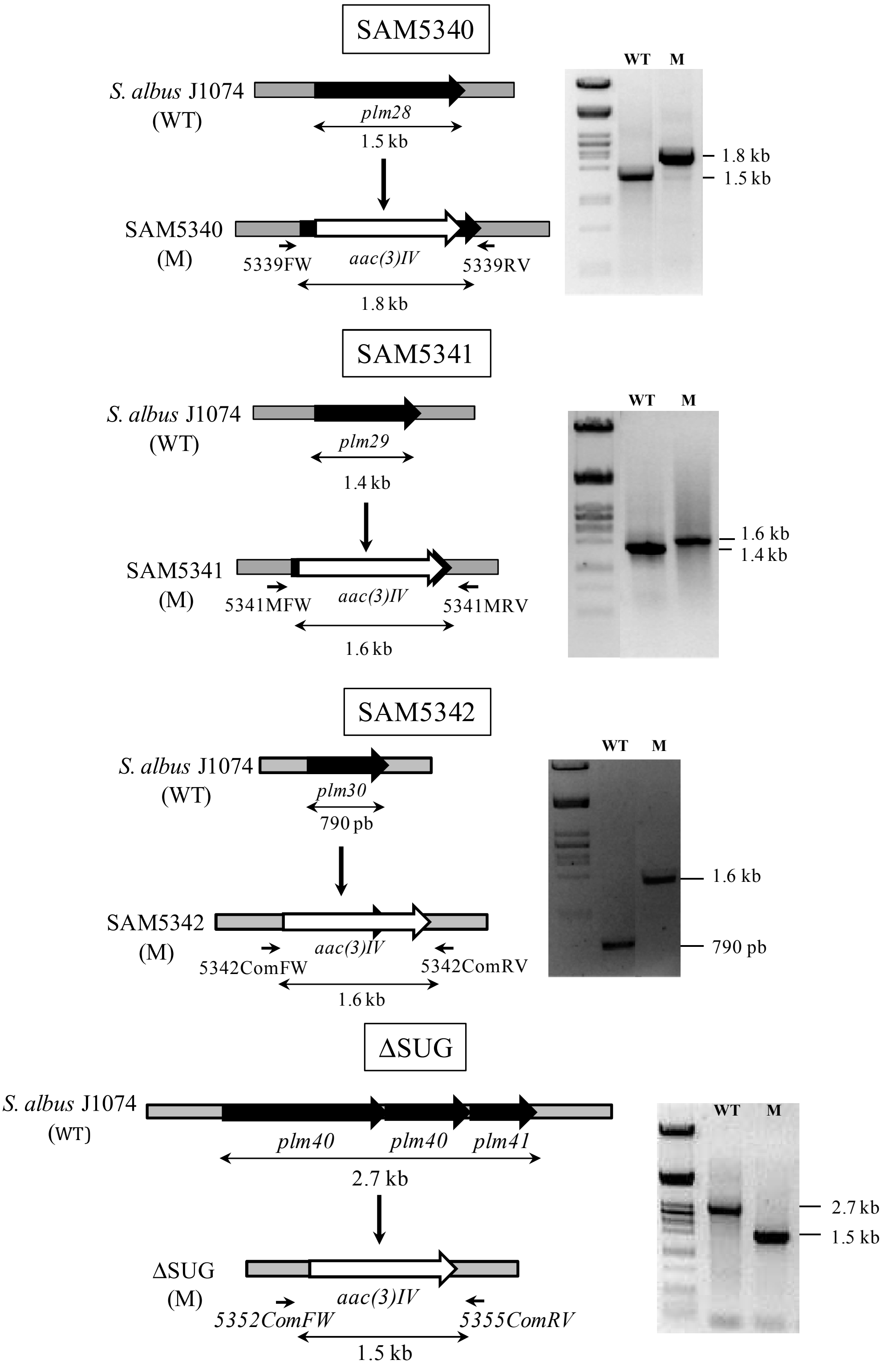

Figure S5. Genetic complementation of *S. albus* J1074 mutant strains. UPLC traces of paulomycins and paulomenols from each mutant strain complemented with the appropriate plasmid. Each plasmid is expressing the appropriate intact gene under the control of *ermE*p*.

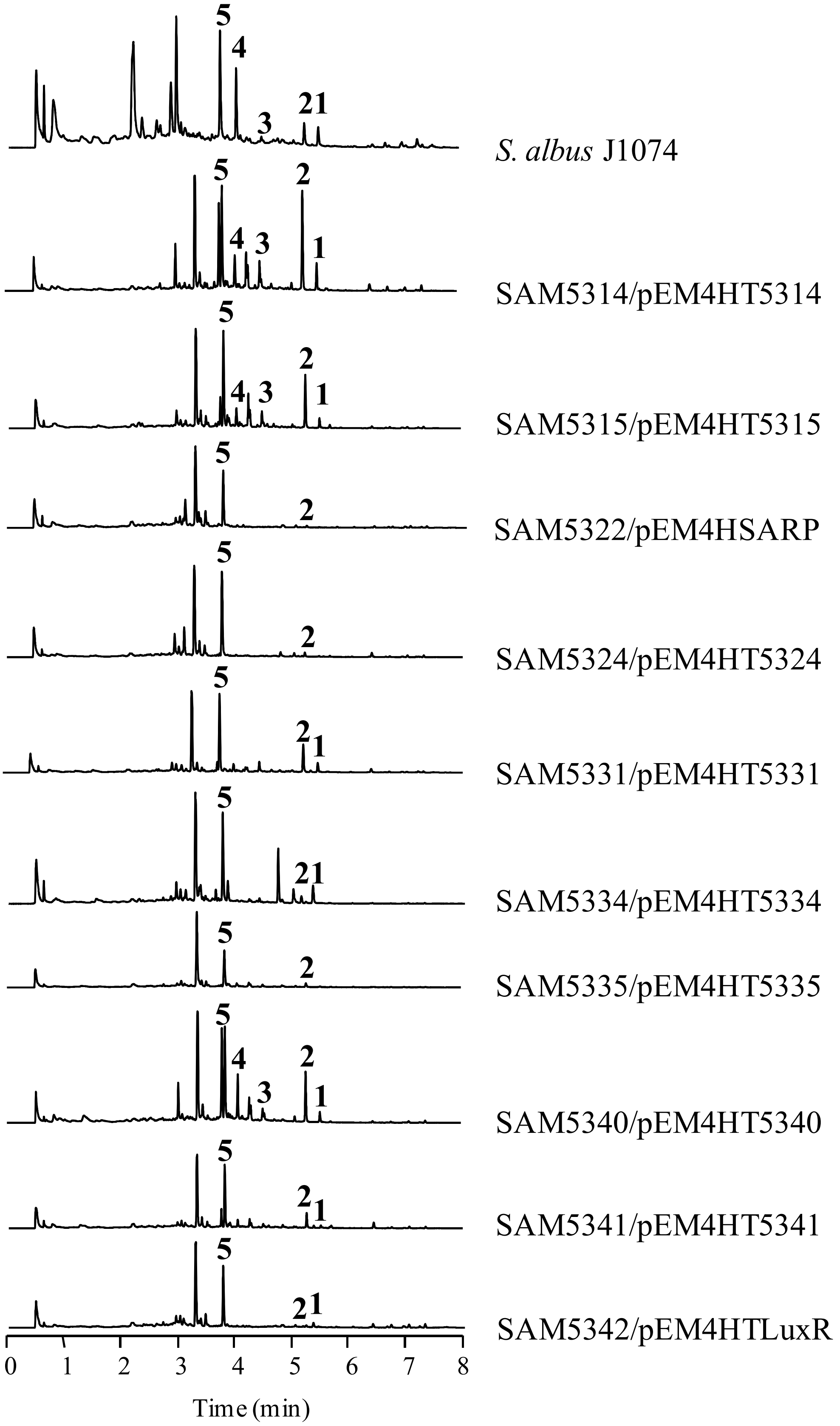

Supplement: Supplementary file 1 — 10.1186/s12934-016-0452-4 Methods. Construction of plasmids for gene inactivation and ectopic expression. Methods. Generation of S. albus J1074 mutant strains. Table S1. Primers used in this work. Figure S1. PCR analysis of SAM5355, SAM5312 and SAM5313 mutant strains. Figure S2. PCR analysis of SAM5314, SAM5315 and SAM5322 mutant strains. Figure S3. PCR analysis of SAM5324, SAM5331, SAM5334 and SAM5335 mutant strains. Figure S4. PCR analysis of SAM5340, SAM5341, SAM5342 and DSUG mutant strains. Figure S5. Genetic complementation of S. albus J1074 mutant strains. Format: PDF. [file 12934_2016_452_MOESM1_ESM.pdf]
