# Supplementary material for: New insights into paulomycin biosynthesis pathway in Streptomyces albus J1074 and generation of novel derivatives by combinatorial biosynthesis
Source: Microb Cell Fact. 2016 Mar 21;15:56. doi: 10.1186/s12934-016-0452-4 (PMC4802897; doi:10.1186/s12934-016-0452-4)
Supplement: Supplementary file 2 — 10.1186/s12934-016-0452-4 Methods. Co-culture experiments. Figure S6. Co-culture of SAM5335 and SAM5324 mutant strains. Figure S7. Co-culture of SAM5335 and SAM5334 mutant strains. Format: PDF. [file 12934_2016_452_MOESM2_ESM.pdf]

Co-culture experiments

For co-culture experiments each strain was grown in Erlenmeyer flasks (250 mL), each containing 50 mL of MFE medium, inoculated with spores and incubated in an orbital shaker (Climo-Shaker ISF4-X, Adolf Kühner AG, Basel, Switzerland) at 30°C and 250 r.p.m. during 2 days. Then, 25 mL of each culture were mixed, transferred to an additional Erlenmeyer flask (250 mL) and culture for 24 additional hours (final 72 hours). Whole mono- and co-cultures were extracted with ethyl acetate containing formic acid (1%) and analyzed by UPLC and LC-MS for the production of paulomycins following previously described methods [1,2].

Figure S6. Co-culture of SAM5335 and SAM5324 mutant strains. The recovery of paulomycin A and B production by transformation of 6-hydroxyl-paulinone (**6**) is shown.

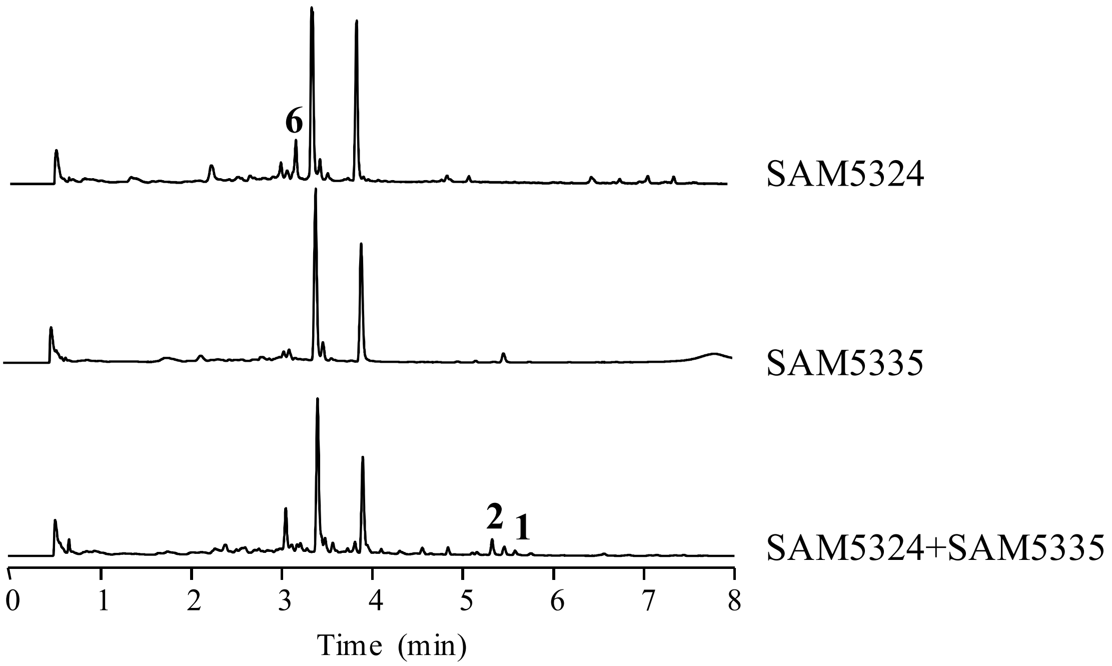

Figure S7. Co-culture of SAM5335 and SAM5334 mutant strains. The recovery of paulomycin A and B production by transformation of 13-*O*-deacetyl-paulomycin E (**14**), 13-*O*-deacetyl-paulomycin B (**15**) and 13-*O*-deacetyl-paulomycin A (**16**) is shown.

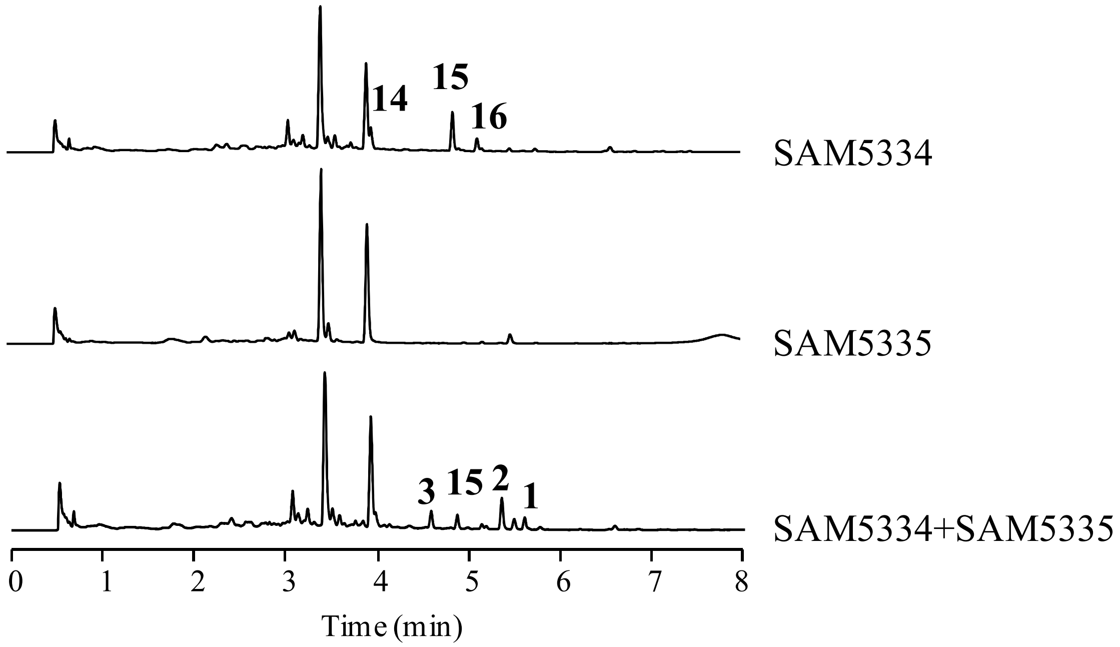

References

1. Braña AF, Rodríguez M, Pahari P, Rohr J, García LA, Blanco G. Activation and silencing of secondary metabolites in *Streptomyces albus* and *Streptomyces lividans* after transformation with cosmids containing the thienamycin gene cluster from *Streptomyces cattleya*. Arch Microbiol. 2014;196:345-355.

2. Olano C, García I, González A, Rodriguez M, Rozas D, Rubio J, et al. Activation and identification of five clusters for secondary metabolites in *Streptomyces albus* J1074. Microb Biotechnol. 2014;7:242-256.
