# Supplementary material for: New insights into paulomycin biosynthesis pathway in Streptomyces albus J1074 and generation of novel derivatives by combinatorial biosynthesis
Source: Microb Cell Fact. 2016 Mar 21;15:56. doi: 10.1186/s12934-016-0452-4 (PMC4802897; doi:10.1186/s12934-016-0452-4)
Supplement: Supplementary file 3 — 10.1186/s12934-016-0452-4 Methods. LC/ ESI-TOF and NMR analyses and structural characterization of: 6-hydroxyl-13-O-paulyl-paulinone (6’) [Figures S8-S14, and Table S2]; (2E)-17-(4’-aminophenyl)-3,11,15-trihydroxy-10,12,14-trimethyl-17-oxo-heptadeca-4,6,8-trienoic acid (7) [Figures S15-S19, and Table S3]; 2-aminobenzoic acid (anthranilic acid) (8) [Figures S20-S23, and Table S4]; N-acetyl-orto-aminobenzoic acid (10) [Figures S24-S26]; deoxydehydrochorismic acid (11) [Figures S27-S29]; paulomycin F (12) [Figures S30-S37, and Table S5]; 13-O-deacetyl-13-O-paulyl-paulomycin E (14’) [Figures S38-S45, and Table S6]; 13-O-deacetyl-13-O-paulyl-paulomycin B (15’) [Figures S46-S53, and Table S7]; 13-O-deacetyl-13-O-paulyl-paulomycin A (16’) [Figures S54-S61, and Table S8]; 3’-O-demethyl-paulomycin E (17) [Figures S62-S67]; 3’-O-demethylpaulomycin B (18) [Figures S68-S74, and Table S9]; 3’-O-demethyl-paulomycin A (19) [Figures S75-S81, and Table S10]; characterization of 3’-demethoxyl-paulomycin A (20) [Figure S82-S88, and Table S11]; and 3’-demethoxyl-paulomycin B (21) [Figure S89-S95, and Table S12]. Format: PDF. [file 12934_2016_452_MOESM3_ESM.pdf]

LC/ ESI-TOF and NMR analyses

LC/ESI-TOF analysis was performed using an Agilent 1200RR HPLC equipped with a SB-C8 column (2.1 × 30 mm, Zorbax) coupled to a a Bruker maXis Spectrometer. Solvent A consisted of 10% acetronitrile and 90% water with 1.3 mM trifluoroacetic acid (TFA) and ammonium formate, and solvent B was 90% acetronitrile and 10% water with 1.3 mM TFA and ammonium formate. The gradient started at 10% B and went to 100% B in 6 minutes, kept at 100% B for 2 minutes and returned to 10% B for 2 minutes to initialize the system. The mass spectrometer was operated in positive ESI mode. The instrumental parameters were: 4kV capillary voltage, drying gas flow of 11 L/ min at 200 ºC, nebulizer pressure at 2.8 bars. TFA-Na cluster ions were used for mass calibration of the instrument prior to sample injection. Pre-run calibration was by infusion with the same TFA-Na calibrant.

NMR spectra were recorded at 24 ºC on a Bruker AVANCE III-500 MHz (500 and 125 MHz for ^{1}H and ^{13}C NMR, respectively) equipped with a 1.7 mm TCI MicroCryoProbe^{TM}, using the signal of the residual solvent as internal reference.

Structural characterization of 6-hydroxyl-13-*O*-paulyl-paulinone (6')

The HRMS data of compound **6'** confirmed a molecular formula of $C_{18}H_{20}N_2O_{11}S$ that was deduced from the pseudo molecular ion at *m/z* 473.0863 $[M+H]^+$ obtained by ESI-TOF analysis (Figure S8). Analyses of correlations observed by 1H NMR spectrum acquired in DMSO-d6 (Figures S9 to S11) were in accordance the initial proposed structured of a paulomycin derivative lacking the 10-*O*-deoxysugar moiety. Chemical shifts observed in COSY, HSQC (Figure S12) and HMBC spectra (Figure S13) confirmed the correlations observed by ^{1}H NMR spectrum and the connectivity and stereochemistry of the polyhydroxylated ring. Furthermore, these results confirmed the

connectivity of paulic acid moiety with the second cycle and the carbon involved in the c-glycoside bond. The ^{1}H-^{13}C HMBC spectrum showed a correlation between the paulic acid carbonyl group and the central ring methylene group indicating the primary hydroxyl group of this ring is acylated. To confirm the structure of **6'** and the acylation on the primary hydroxyl group, its chemical shifts (Table S2) were compared to those reported in the literature for paulomycin A [1,2] and senfolomycinin A [3]. From that comparison was clear that C-11 hydroxyl group was not acylated by paulic acid, being this group esterifed at C-13 hydroxyl group. This allowed identification of **6'** as 6-hydroxyl-13-*O*-paulyl-paulinone (Figure S14).

Figure S8. (+) ESI-TOF spectrum of 6-hydroxyl-13-*O*-paulyl-paulinone (6').

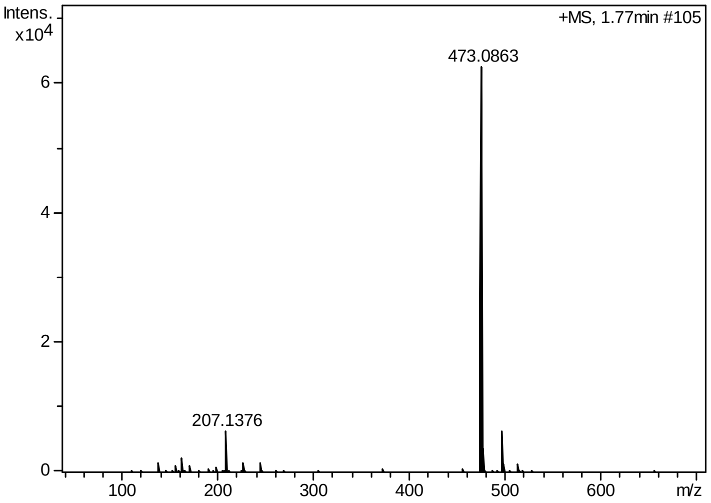

Figure S9. ^{1}H NMR spectrum of 6-hydroxyl-13-*O*-paulyl-paulinone (6’) (DMSO-*d$_6$*, 500 MHz, 24 °C).

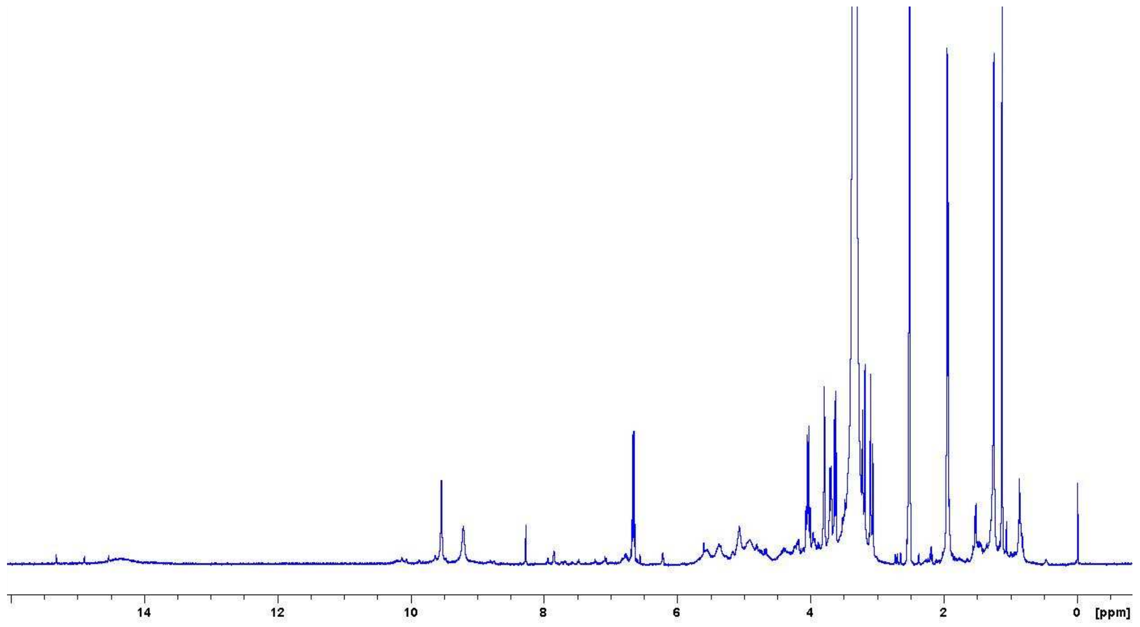

Figure S10. Detail of ^{1}H NMR spectrum of 6-hydroxyl-13-*O*-paulyl-paulinone (6’) (DMSO-*d$_6$*, 500 MHz, 24 °C).

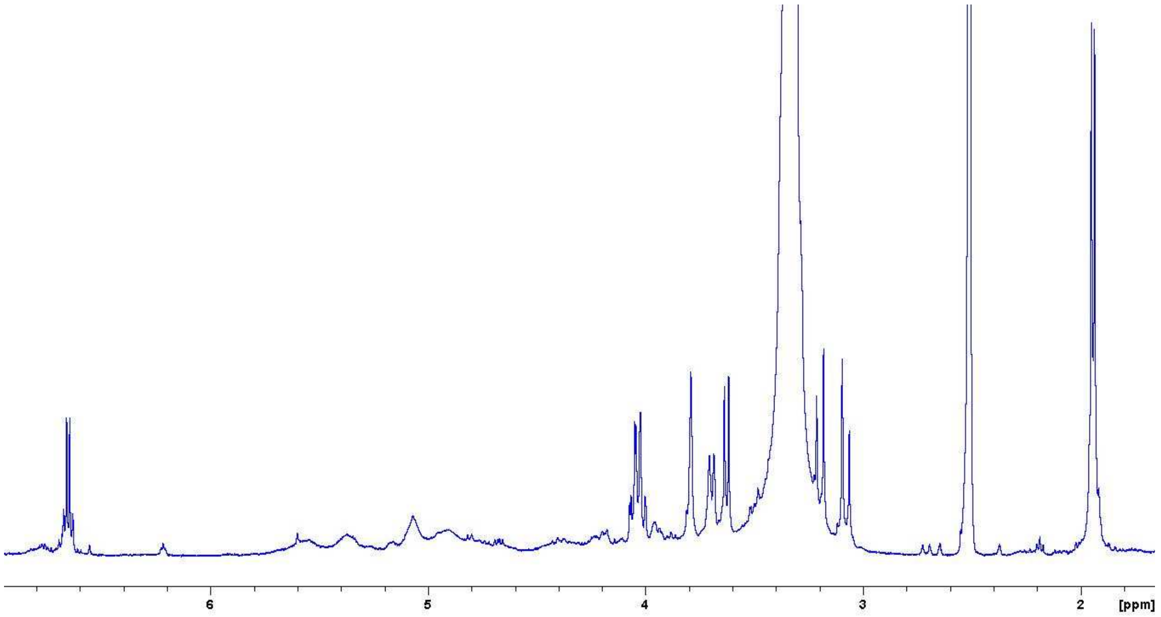

Figure S11. ^{1}H NMR spectrum of 6-hydroxyl-13-*O*-paulyl-paulinone (6') (DMSO-*d_6*, 500 MHz, 24 °C) (in red). The same data edited using a T2 filter (in blue) is shown.

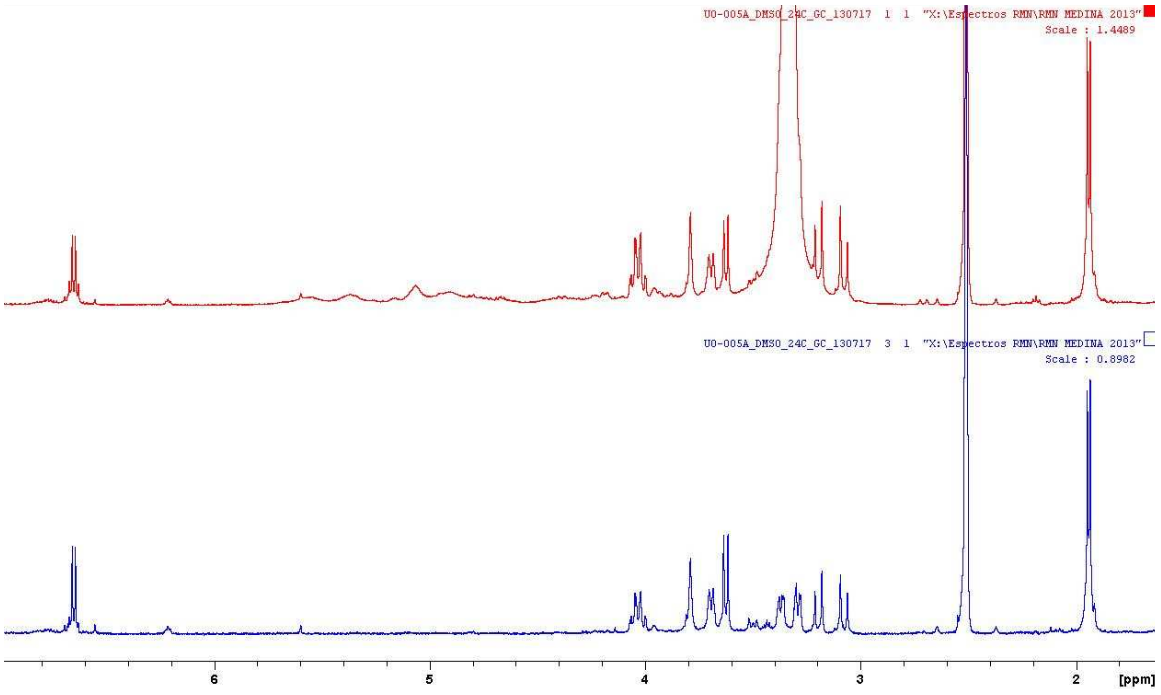

Figure S12. Detail of ^{1}H-^{13}C HSQC spectrum (polyhydroxylated ring) of 6-hydroxyl-13-*O*-paulyl-paulinone (6') in DMSO-*d_6* at 500 MHz.

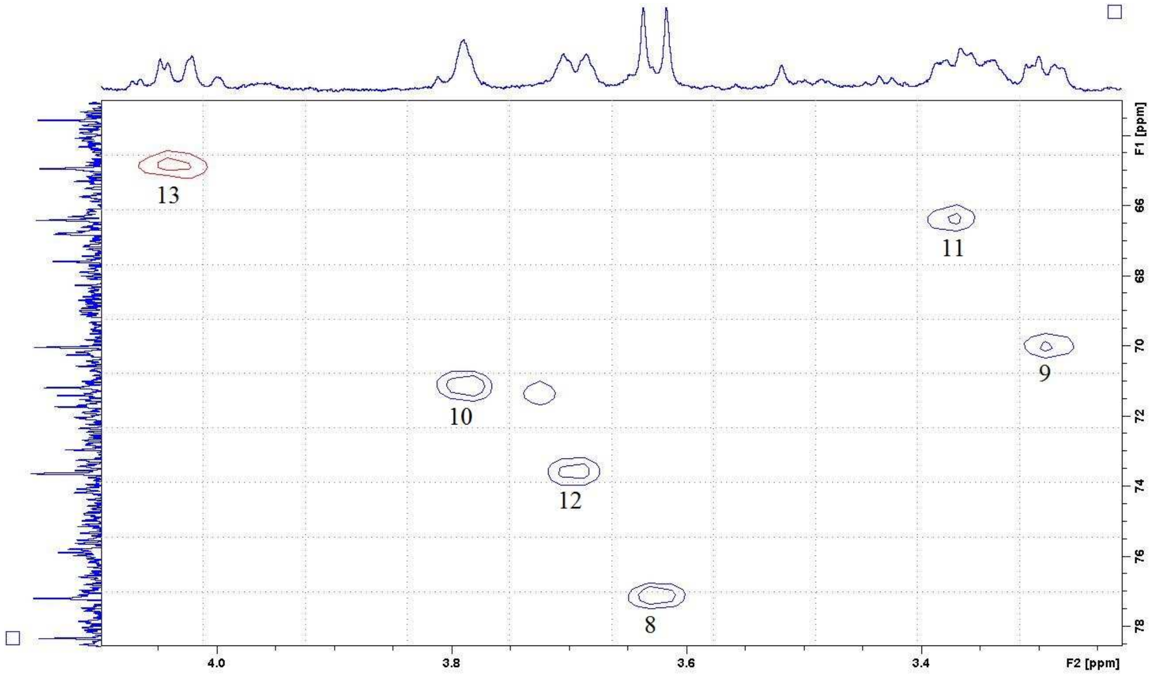

Figure S13. Expansion of the ^{1}H-^{13}C HMBC spectrum (of 6-hydroxyl-13-*O*-paulyl-paulinone (6') in DMSO-d_6 at 500 MHz. The key correlation between carbon 1' and protons at position 13 is highlighted in red color. The other HMBC correlations involving this carbonyl with the protons at positions 3' and 4' are highlighted in green color.

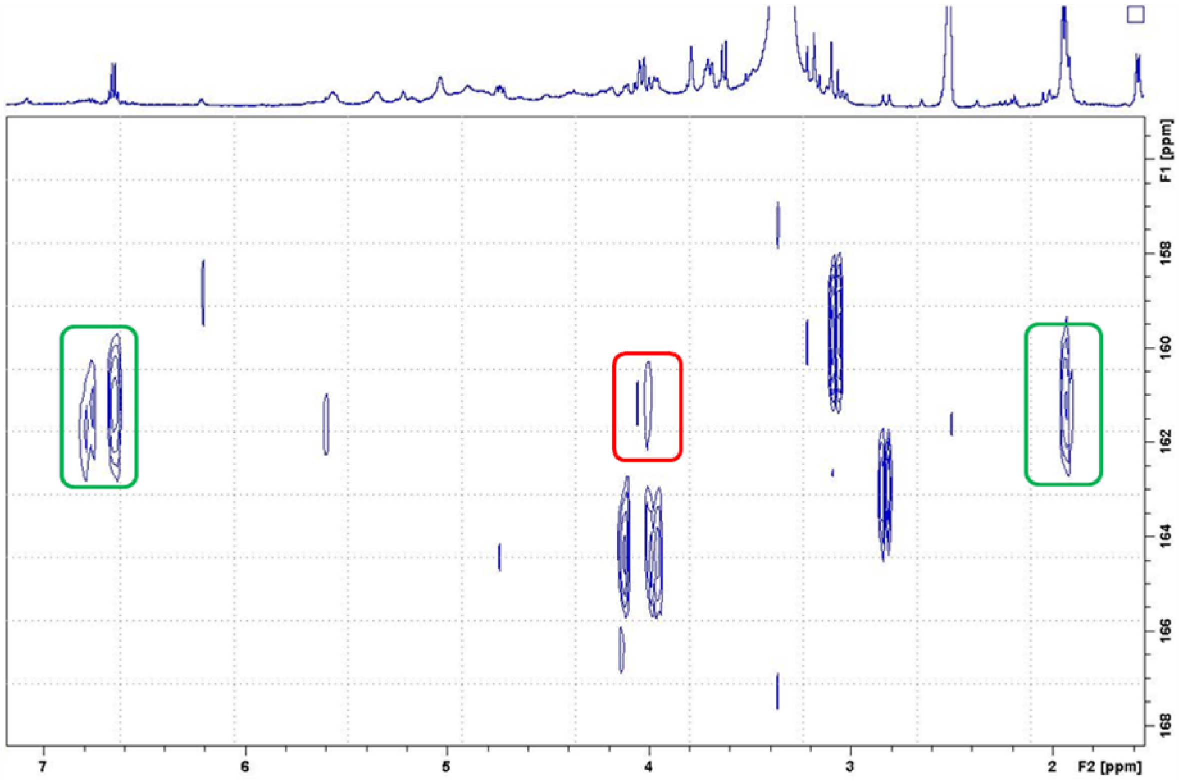

Figure S14. Structure of 6-hydroxyl-13-*O*-paulyl-paulinone (6').

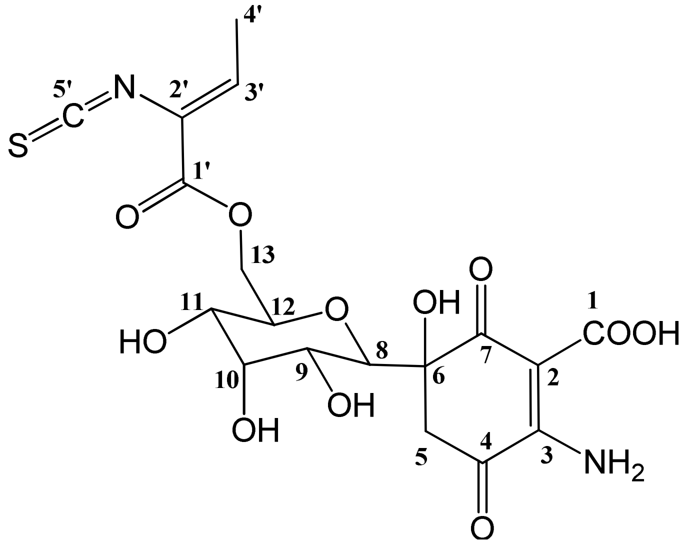

Table S2. 6-hydroxyl-13-*O*-paulyl-paulinone (6') ^{13}C and ^{1}H NMR data acquired in DMSO-d_6 (500 MHz, 24 °C).

Position	$\delta^{13}C$ (ppm)	$\delta^{1}H$ (ppm)	*J* (Hz)
1	n. d.	-	-
2	n. d.	-	-
3	159.7	-	-
4	188.9	-	-
5	48.6	3.19, d<br>3.08, d	16.0<br>16.0
6	78.1	-	-
7	198.5	-	-
8	77.2	3.62, d	10.0
9	70.0	3.28, dd	10.0, 2.9
10	71.2	3.78, br. s	-
11	66.3	3.36, br. dd	-
12	73.6	3.69, dt	10.2, 2.8
13	65.1	4.05, dd<br>4.00, dd	11.3, 3.0<br>11.3, 1.4
1'	161.2	-	-
2'	123.1	-	-
3'	136.5	6.64, q	6.9
4'	14.9	1.93, d	6.9
5'	n. d.	-	-

$\delta^{13}C$ were determined from HSQC and HMBC spectra.

Structural characterization of (2E)-17-(4'-aminophenyl)-3,11,15-trihydroxy-10,12,14-trimethyl-17-oxo-heptadeca-4,6,8-trienoic acid (7)

The HRMS data of compound **7** confirmed a molecular formula of $C_{26}H_{37}NO_6$ that was deduced from the pseudo molecular ion at *m/z* 460.2699 $[M+H]^+$ obtained by ESI-TOF analysis (Figure S15). Analysis of correlations observed by ^{1}H NMR spectrum acquired in DMSO-d_6 (Figures S16 and S17). The molecular formula points to 9 double bond equivalents (DBE). Protons observed in the ^{1}H NMR spectrum (31) (Figures S16 and S17) points compound **7** should contain six interchangeable protons. In addition, the ^{1}H NMR spectra shows clearly 10 sp^2 protons, four of them in a *para*-disubstituted benzenic ring, and three methyl groups in resonance as a doublet. Chemical shifts observed in COSY, HSQC and HMBC spectra (Table S3) confirmed the correlations and connectivity of the molecule and the stereochemistry of three *trans* conjugated

double bonds. Location of the –NH_2 group in para position of the aromatic ring was based in the chemical shift of the quaternary carbon carrying it (δ ^{13}C, 153.6 ppm) and the MS/MS data showing a fragmented molecular ion at *m/z* 120.0450 $[M+H]^+$ corresponding to a *p*-aminoacetophenonic acid (Figure S18).

Several *p*-aminoacetophenonic acids have been previously described [4]. The chemical shifts of the *p*-aminoacetophenonic acid structurally closest to **7**, (2E)-11-(4′-aminophenyl)-5,9-dihydroxy-4,6,8-trimethyl-11-oxo-undec-2-enoic acid [5], were compared to those of **7** showing an excellent concordance. This allowed identification of compound **7** as (2E)-17-(4’-aminophenyl)-3,11,15-trihydroxy-10,12,14-trimethyl-17-oxo-heptadeca-4,6,8-trienoic acid (Figure S19).

Figure S15. (+) ESI-TOF of (2E)-17-(4’-aminophenyl)-3,11,15-trihydroxy-10,12,14-trimethyl-17-oxo-heptadeca-4,6,8-trienoic acid (7).

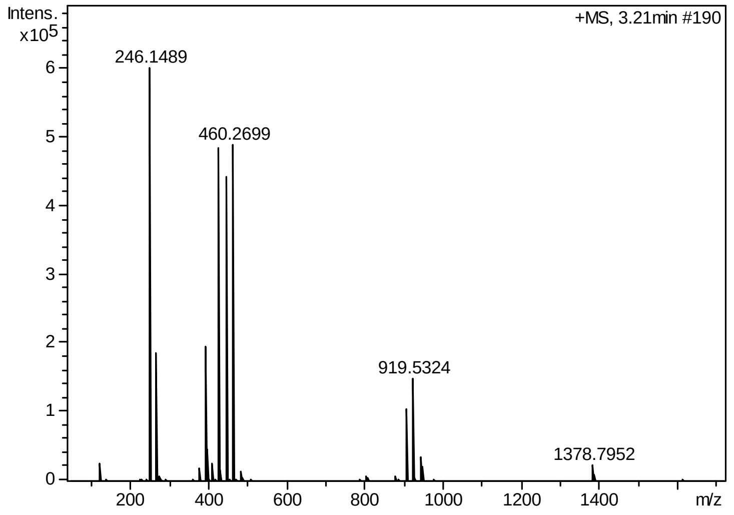

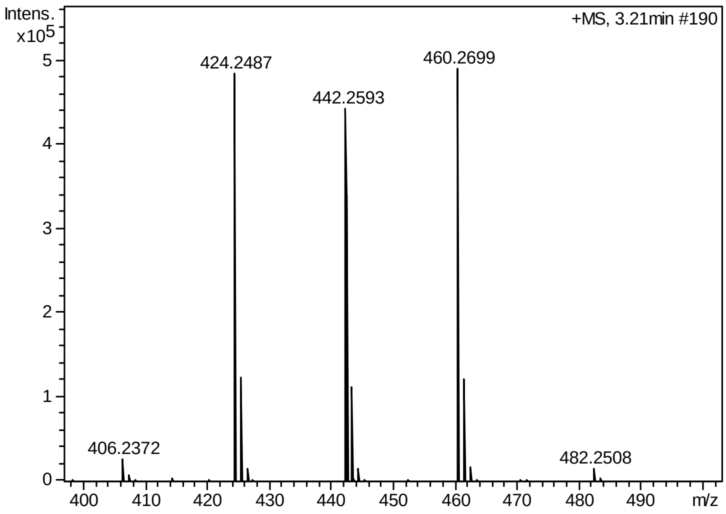

Figure S16. ^{1}**H NMR spectrum of (2E)-17-(4'-aminophenyl)-3,11,15-trihydroxy-10,12,14-trimethyl-17-oxo-heptadeca-4,6,8-trienoic acid (7) (DMSO-d_6, 500 MHz, 24 °C).**

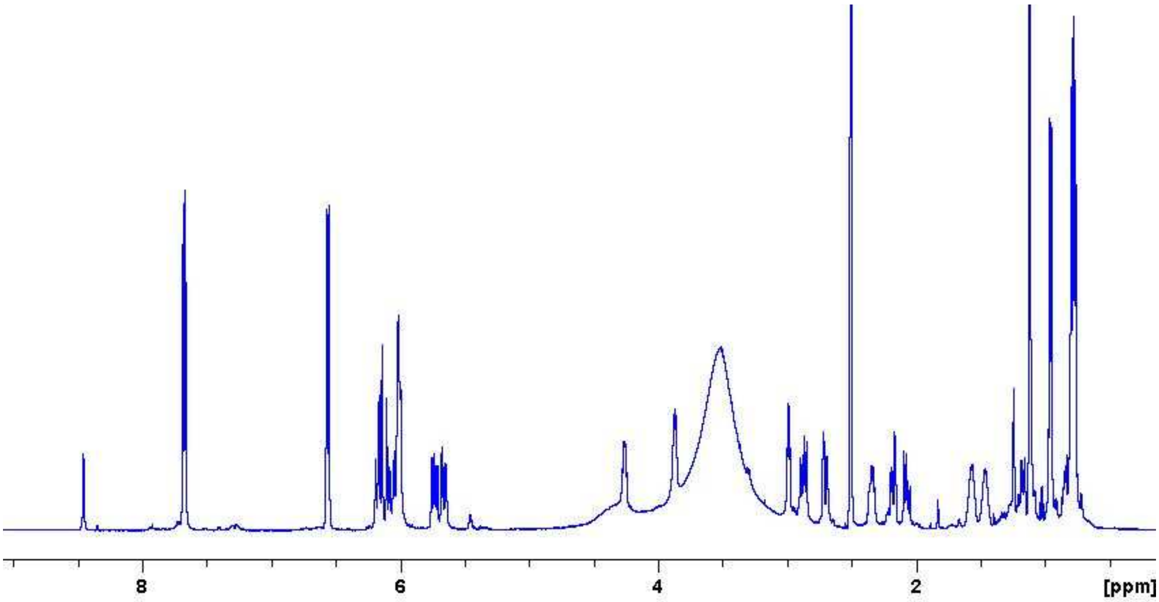

Figure S17. ^{1}H NMR spectrum of (2E)-17-(4'-aminophenyl)-3,11,15-trihydroxy-10,12,14-trimethyl-17-oxo-heptadeca-4,6,8-trienoic acid (7) (DMSO-d_6, 500 MHz, 24 °C). Data edited using a T2 filter (in red) and a diffusion filter (in blue).

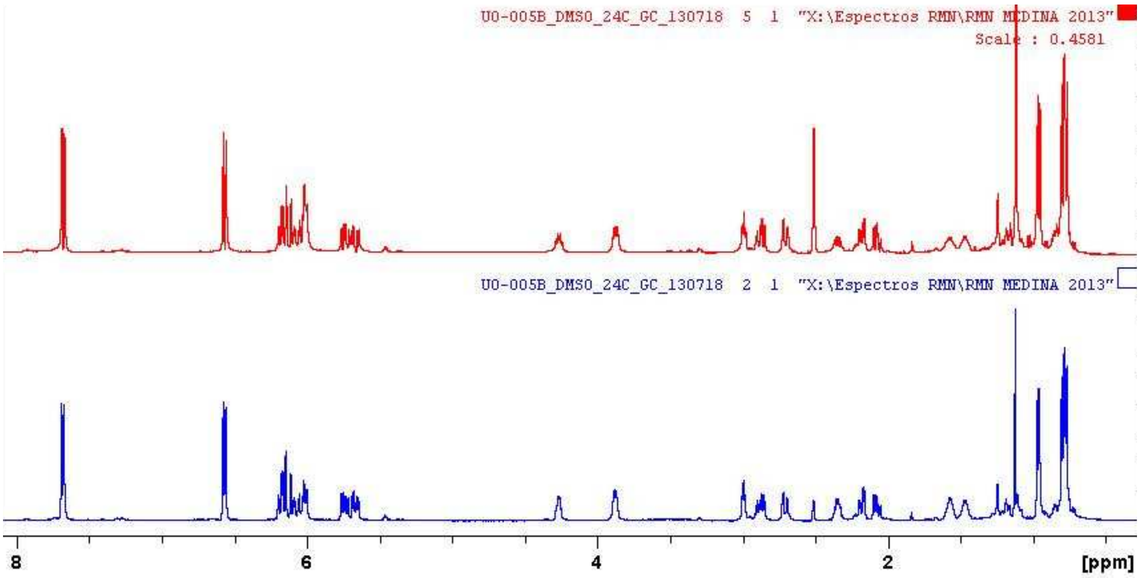

Figure S18. Structure of fragmented molecular ion at *m/z* 120.0450 [*M*+H]$^{+}$.

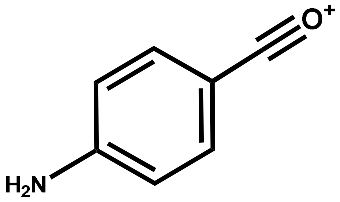

Figure S19. Structure of (2E)-17-(4'-aminophenyl)-3,11,15-trihydroxy-10,12,14-trimethyl-17-oxo-heptadeca-4,6,8-trienoic acid (7).

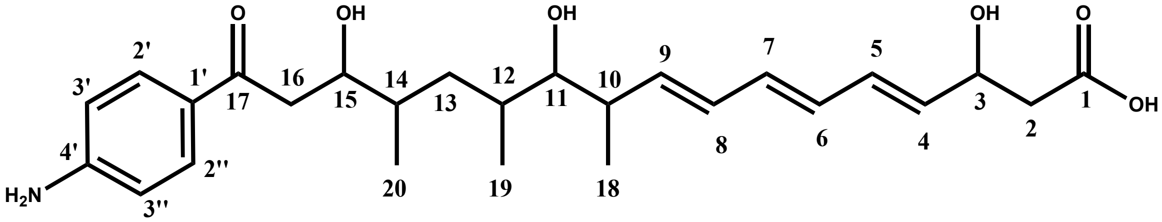

Table S3. (2E)-17-(4'-aminophenyl)-3,11,15-trihydroxy-10,12,14-trimethyl-17-oxo-heptadeca-4,6,8-trienoic acid (7) ^{13}C and ^{1}H NMR data acquired in DMSO-d_6 (500 MHz, 24 °C).

Position	δ ^{13}C (ppm)	δ ^{1}H (ppm)	*J* (Hz)
1	173.8	-	-
2	43.6	2.17, dd<br>2.07, dd	15.1, 4.5<br>15.1, 8.4
3	68.5	4.26, ddd	8.4, 5.7, 4.5
4	137.2	5.66, dd	15.1, 5.7
5	128.6	6.16, dd	15.2, 9.9
6	130.5	6.08, dd	15.1, 9.8
7	132.8	6.16, dd	15.2, 9.9
8	130.2	6.02, dd	ca. 15, ca. 10
9	138.0	5.74, dd	15.2, 8.5
10	40.1	2.34, app. sextet	ca. 7
11	78.4	2.99, dd	5.5, 5.5
12	33.6	1.46, m	n. d.
13	35.9	1.18, m<br>1.11, m	n. d.
14	36.2	1.57, m	n. d.
15	72.1	3.87, ddd	8.6, 4.3, 3.6
16	41.6	2.87, dd<br>2.70, dd	15.0, 8.6<br>15.0, 3.6
17	197.1	-	-
1'	125.4	-	-
2' 2''	130.9	7.67, d	8.7
3' 3''	112.8	6.56, d	8.7
4'	153.6	-	-

δ^{13}C were determined from HSQC and HMBC spectra.

Structural characterization of 2-aminobenzoic acid (anthranilic acid) (8)

The HRMS data of compound **8** confirmed a molecular formula of $C_7H_7NO_2$ based on the observed ions at *m/z* 138.0550 [M+H]$^+$ obtained by ESI-TOF analysis (Figure S20). Searching the molecular formula in the Dictionary of Natural Products [6] retrieved 20 possible candidates. NMR analysis was then pursued in order to identify if any of those candidates corresponded to the target compound **8**. The proton spectrum (Figures S21 and S22) clearly showed the aromatic nature of the molecule. The integration of signals accounted for a total of 4 aromatic protons and the multiplicity pattern observed corresponded to an *ortho*-disubstituted benzene ring. This information was entered as

query alongside the molecular formula in the Dictionary of Natural Products NMR feature database. Only one compound, 2-aminobenzoic acid (anthranilic acid) (Figure S23), was obtained and its structure perfectly matches the observed NMR spectrum. Likewise the absorption maxima reported in literature for this compound also match those observed in the UV-vis DAD spectrum showed previously. Further 2D NMR experiments were acquired to fully assign its ^{1}H and ^{13}C resonances.

Figure S20. (+) ESI-TOF spectrum of 2-aminobenzoic acid (8).

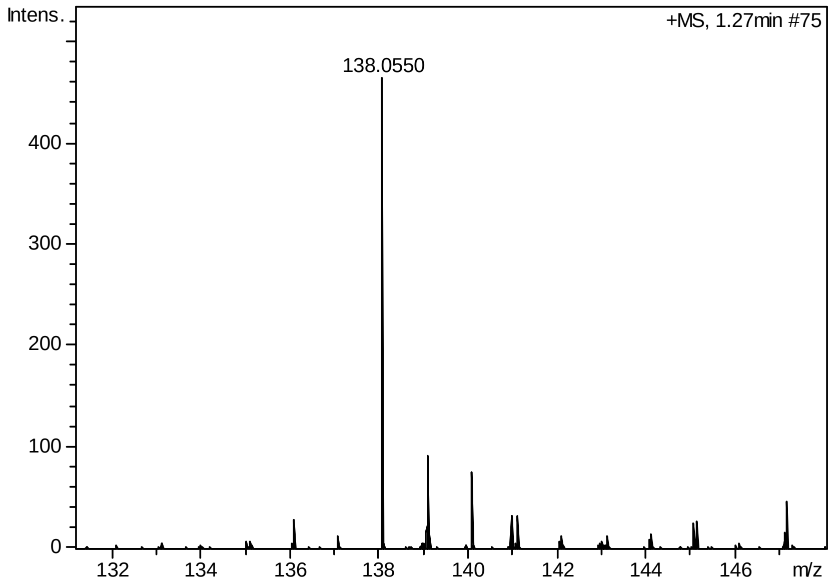

Figure S21. ^{1}H NMR spectrum of 2-aminobenzoic acid (8) (CD_3OD, 500 MHz, 24 °C).

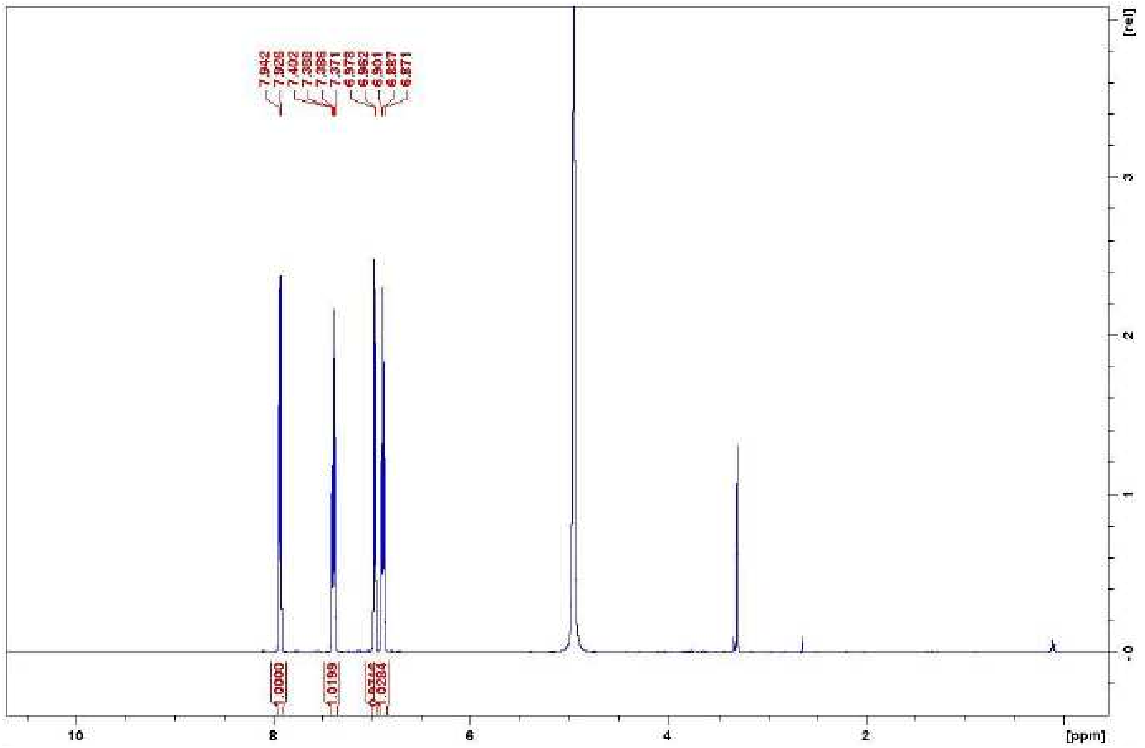

Figure S22. Detail of ^{1}H NMR spectrum of 2-aminobenzoic acid (8) (CD_3OD, 500 MHz, 24 °C).

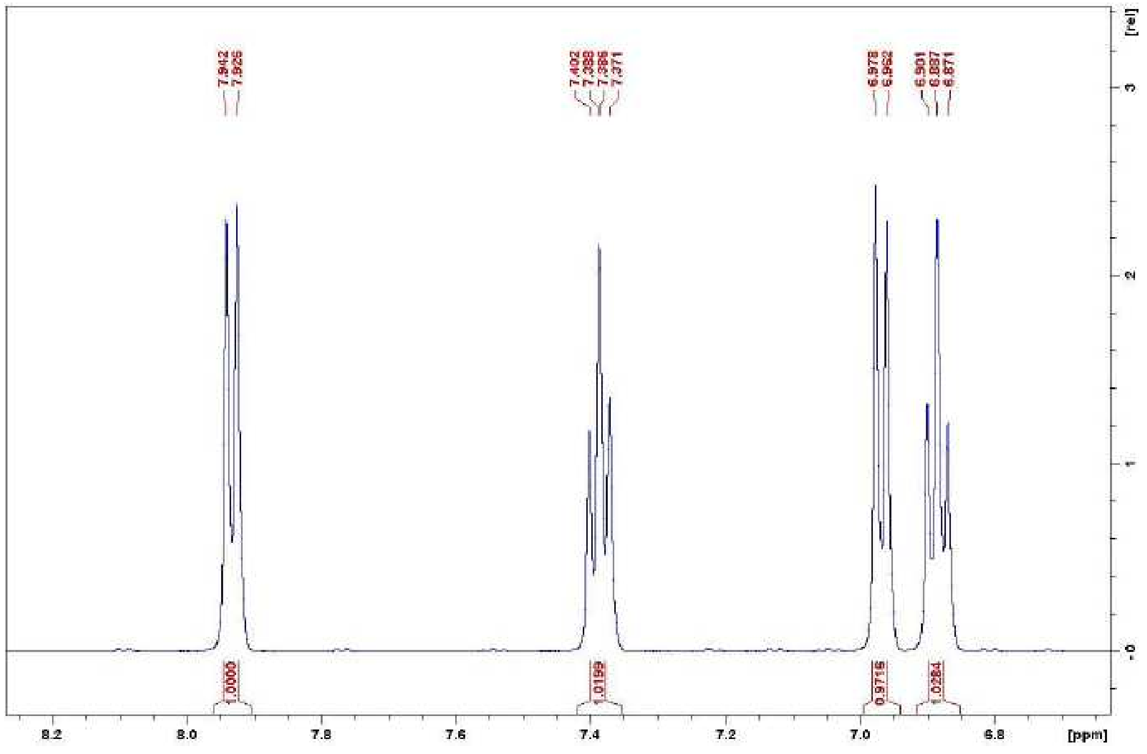

Figure S23. Structure of 2-aminobenzoic acid (8).

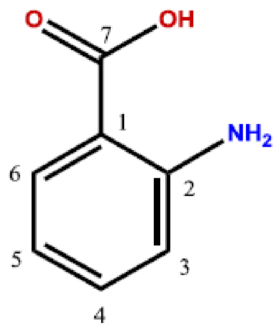

Table S4. 2-aminobenzoic acid (8) ^{13}C and ^{1}H NMR data acquired in CD_3OD (500 MHz, 24 °C).

Position	δ ^{13}C (ppm)	δ ^{1}H (ppm)	*J* (Hz)
1	113.5	-	-
2	144.7	-	-
3	117.9	6.97, d	8.3
4	132.9	7.39, t	7.6
5	118.5	6.89, t	7.8
6	130.6	7.93, d	8.0
7	168.3	-	-

δ^{13}C were determined from HSQC and HMBC spectra.

Structural characterization of *N*-acetyl-*orto*-aminobenzoic acid (10)

The HRMS data of compound **10** confirmed a molecular formula of $C_9H_9NO_3$ that was deduced from the pseudo molecular ion at *m/z* 180.0654 $[M+H]^+$ obtained by ESI-TOF analysis (Figure S24). Analysis of correlations observed by ^{1}H NMR spectrum acquired in CD_3OD showed the presence of a single methyl group and four sp^2 protons (Figure S25). The molecular formula points to 6 double bond equivalents (DBE). On the other hand, protons observed in the ^{1}H NMR spectrum points two of them are interchangeable. Chemical shifts observed in COSY and HSQC spectra suggest an *orto*-disubstituted benzenic ring (4 DBE), which points the 2 DBE remaining might correspond to carbonyl groups. These data might indicate the two interchangeable protons are as –OH and –NH– groups.

Dereplication of compound **10** using the Chapman & Hall Dictionary of Natural Products database [6] allowed its identification as *N*-acetyl-*orto*-aminobenzoic acid (Figure S26).

Figure S24. (+) ESI-TOF spectrum of *N*-acetyl-*orto*-aminobenzoic acid (10).

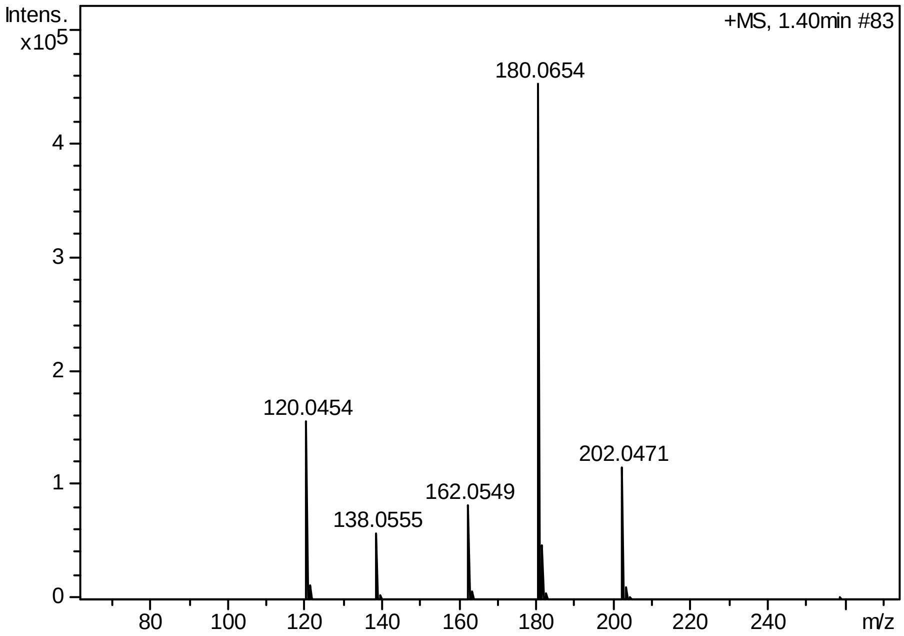

Figure S25. ^{1}H NMR spectrum of *N*-acetyl-*orto*-aminobenzoic acid (10) (CD_3OD, 500 MHz, 24 °C).

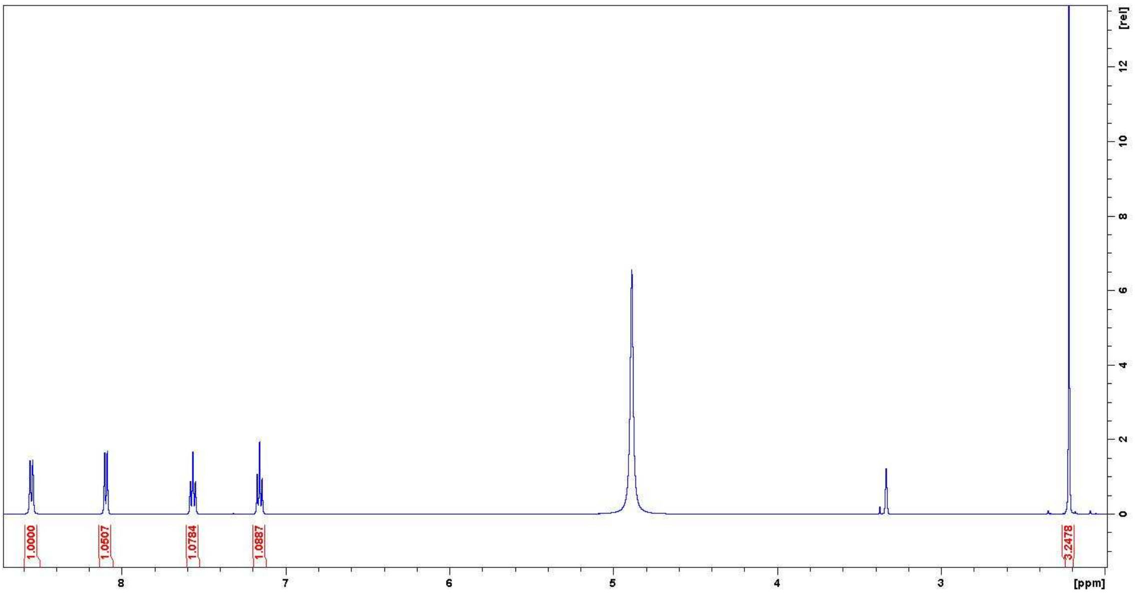

Figure S26. Structure of *N*-acetyl-*orto*-aminobenzoic acid (10).

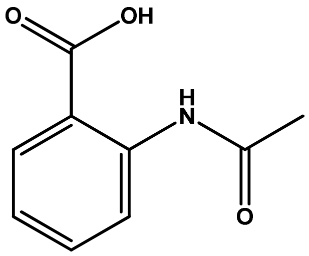

Structural characterization of deoxydehydrochorismic acid (11)

The HRMS data of compound **11** confirmed a molecular formula of $C_{10}H_8O_5$ that was deduced from the pseudo molecular ion at *m/z* 231.0256 $[M+H]^+$ obtained by ESI-TOF analysis (Figure S27). Analysis of correlations observed by ^1H NMR spectrum acquired in CD_3OD showed the presence of six sp^2 protons (Figure S28). The molecular formula points to 7 double bond equivalents (DBE). Protons observed in the ^1H NMR spectrum (6) points two of them are interchangeable. Chemical shifts observed in HSQC spectrum show the presence of a vinyl group; consequently four of the sp^2 protons are aromatic. Furthermore, COSY spectrum suggests a *meta*-disubstituted benzenic ring. Five DBE correspond to the vinyl group and the aromatic ring, then the two additional DBE correspond to carbonyl groups pointing that the interchangeable protons are as an –OH group.

Dereplication of compound **11** using the Chapman & Hall Dictionary of Natural Products database [6] allowed its identification as deoxydehydrochorismic acid (Figure S29).

Figure S27. (+) ESI-TOF spectrum of deoxydehydrochorismic acid (11).

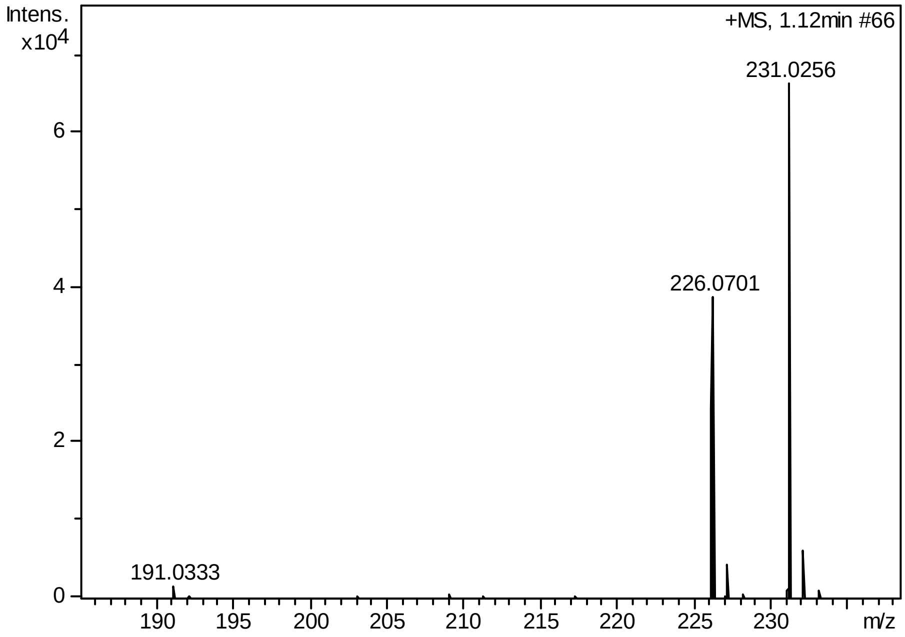

Figure S28. ^{1}H NMR spectrum of deoxydehydrochorismic acid (11) (CD_3OD, 500 MHz, 24 °C).

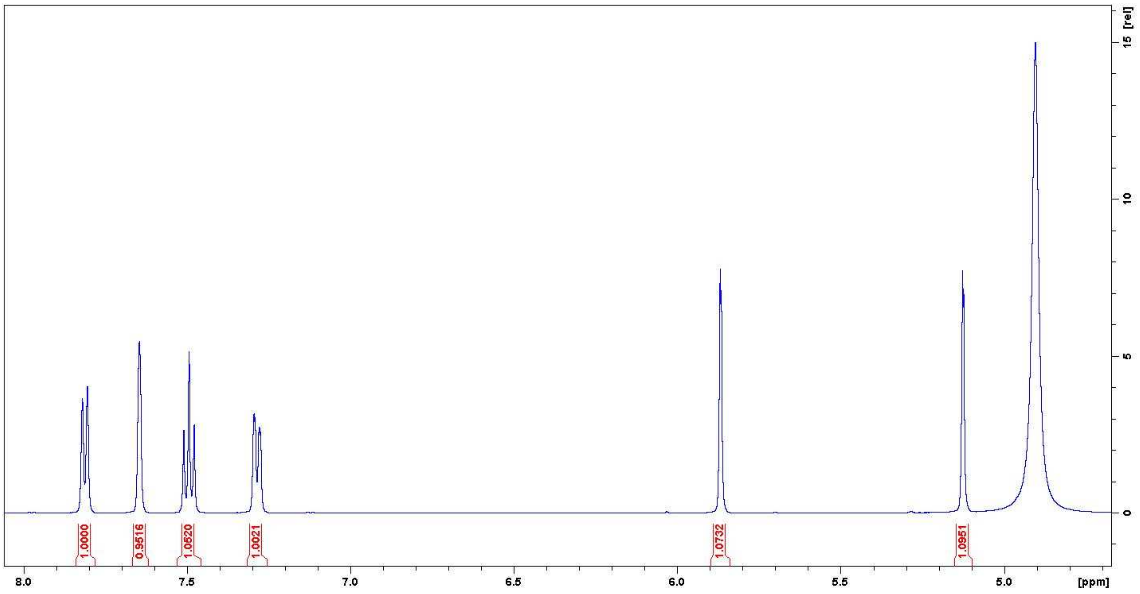

Figure S29. Structure of deoxydehydrochorismic acid (11).

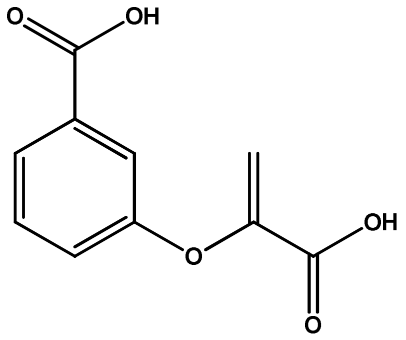

Structural characterization of paulomycin F (12)

The HRMS data of compound **12** confirmed a molecular formula of $C_{29}H_{38}N_2O_{16}S$ based on the observed pseudo molecular ion at *m/z* 725.1842 $[M+Na]^+$ obtained by ESI-TOF analysis (Figure S30). The molecular formula of this compound contains a sulfur atom suggesting a paulomycin-like structure and matches that reported for paulomycin F and its epimer senfolomycin B [3]. The ^1H and the HSQC NMR spectra (Figures S31 to S35) confirmed that **12** possesses a paulomycin-like structure. Furthermore, many of the observed signals showed strong resemblance to those displayed by 6-hydroxyl-13-*O*-paulyl-paulinone (**6'**) and the presence of a paulic acid moiety was confirmed. Detailed analysis of the proton and HSQC spectra indicated that the main component very likely displayed the connectivity described for paulomycin F and senfolomycin B. However, unambiguous establishment of the structure required the acquisition of additional 2D NMR spectra including, COSY, TOCSY, NOESY and HMBC (Figure S36). Analysis of such spectral set confirmed the main component in compound **12** corresponds to paulomycin F (Figure S37, Table S5). The epimer at 3', senfolomycin B, was discarded based on coupling constants displayed by the proton at position 3' and comparison with the reported NMR data [3,7].

Figure S30. (+) ESI-TOF spectrum of paulomycin F (12).

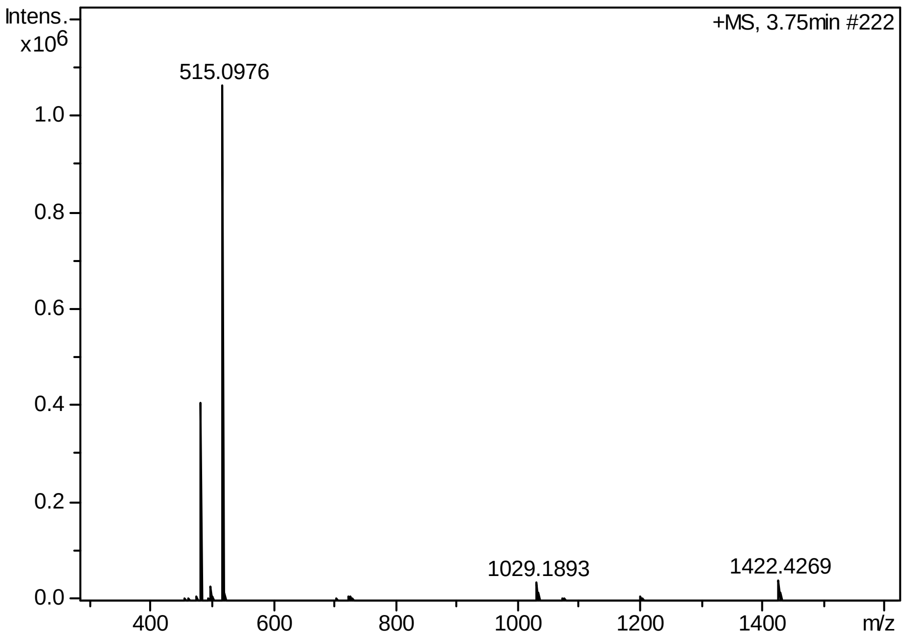

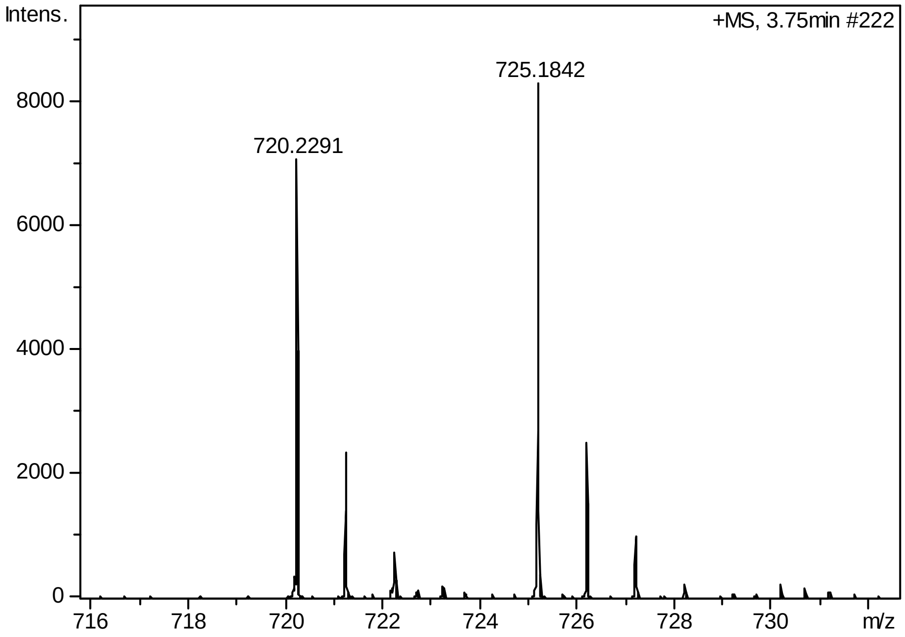

Figure S31. ¹H NMR spectrum of paulomycin F (12) (DMSO-d_6, 500 MHz, 24 °C).

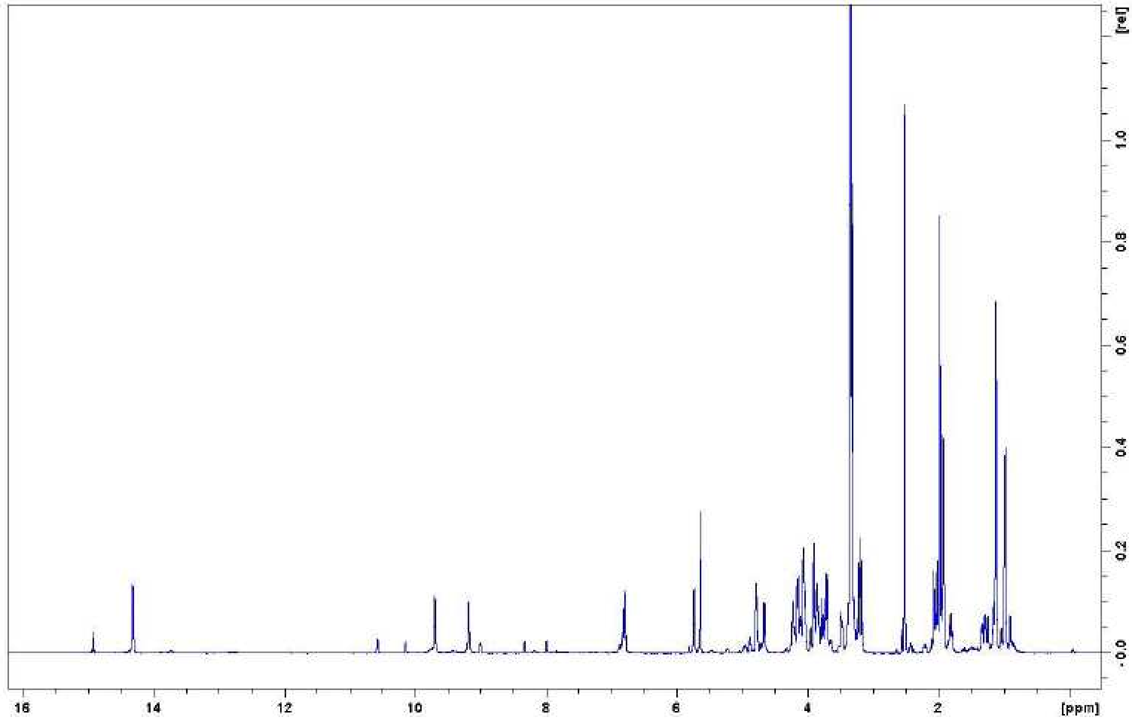

Figure S32. Expansion of ¹H NMR spectrum of paulomycin F (12) (DMSO-d_6, 500 MHz, 24 °C).

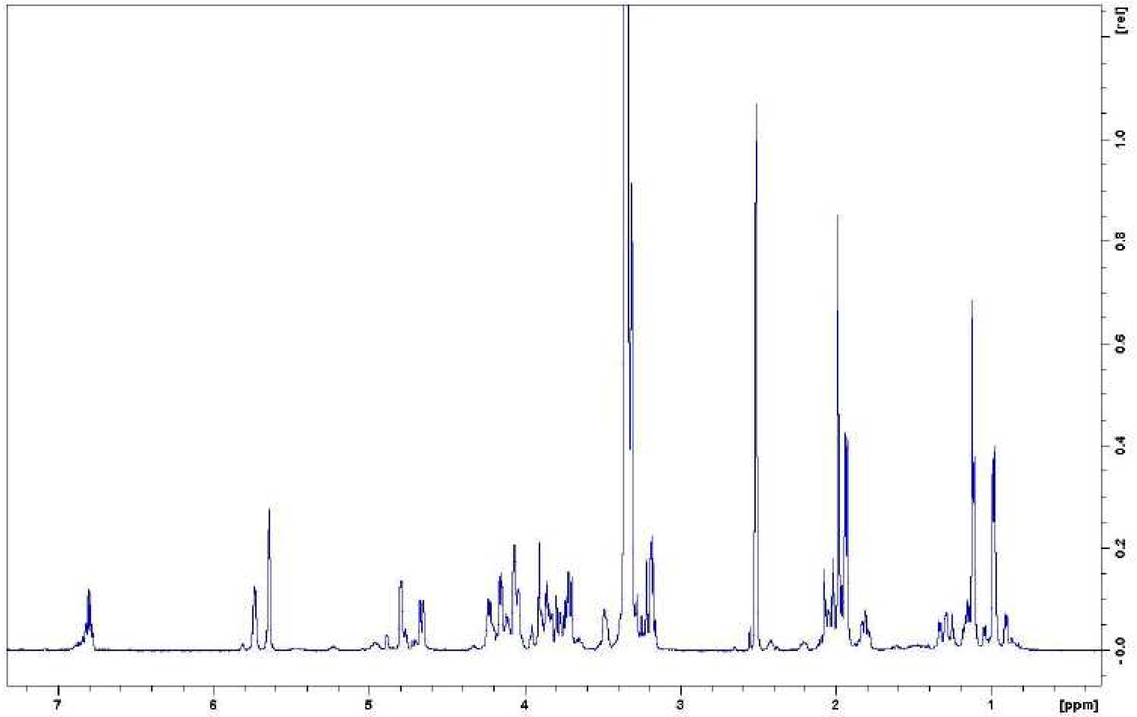

Figure S33. Expansion of ^{1}H NMR spectrum of paulomycin F (12) (DMSO-d_6, 500 MHz, 24 °C).

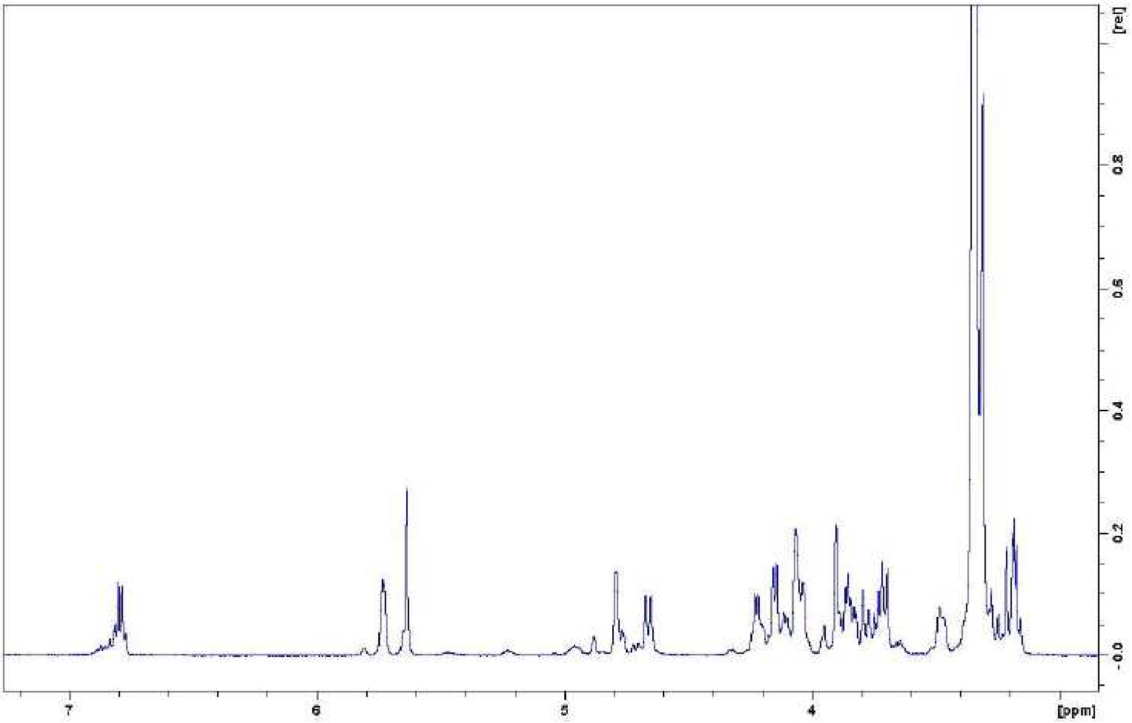

Figure S34. Expansion of ^{1}H NMR spectrum of paulomycin F (12) (DMSO-d_6, 500 MHz, 24 °C).

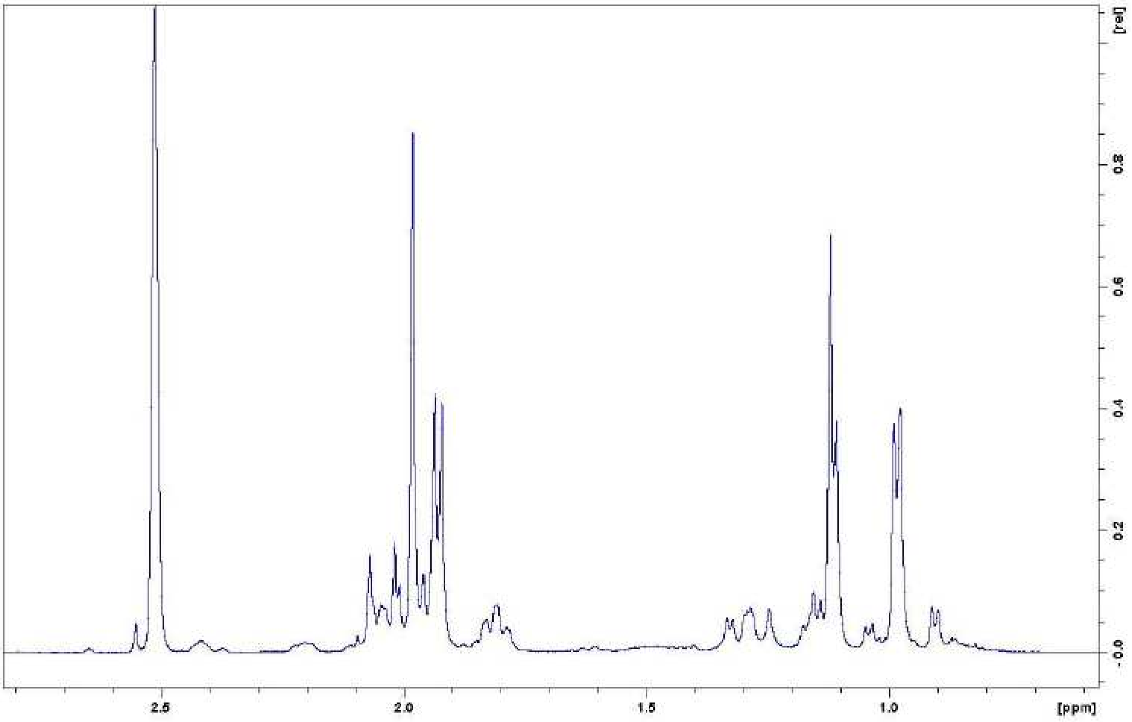

Figure S35. ^{1}H-^{13}C HSQC spectrum of paulomycin F (12) (DMSO-d_6, 500 MHz, 24 °C).

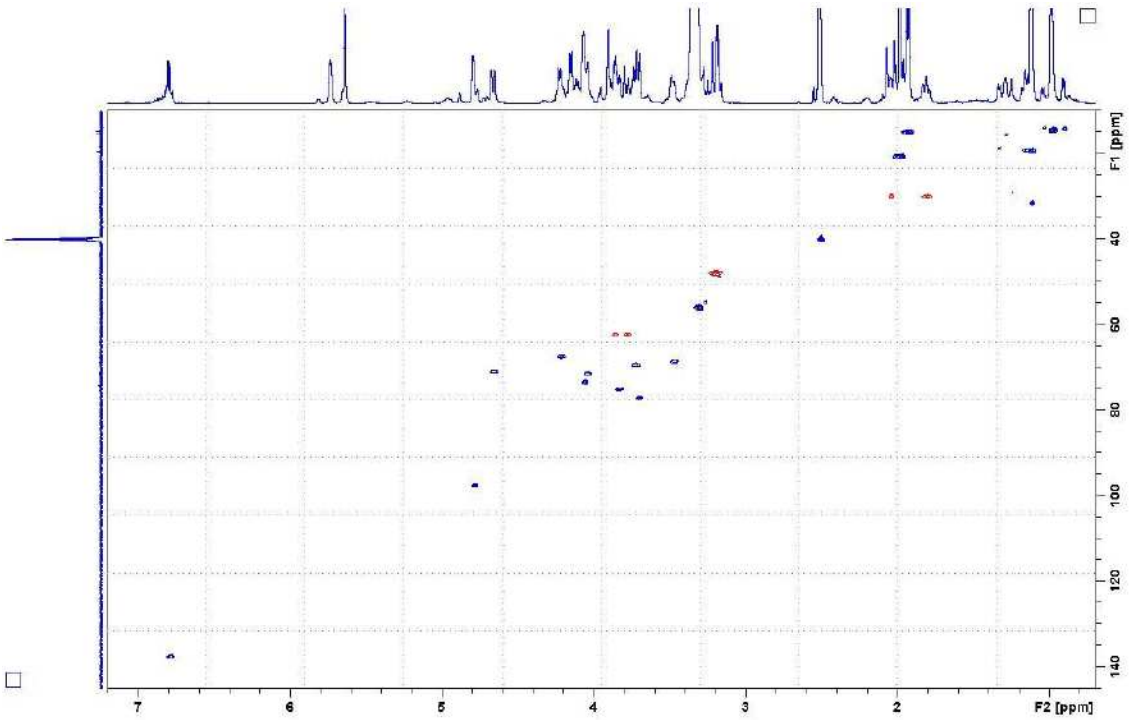

Figure S36. Expansion of the ^{1}H-^{13}C HMBC spectrum of paulomycin F (12) (DMSO-d_6, 500 MHz, 24 °C). The key correlation between carbon 1'' and proton at position 11 is highlighted in red color. The other HMBC correlations involving this carbonyl with the protons at positions 3'' and 4'' are highlighted in green color.

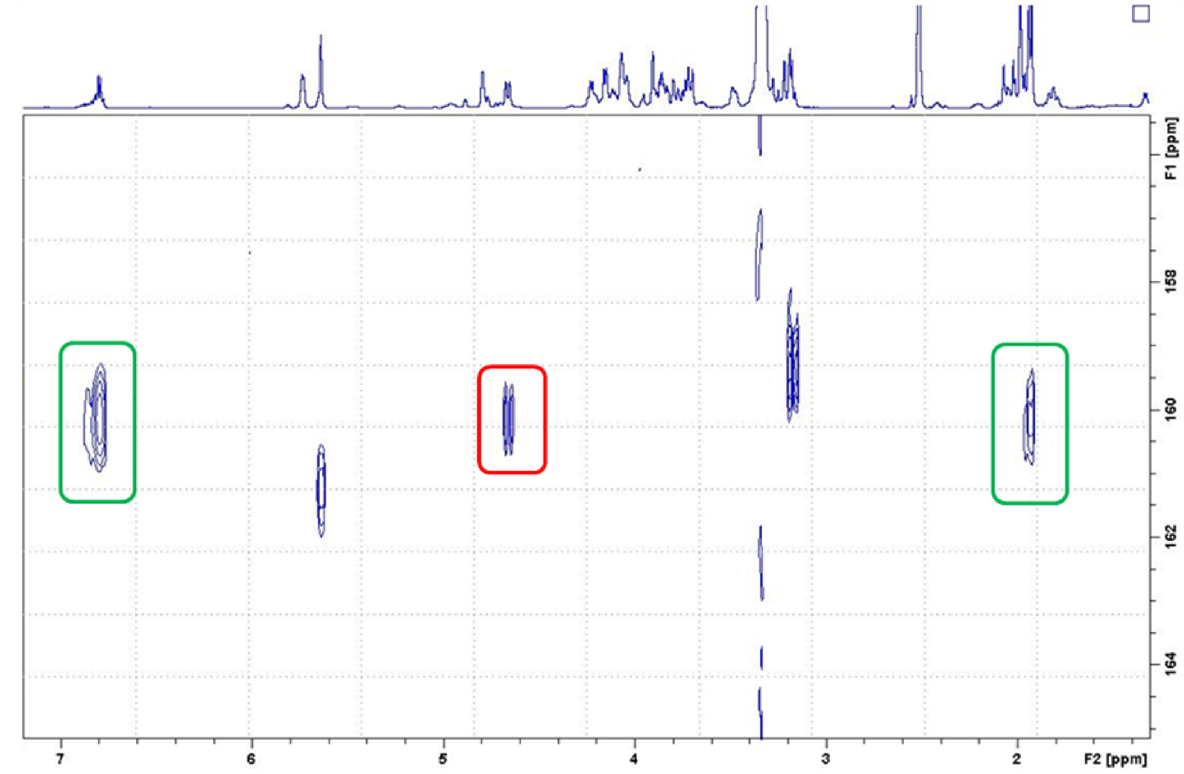

Figure S37. Structure of paulomycin F (12). Numbering of the molecule is according to [3] and absolute stereochemistry is according to [8]).

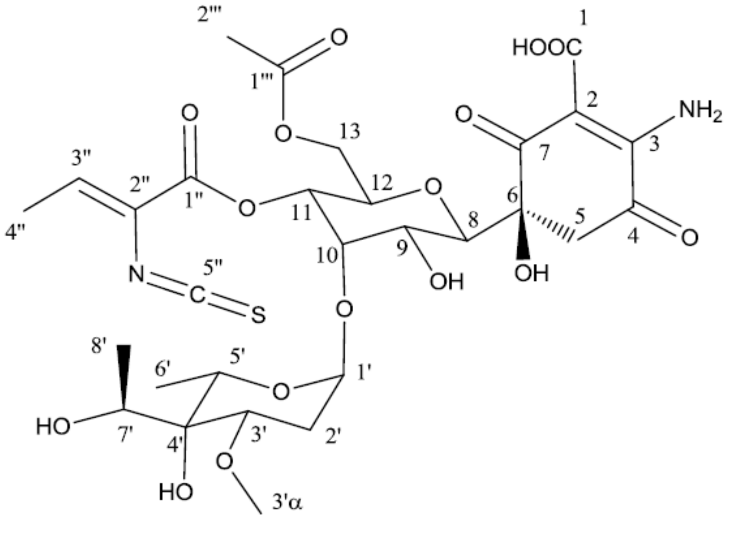

Table S5. Paulomycin F (12) ^{13}C and ^{1}H NMR data (δ in ppm) acquired in DMSO-*d6* (500 MHz, 24 °C).

Position	δ ^{13}C	δ ^{1}H (mult, *J* in Hz)	Position	δ ^{13}C	δ ^{1}H (mult, *J* in Hz)
1	n.d.	-	1’	97.7	4.79 (br s)
2	99.3	-	2’	30.2	2.04 (m)<br>1.81 (td, 11.5, 3.0)
3	159.3	-	3’	75.2	3.84 (dd, 10.9, 4.4)
4	189.0	-	3’α	56.0	3.30 (s)
5	48.0	3.20 (m)	4’	74.2	-
6	77.8	-	5’	67.6	4.22 (quart., 6.5)
7	198.0	-	6’	14.6	0.98 (d, 6.0)
8	77.3	3.71 (d, 10.0)	7’	69.5	3.73 (m)
9	68.7	3.48 (br d, 9.1)	8’	19.5	1.11 (d, 6.1)
10	73.6	4.07 (m)	1’’	160.2	-
11	71.1	4.66 (br d, 10.0)	2’’	122.6	-
12	71.5	4.04 (m)	3’’	137.7	6.79 (quart., 7.2)
13	62.5	3.86 (m)<br>3.78 (br d, 11.7)	4’’	15.1	1.93 (d, 7.0)
			5’’	n.d.	-
			1’’’	170.4	-
			2’’’	20.8	1.98 (s)

δ^{13}C were determined from HSQC and HMBC spectra.

Structural characterization of 13-*O*-deacetyl-13-*O*-paulyl-paulomycin E (14’)

The HRMS data of compound **14’** confirmed a molecular formula $C_{27}H_{34}N_2O_{15}S$ based on the observed pseudo molecular ion at *m/z* 659.1751 $[M+H]^+$ (Figure S38). The base peak in the mass spectrum does not correspond to the pseudo molecular ion but to a fragment at *m/z* 473.0864 $[M+H]^+$ (Figure S38). Such a behavior is typical for paulomycins (at least in our equipment and under the ionization conditions employed) as it was already found for paulomycin F (**12**). This observed in-source fragmentation is due to the easy breakage of the glycosidic bond of paulomycins. Interestingly the mentioned *m/z* = 473.0864 $[M+H]^+$ fragment displays identical accurate mass as the pseudo molecular ion observed for 6-hydroxyl-13-*O*-paulyl-paulinone (**6’**) suggesting they could in fact share the same molecular structure.

To establish the connectivity and confirm the structure of **14’** the ^{1}H NMR spectrum and a set of 2D NMR spectra (including COSY, HSQC and HMBC) were acquired (Figures S39 to S44). Many of the observed signals showed strong resemblance to those displayed by 6-hydroxyl-13-*O*-paulyl-paulinone (**6’**) and paulomycin F (**12**). Analysis of such spectral set confirmed that the main fragment observed in **14’** mass spectrum corresponds to 13-*O*-deacetyl-13-*O*-paulyl-paulomycin E (Figure S45, Table S6).

The epimer at C3’ (senfolomycin-like configuration) was discarded based on the coupling constants displayed by the proton in position 3’ and by comparison with the NMR data of paulomycin F (**12**). The paulic acid unit is acylating the primary hydroxyl as it was found for 6-hydroxyl-13-*O*-paulyl-paulinone (**6’**). Chemical shifts provide further confirmation of the acylation position. The deshielding of the methylene protons at C-13 with respect to paulomycin F (**12**) is in perfect agreement with the acylation of the primary hydroxyl. In a similar manner, the shielding of the methine proton at C-11

with respect to paulomycin F (**12**) indicates no acylation on that position. The same behavior was already observed for 6-hydroxyl-13-*O*-paulyl-paulinone (**6'**) and in fact, both 6-hydroxyl-13-*O*-paulyl-paulinone (**6'**) and compound **14'** display very close proton and carbon chemical shifts at positions C-11, C-12 and C-13. On the other hand, the paulomycose unit with a methyl ketone substituent at position 4' matches that reported for paulomycin E [7].

Figure S38. (+) ESI-TOF spectrum of 13-*O*-deacetyl-13-*O*-paulyl-paulomycin E (14').

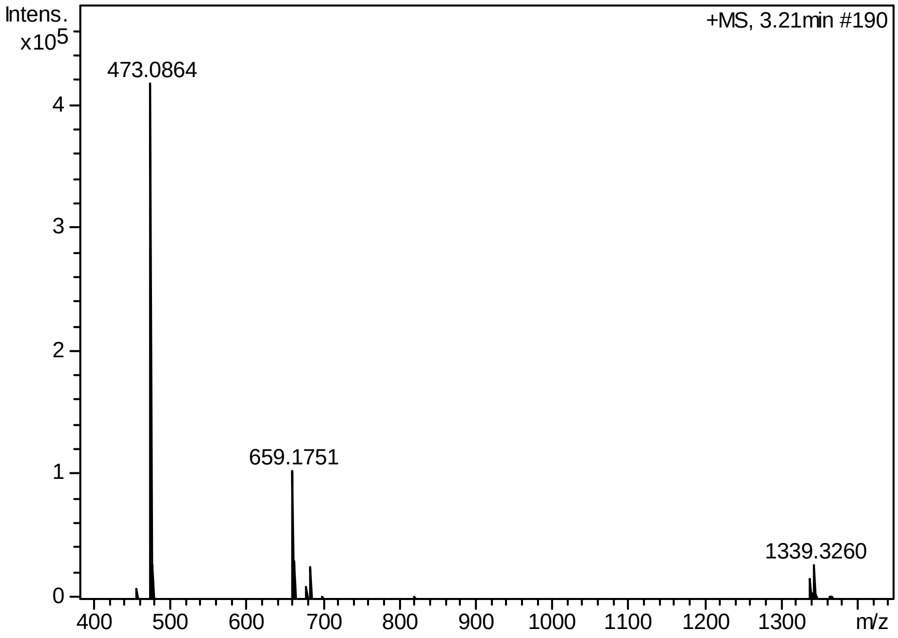

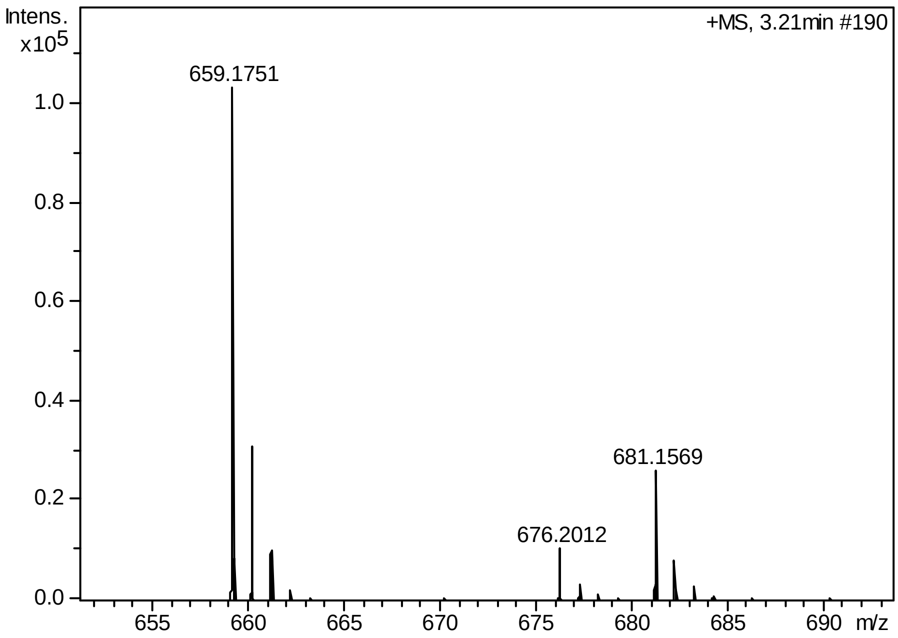
Intens.
x10^5
+MS, 3.21min #190
659.1751
681.1569
676.2012
1.0
0.8
0.6
0.4
0.2
0.0
655
660
665
670
675
680
685
690
m/z

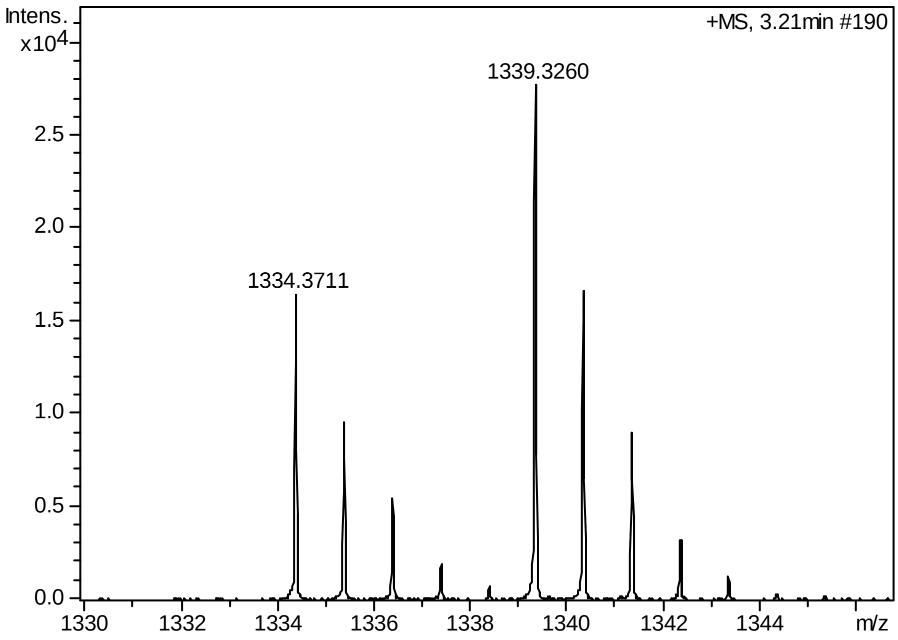
Intens.
x10^4
+MS, 3.21min #190
1339.3260
1334.3711
2.5
2.0
1.5
1.0
0.5
0.0
1330
1332
1334
1336
1338
1340
1342
1344
m/z

Figure S39. ^{1}**H NMR spectrum of 13-*O*-deacetyl-13-*O*-paulyl-paulomycin E (14’) (DMSO-d_6, 500 MHz, 24 °C).**

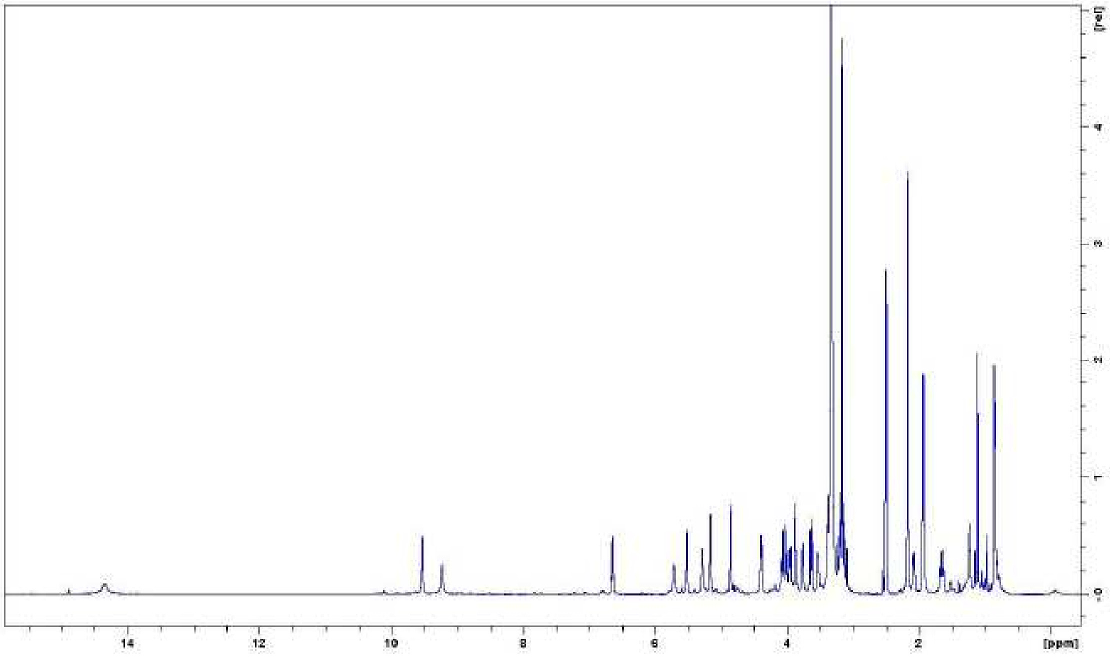

Figure S40. **Expansion of** ^{1}**H NMR spectrum of 13-*O*-deacetyl-13-*O*-paulyl-paulomycin E (14’) (DMSO-d_6, 500 MHz, 24 °C).**

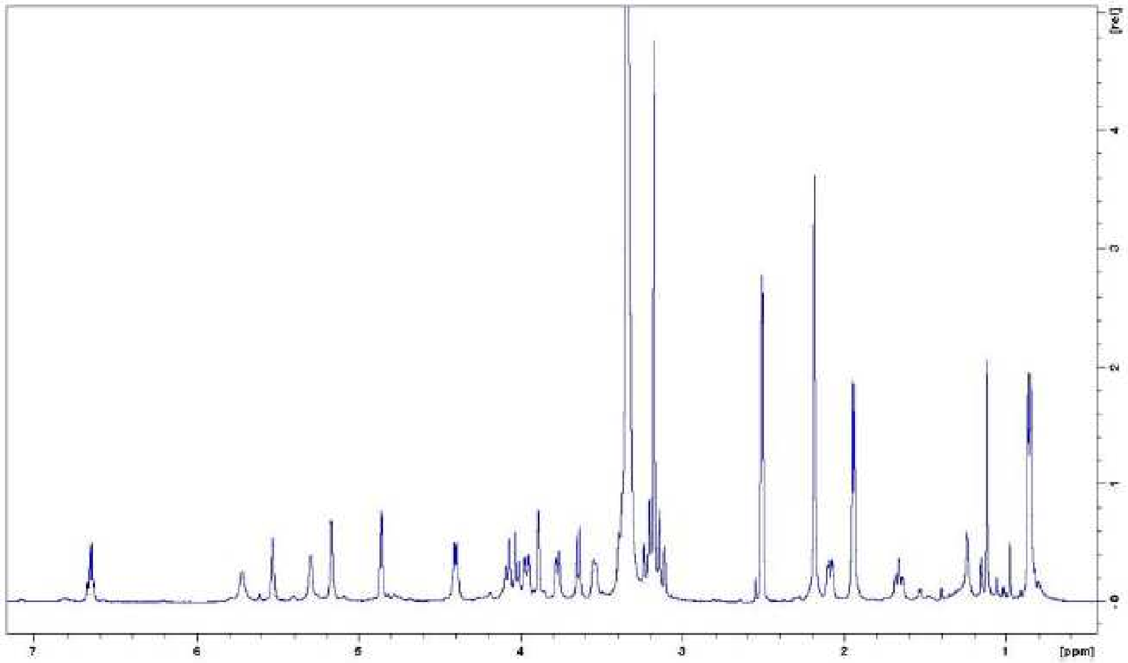

Figure S41. **Expansion of ^{1}H NMR spectrum of 13-*O*-deacetyl-13-*O*-paulyl-paulomycin E (14') (DMSO-*d*$_6$, 500 MHz, 24 °C).**

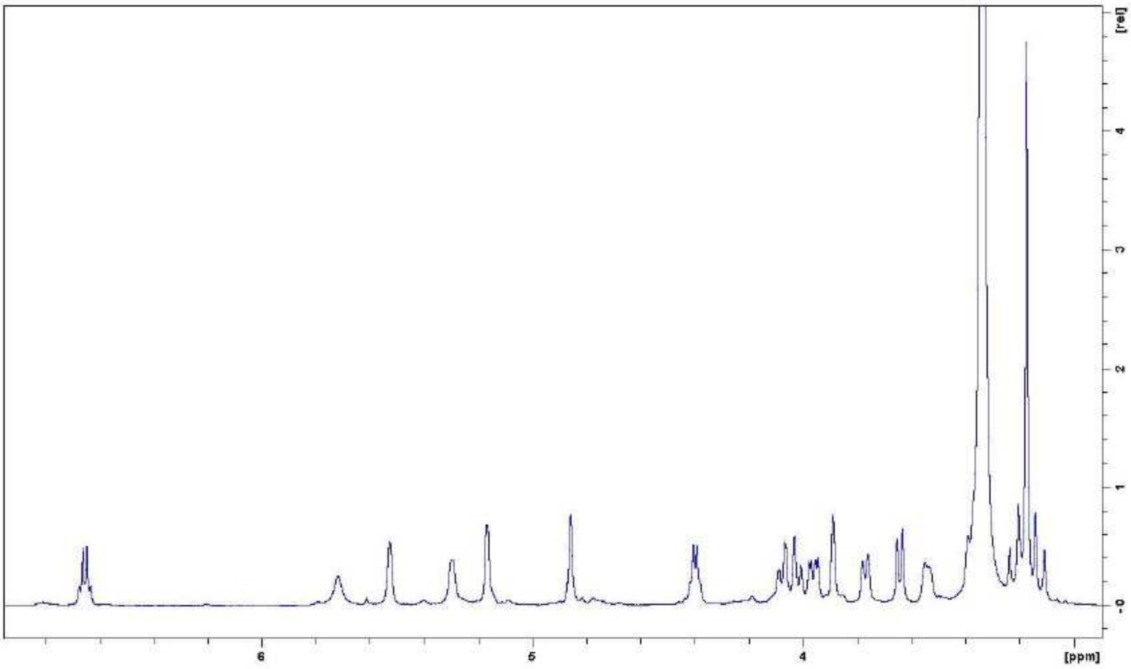

Figure S42. **Expansion of ^{1}H NMR spectrum of 13-*O*-deacetyl-13-*O*-paulyl-paulomycin E (14') (DMSO-*d*$_6$, 500 MHz, 24 °C).**

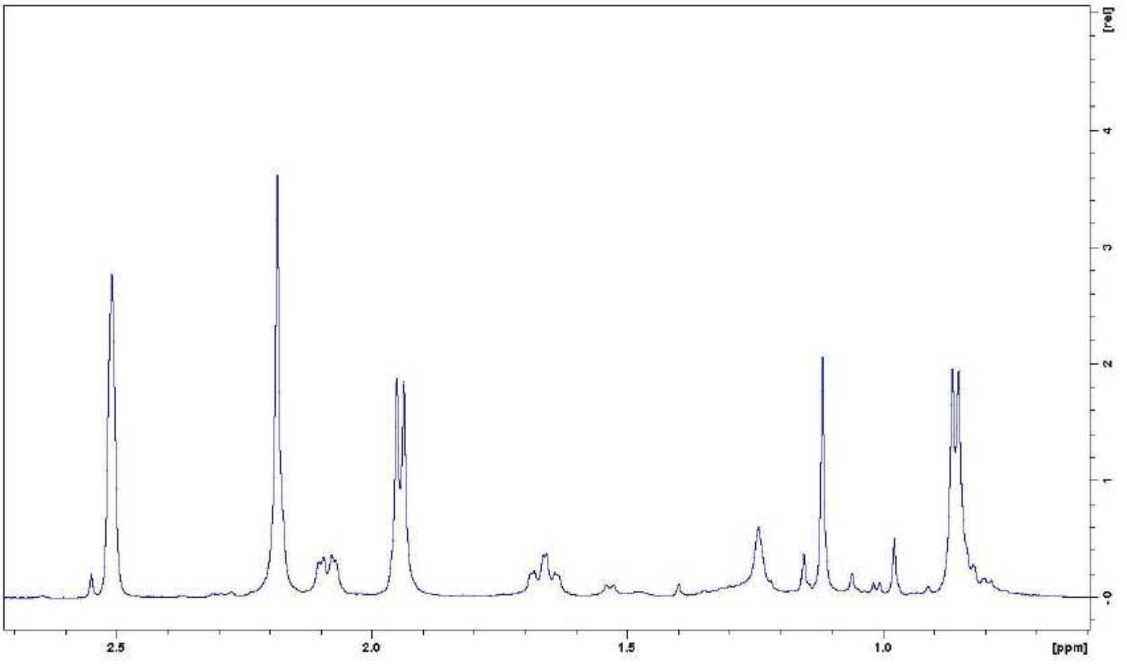

Figure S43. **^{1}H-^{13}C HSQC spectrum of 13-*O*-deacetyl-13-*O*-paulyl-paulomycin E (14') (DMSO-d_6, 500 MHz, 24 °C).**

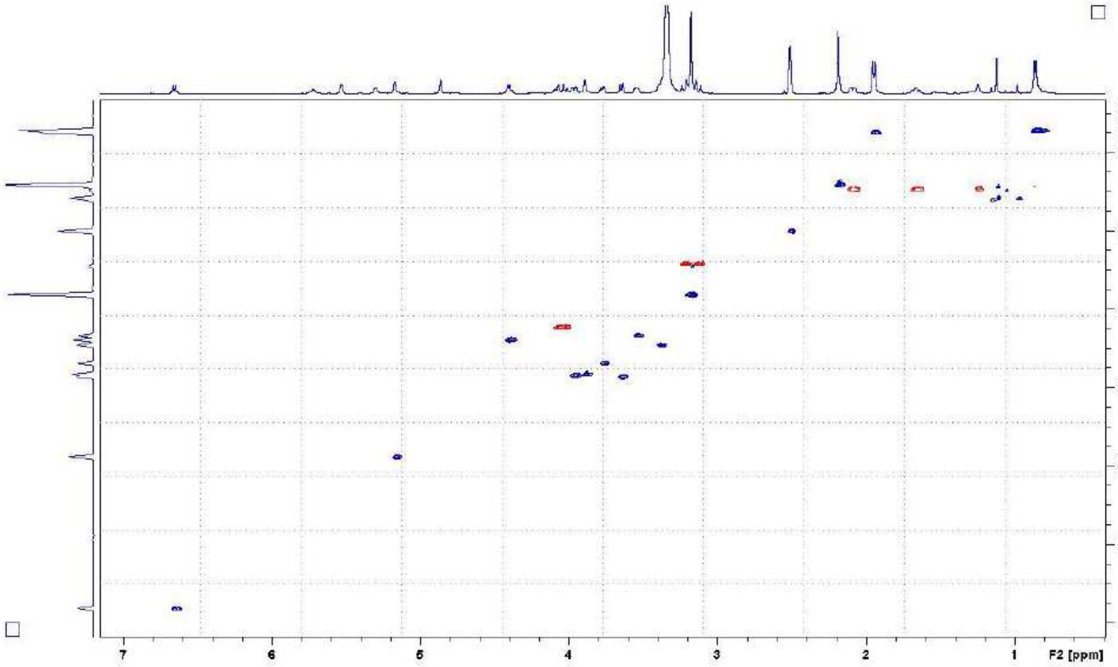

Figure S44. **Expansion of the ^{1}H-^{13}C HMBC spectrum of 13-*O*-deacetyl-13-*O*-paulyl-paulomycin E (14') (DMSO-d_6, 500 MHz, 24 °C).** The key correlation between carbon 1'' and protons at position 13 is highlighted in red color. The other HMBC correlations involving this carbonyl with the protons at positions 3'' and 4'' are highlighted in green color. Better S/N is observed in the HMBC spectrum of **15'** for comparison.

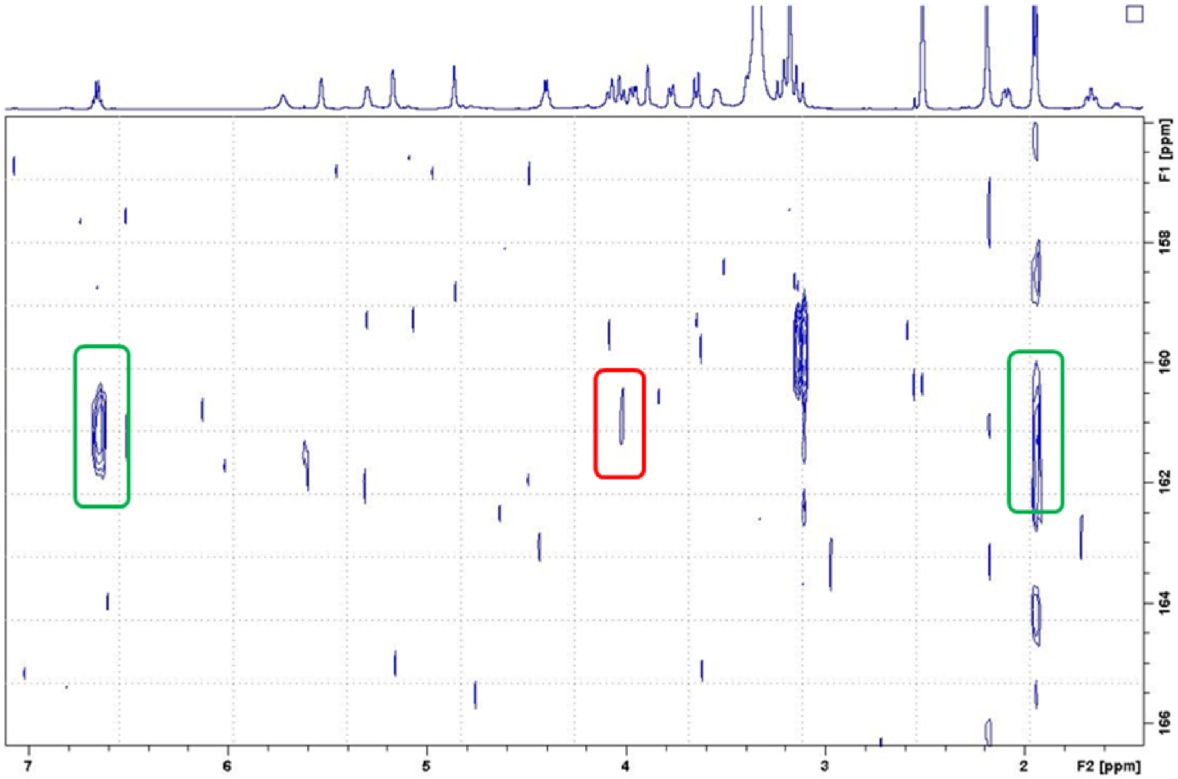

Figure S45. **Structure of 13-*O*-deacetyl-13-*O*-paulyl-paulomycin E (14').**

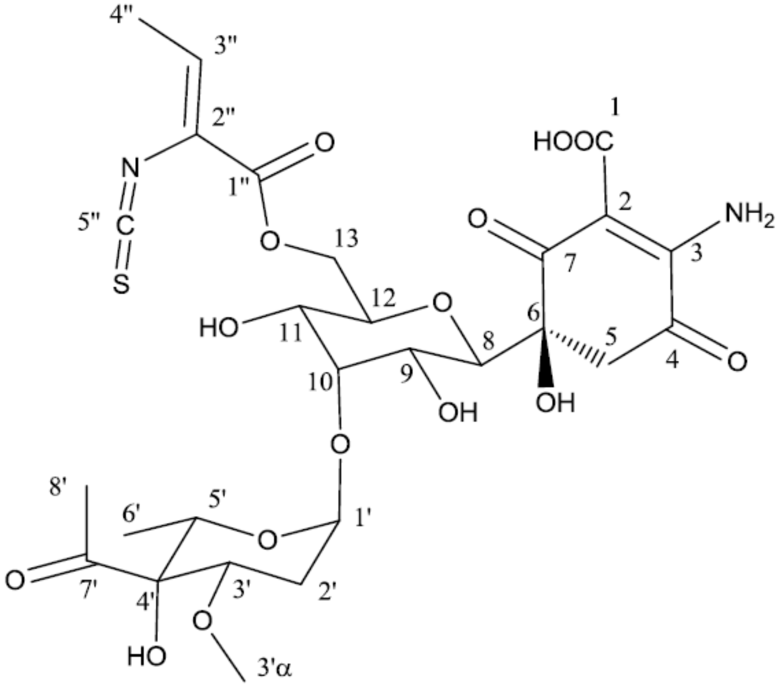

Table S6. 13-*O*-deacetyl-13-*O*-paulyl-paulomycin E (14') ^{13}C and ^{1}H NMR data (δ in ppm) acquired in DMSO-*d6* (500 MHz, 24 °C).

Position	δ ^{13}C	δ ^{1}H (mult, *J* in Hz)	Position	δ ^{13}C	δ ^{1}H (mult, *J* in Hz)
1	n.d.	-	1'	97.8	5.17 (d, 2.7)
2	99.5	-	2'	29.4	2.09 (dd, 11.9, 4.1)<br>1.66 (td, 12.0, 3.2)
3	159.8	-	3'	77.0	3.96 (dd, 11.9, 4.5)
4	189.1	-	3'α	56.2	3.17 (s)
5	48.4	3.22 (d, 16.1)<br>3.13 (d, 16.1)	4'	82.3	-
6	78.1	-	5'	67.9	4.40 (quart., 6.4)
7	198.5	-	6'	14.4	0.86 (d, 6.2)
8	77.3	3.64 (d, 9.9)	7'	213.7	-
9	69.1	3.38 (m, overlap)	8'	28.3	2.18 (s)
10	76.6	3.89 (br t)	1''	161.1	-
11	66.8	3.54 (br t, 8.0)	2''	123.1	-
12	73.8	3.77 (br dt, 9.8)	3''	136.4	6.66 (quart., 7.0)
13	64.7	4.08 (dd, 12.2, 2.5)<br>4.02 (br d, 11.9)	4''	15.0	1.94 (d, 7.0)
			5''	n.d.	-

δ^{13}C were determined from HSQC and HMBC spectra.

Structural characterization of 13-*O*-deacetyl-13-*O*-paulyl-paulomycin B (15')

The HRMS data of compound **15'** confirmed a molecular formula of $C_{31}H_{42}N_2O_{16}S$ based on the observed pseudo molecular ion at *m/z* 731.2325 $[M{+}H]^+$ (Figure S46). The base peak in the mass spectrum does not correspond to the pseudo molecular ion but to the same fragment at *m/z* = 473.0866 $[M{+}H]^+$ (Figure S46) already found for 13-*O*-deacetyl-13-*O*-paulyl-paulomycin E (**14'**). This suggested that 13-*O*-deacetyl-13-*O*-paulyl-paulomycin E (**14'**) and compound **15'** share the same molecular structure for that fragment.

To establish the connectivity and confirm the structure of **15'** the ^1H NMR spectrum and a set of 2D NMR spectra (including COSY, HSQC and HMBC) were acquired (Figures S47 to S52). As expected, many of the observed signals were almost identical to those observed for 13-*O*-deacetyl-13-*O*-paulyl-paulomycin E (**14'**). In depth interpretation of the correlations observed allowed establishing the structure of compound **15'** as 13-*O*-deacetyl-13-*O*-paulyl-paulomycin B (Figure S53, Table S7).

The epimer at 3' (senfolomycin-like configuration) was discarded based on the coupling constants displayed by the proton in position 3' and by comparison with the NMR data of paulomycin F (**12**) and 13-*O*-deacetyl-13-*O*-paulyl-paulomycin E (**14'**). As expected, the paulic acid unit is acylating the primary hydroxyl as it was found for 13-*O*-deacetyl-13-*O*-paulyl-paulomycin E (**14'**). On the other hand, the substituent at position 4' of the paulomycose unit matches that reported for paulomycin B [1,2]. The structure determined proves that the main fragment observed in the mass spectrum is identical to that found in 13-*O*-deacetyl-13-*O*-paulyl-paulomycin E (**14'**) and matches the structure determined for 6-hydroxyl-13-*O*-paulyl-paulinone (**6'**).

Figure S46. (+) ESI-TOF spectrum of 13-*O*-deacetyl-13-*O*-paulyl-paulomycin B (15’).

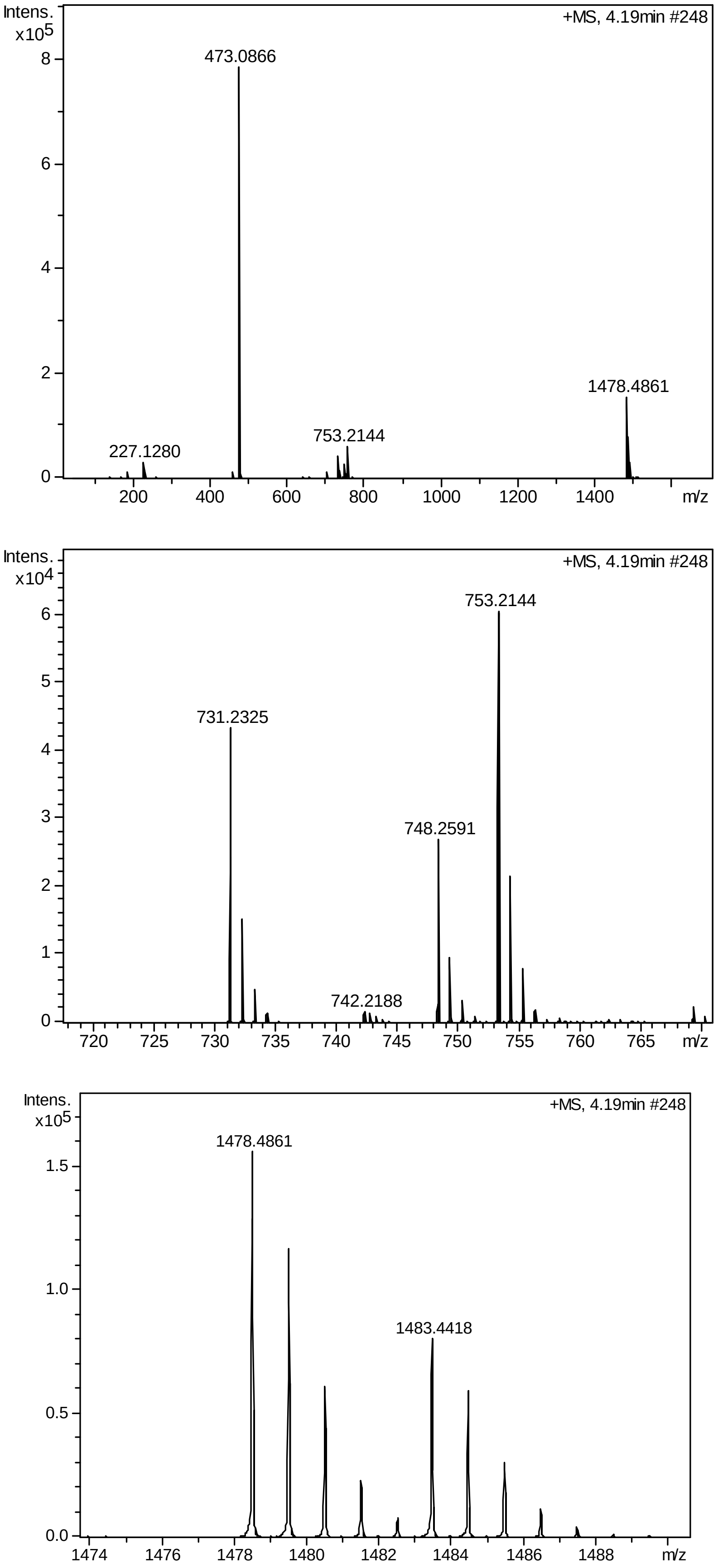

Figure S47. ^{1}H NMR spectrum of 13-*O*-deacetyl-13-*O*-paulyl-paulomycin B (15’) (DMSO-d_6, 500 MHz, 24 °C).

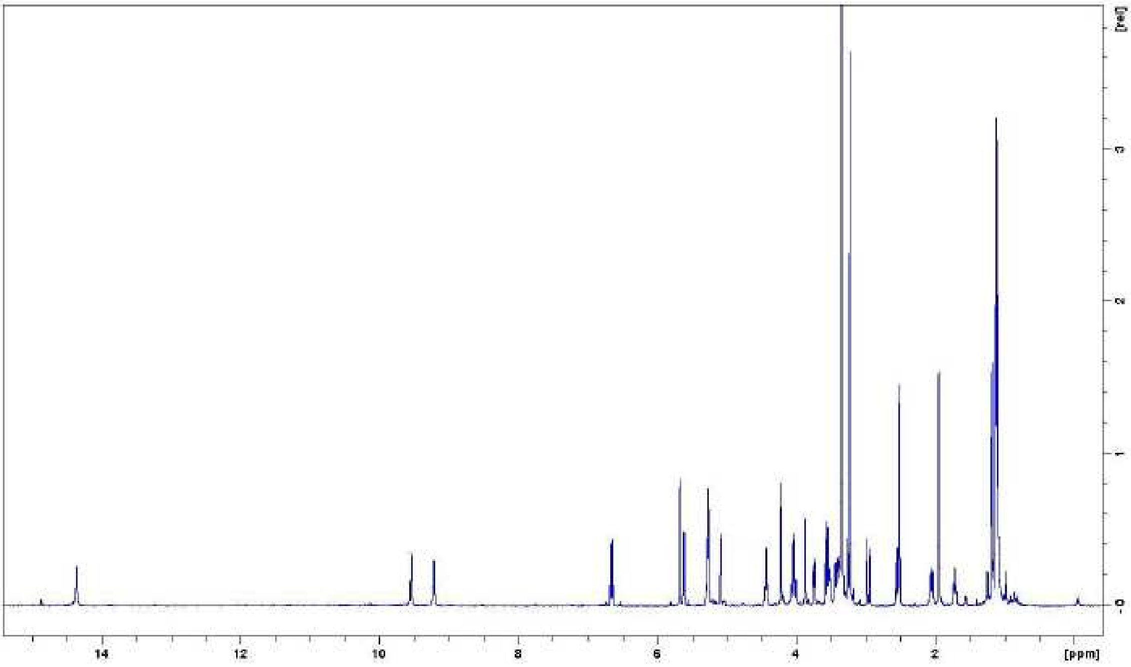

Figure S48. Expansion of ^{1}H NMR spectrum of 13-*O*-deacetyl-13-*O*-paulyl-paulomycin B (15’) (DMSO-d_6, 500 MHz, 24 °C).

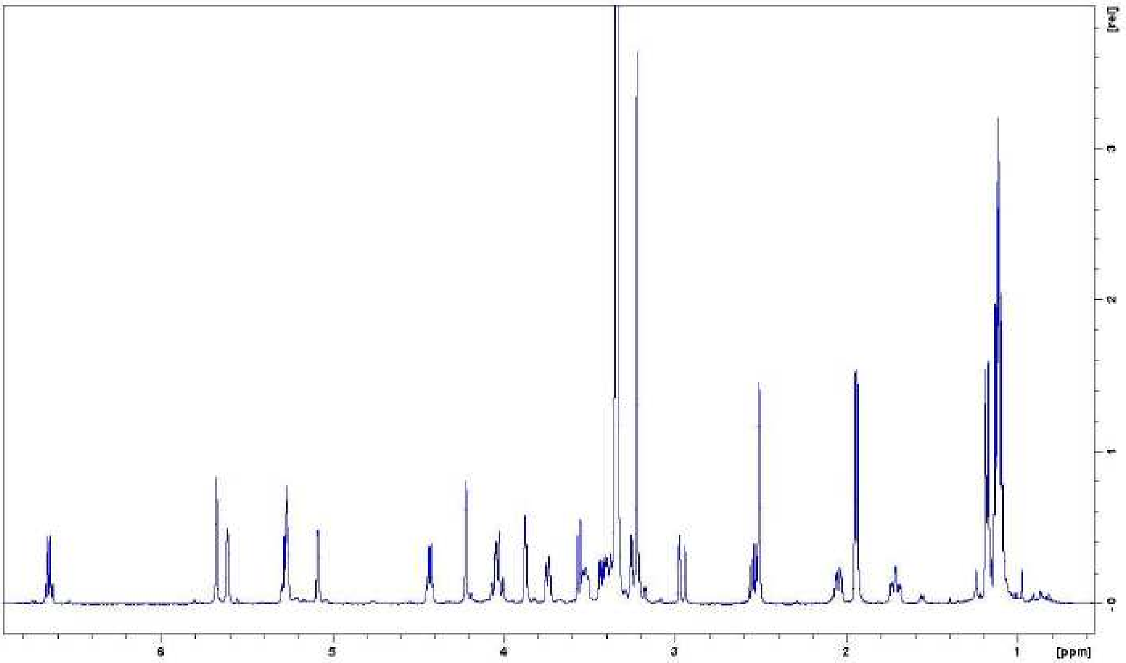

Figure S49. **Expansion of ^{1}H NMR spectrum of 13-*O*-deacetyl-13-*O*-paulyl-paulomycin B (15') (DMSO-d_6, 500 MHz, 24 °C).**

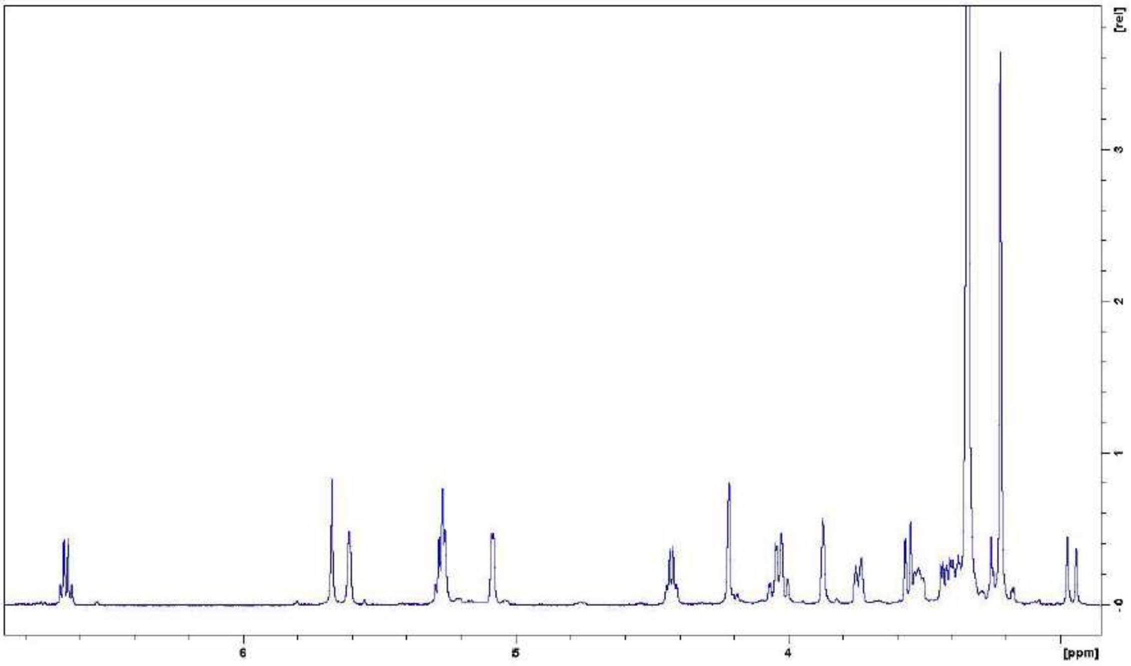

Figure S50. **Expansion of ^{1}H NMR spectrum of 13-*O*-deacetyl-13-*O*-paulyl-paulomycin B (15') (DMSO-d_6, 500 MHz, 24 °C).**

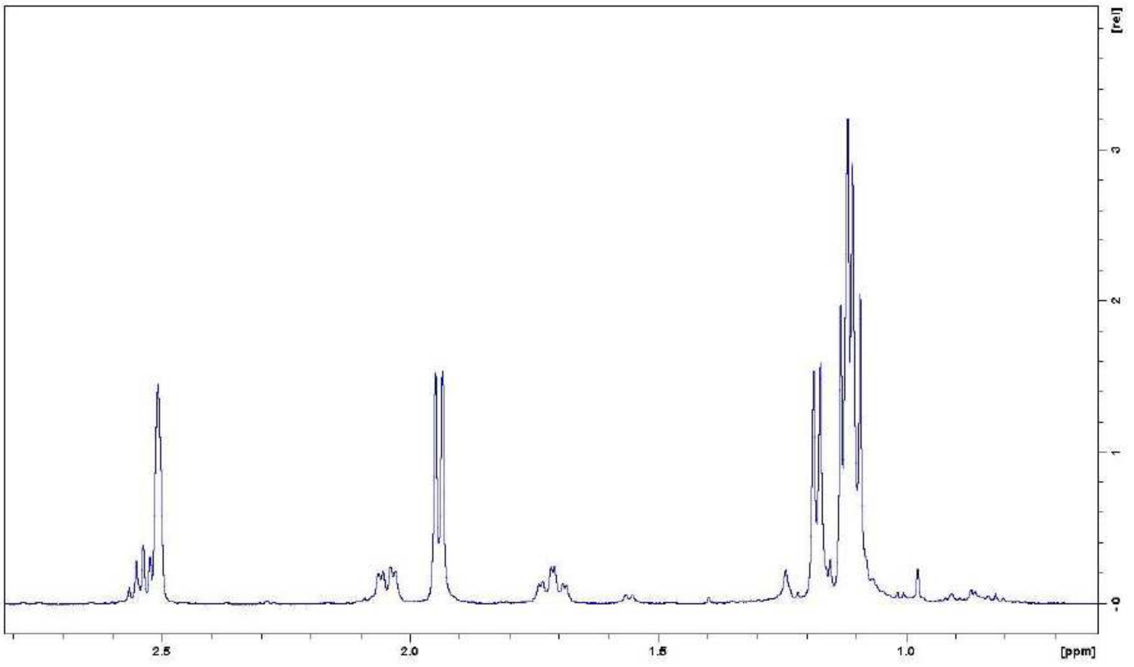

Figure S51. ^{1}H-^{13}C HSQC spectrum of 13-*O*-deacetyl-13-*O*-paulyl-paulomycin B (15') (DMSO-d_6, 500 MHz, 24 °C).

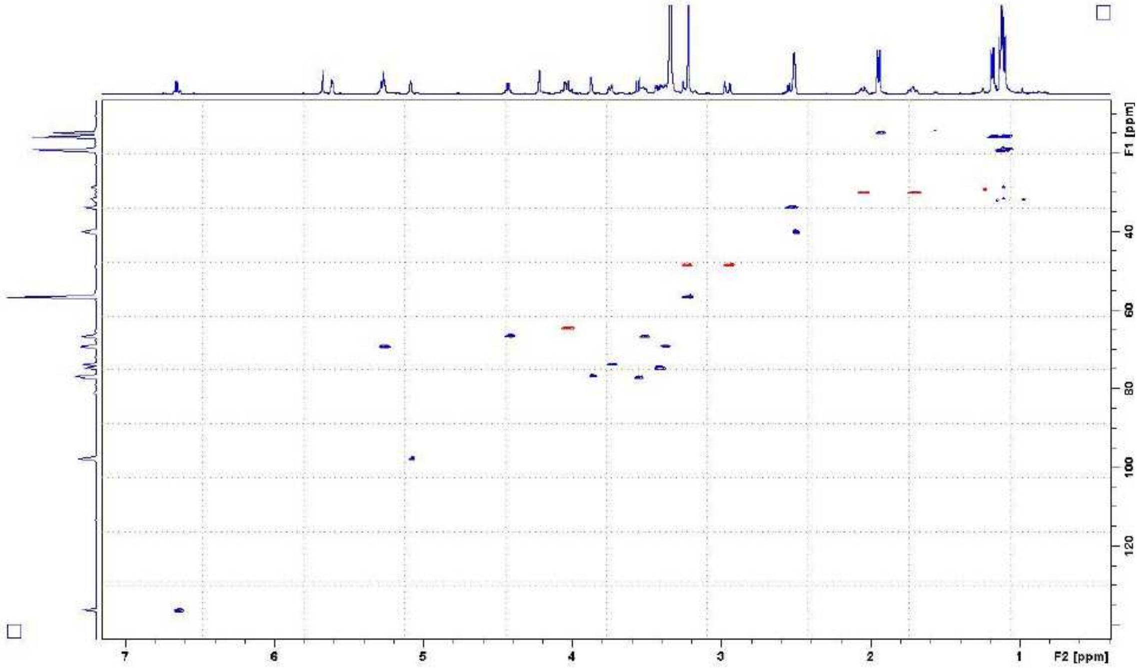

Figure S52. **Expansion of the ^{1}H-^{13}C HMBC spectrum of 13-*O*-deacetyl-13-*O*-paulyl-paulomycin B (15') (DMSO-d_6, 500 MHz, 24 °C).** The key correlation between carbon 1'' and protons at position 13 is highlighted in red color. The other HMBC correlations involving this carbonyl with the protons at positions 3'' and 4'' are highlighted in green color.

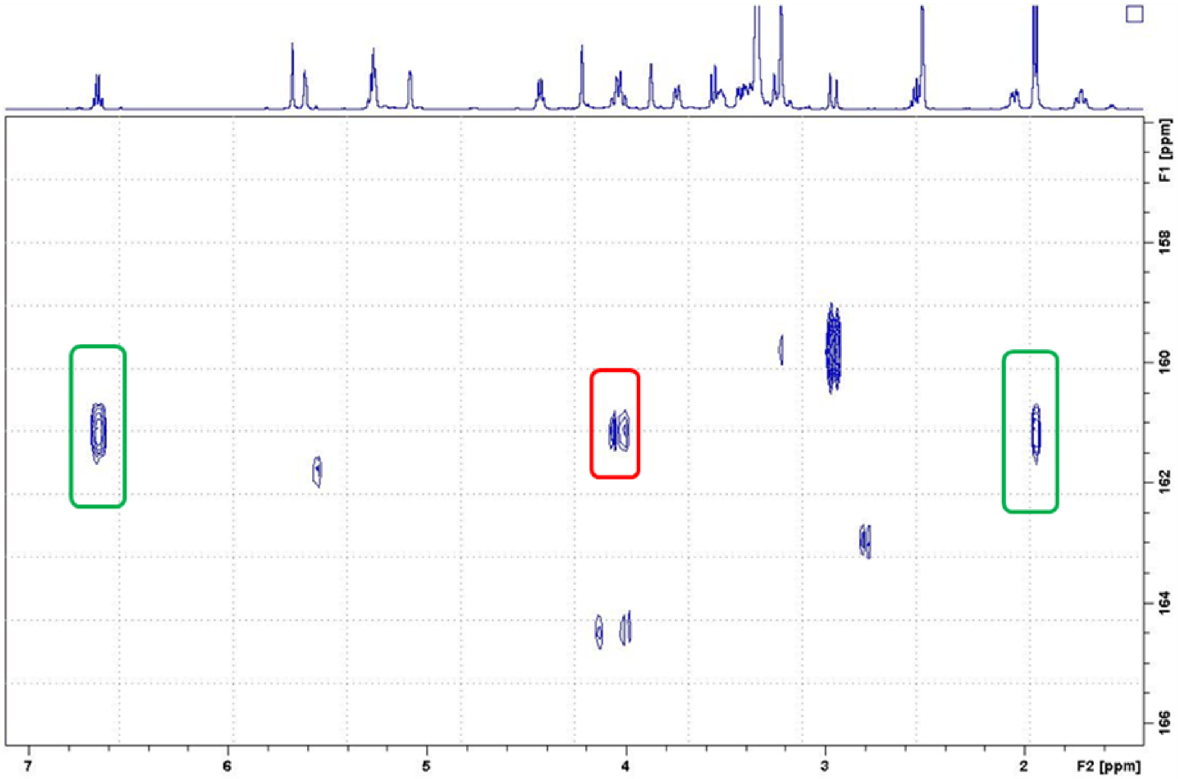

Figure S53. **Structure of 13-*O*-deacetyl-13-*O*-paulyl-paulomycin B (15').**

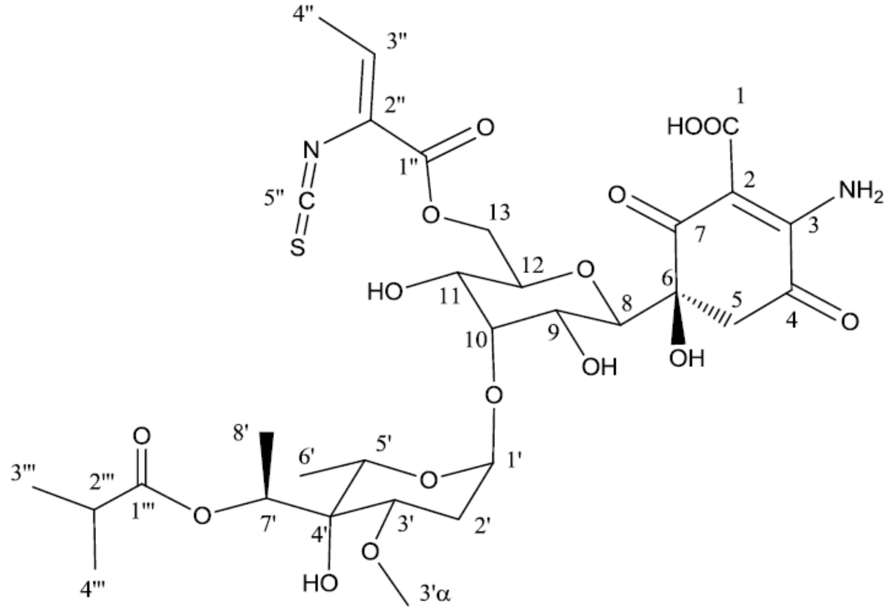

Table S7. 13-*O*-deacetyl-13-*O*-paulyl-paulomycin B (15') ^{13}C and ^{1}H NMR data (δ in ppm) acquired in DMSO-*d6* (500 MHz, 24 °C).

Position	δ ^{13}C	δ ^{1}H (mult, *J* in Hz)	Position	δ ^{13}C	δ ^{1}H (mult, *J* in Hz)
1	n.d.	-	1'	97.9	5.08 (d, 2.8)
2	99.5	-	2'	30.1	2.04 (dd, 11.9, 4.3)<br>1.71 (td, 12.0, 3.6)
3	159.8	-	3'	74.7	3.42 (dd, 11.6, 4.7)
4	189.0	-	3'α	56.7	3.22 (s)
5	48.6	3.23 (d, 16.1)<br>2.96 (d, 16.1)	4'	73.5	-
6	78.2	-	5'	66.6	4.43 (quart., 6.3)
7	198.5	-	6'	16.0	1.11 (d, 6.2)
8	77.3	3.56 (d, 9.9)	7'	69.4	5.27 (quart., 6.2)
9	69.2	3.38 (m, overlap)	8'	16.0	1.18 (d, 6.7)
10	76.9	3.87 (br t)	1''	161.1	-
11	66.8	3.52 (br t, 8.0)	2''	123.0	-
12	73.9	3.74 (br dt, 9.8)	3''	136.4	6.65 (quart., 7.0)
13	64.7	4.06 (dd, 12.0, 2.8)<br>4.01 (br d, 11.5)	4''	14.9	1.94 (d, 7.0)
			5''	n.d.	-
			1'''	175.9	-
			2'''	33.9	2.54 (sept., 6.8)
			3'''	19.2	1.10 (d, 6.8)
			4'''	19.3	1.12 (d, 6.8)

δ^{13}C were determined from HSQC and HMBC spectra.

Structural characterization of 13-*O*-deacetyl-13-*O*-paulyl-paulomycin A (16')

The HRMS data of compound **16'** confirmed a molecular formula of $C_{32}H_{44}N_2O_{16}S$ based on the observed pseudo molecular ion at *m/z* 745.2481 $[M+H]^+$ (Figure S54). The base peak in the mass spectrum does not correspond to the pseudo molecular ion but to the same fragment at *m/z* = 473.0867 $[M+H]^+$ (Figure S54) already found for 13-*O*-deacetyl-13-*O*-paulyl-paulomycin E (**14'**) and 13-*O*-deacetyl-13-*O*-paulyl-paulomycin B (**15'**). This suggested that 13-*O*-deacetyl-13-*O*-paulyl-paulomycin E (**14'**), 13-*O*-deacetyl-13-*O*-paulyl-paulomycin B (**15'**) and compound **16'** share the same molecular structure for that fragment.

To establish the connectivity and confirm the structure of **16'** the ^{1}H NMR spectrum and a set of 2D NMR spectra (including COSY, HSQC and HMBC) were acquired (Figures S55 to S60). As expected, most of the observed signals were identical to those observed for 13-*O*-deacetyl-13-*O*-paulyl-paulomycin B (**15'**). In depth interpretation of the correlations observed allowed establishing the structure of compound **16'** as 13-*O*-deacetyl-13-*O*-paulyl-paulomycin A (Figure S61, Table S8).

It was discarded the epimer at 3' (senfolomycin like configuration), based on the coupling constants displayed by the proton in position 3' and by comparison with the NMR data of paulomycin F (**12**), 13-*O*-deacetyl-13-*O*-paulyl-paulomycin E (**14'**) and 13-*O*-deacetyl-13-*O*-paulyl-paulomycin B (**15'**). As expected, the paulic acid unit is acylating the primary hydroxyl as it was found for 13-*O*-deacetyl-13-*O*-paulyl-paulomycin B (**15'**). On the other hand, the substituent at position 4' of the paulomycose unit matches that reported for paulomycin A [1]. The determined structure proves that the main fragment observed in the mass spectrum is identical to that found in 13-*O*-deacetyl-13-*O*-paulyl-paulomycin E (**14'**) and 13-*O*-deacetyl-13-*O*-paulyl-

paulomycin B (**15'**), which matches the structure determined for 6-hydroxyl-13-*O*-paulyl-paulinone (**6'**).

Figure S54. (+) ESI-TOF spectrum of 13-*O*-deacetyl-13-*O*-paulyl-paulomycin A (16').

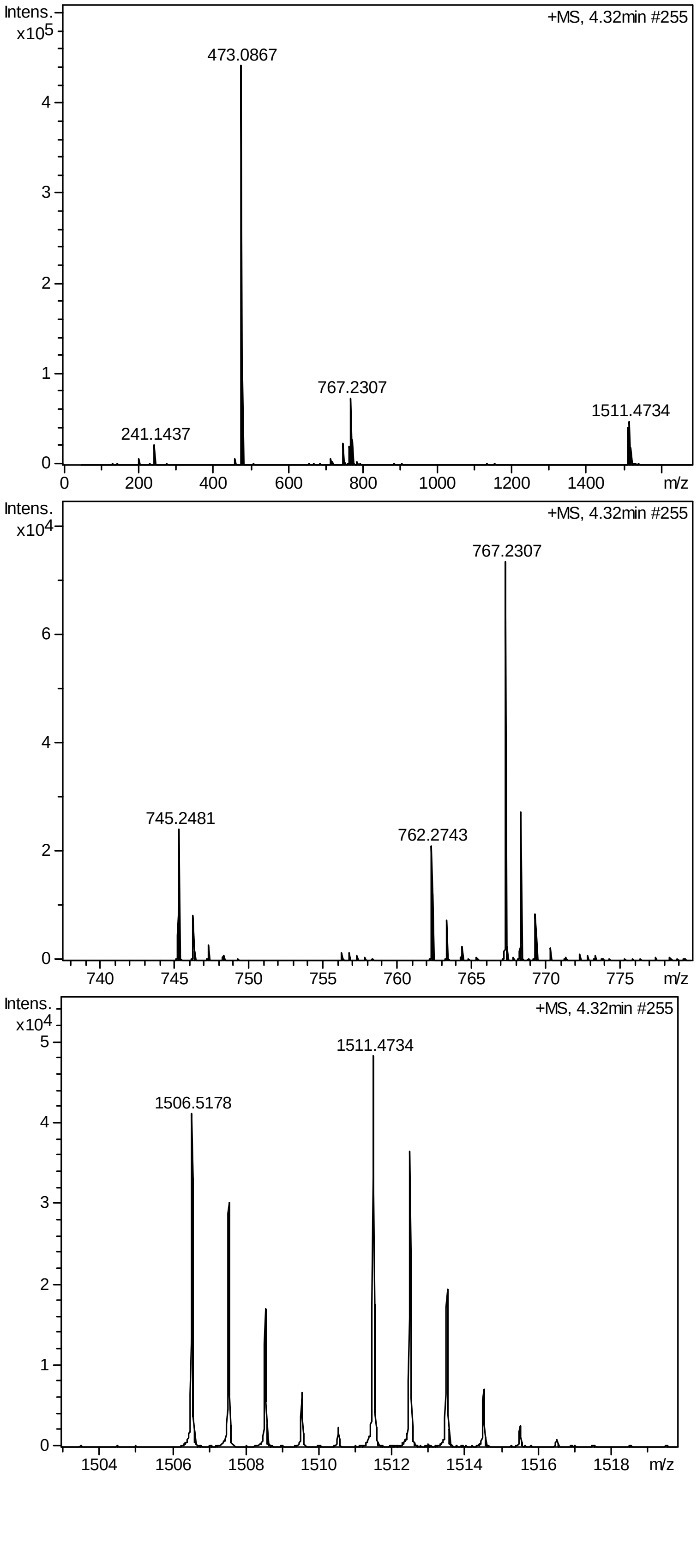

Figure S55. ^{1}H NMR spectrum of 13-*O*-deacetyl-13-*O*-paulyl-paulomycin A (16') (DMSO-d_6, 500 MHz, 24 °C).

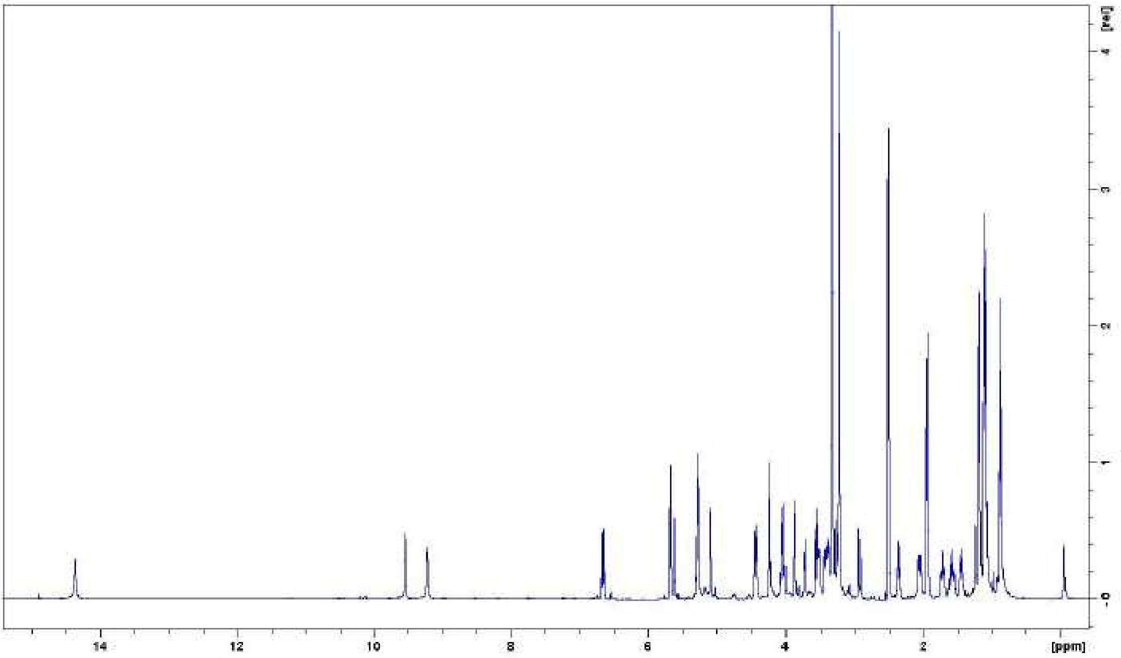

Figure S56. Expansion of ^{1}H NMR spectrum of 13-*O*-deacetyl-13-*O*-paulyl-paulomycin A (16') (DMSO-d_6, 500 MHz, 24 °C).

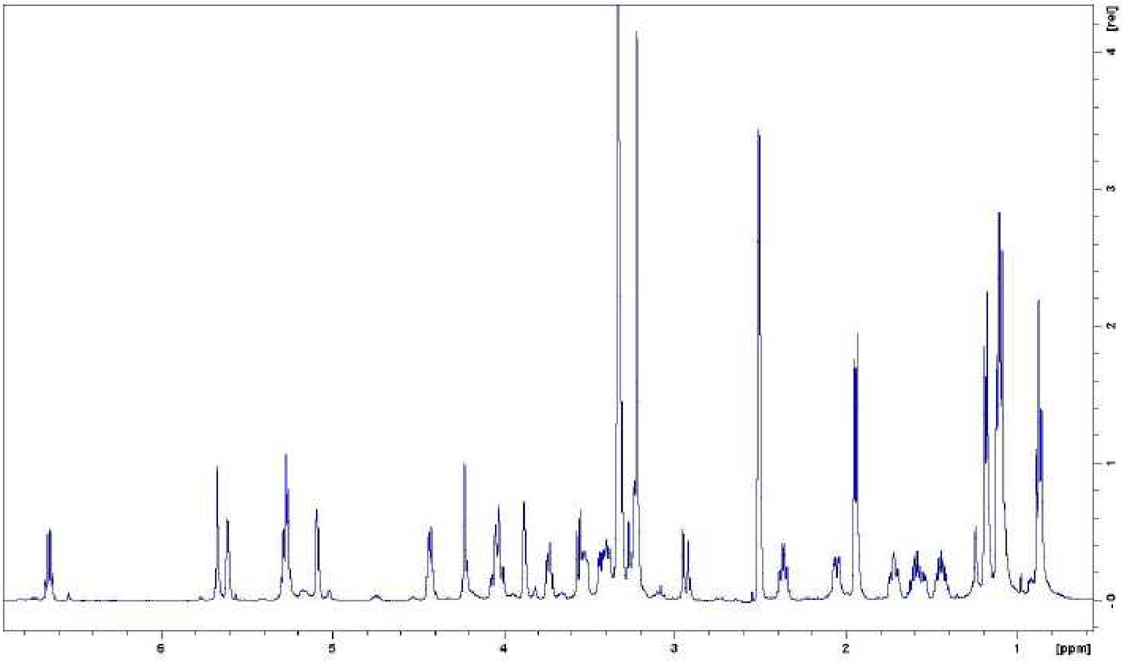

Figure S57. **Expansion of ^{1}H NMR spectrum of 13-*O*-deacetyl-13-*O*-paulyl-paulomycin A (16') (DMSO-*d*$_6$, 500 MHz, 24 °C).**

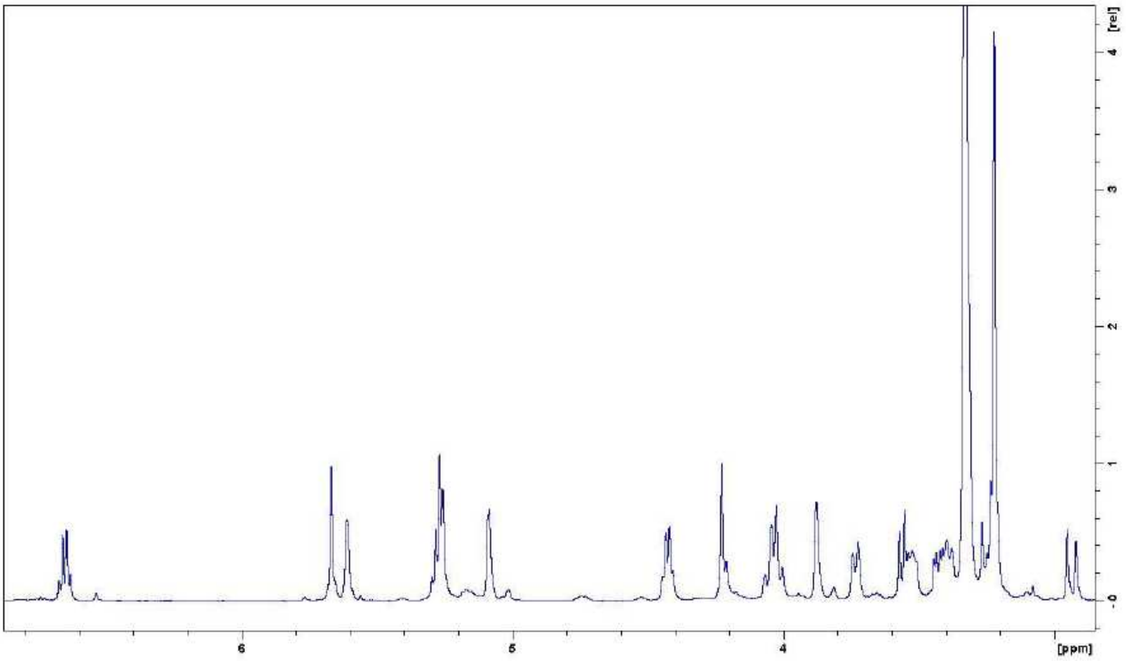

Figure S58. **Expansion of ^{1}H NMR spectrum of 13-*O*-deacetyl-13-*O*-paulyl-paulomycin A (16') (DMSO-*d*$_6$, 500 MHz, 24 °C).**

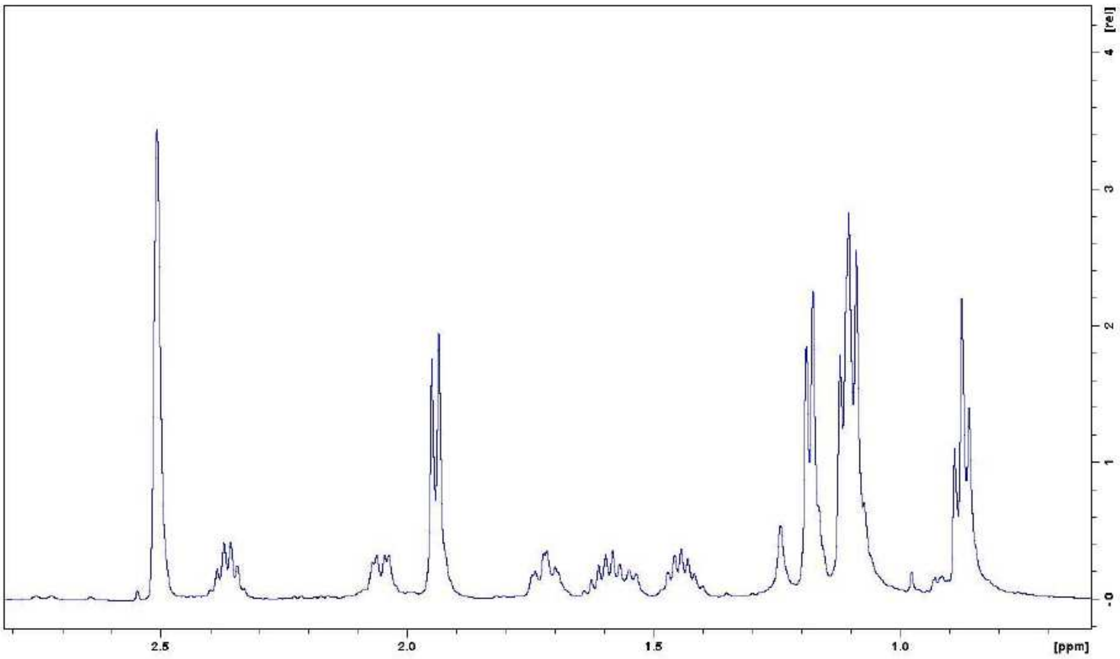

Figure S59. **^{1}H-^{13}C HSQC spectrum of 13-*O*-deacetyl-13-*O*-paulyl-paulomycin A (16') (DMSO-*d$_6$*, 500 MHz, 24 °C).**

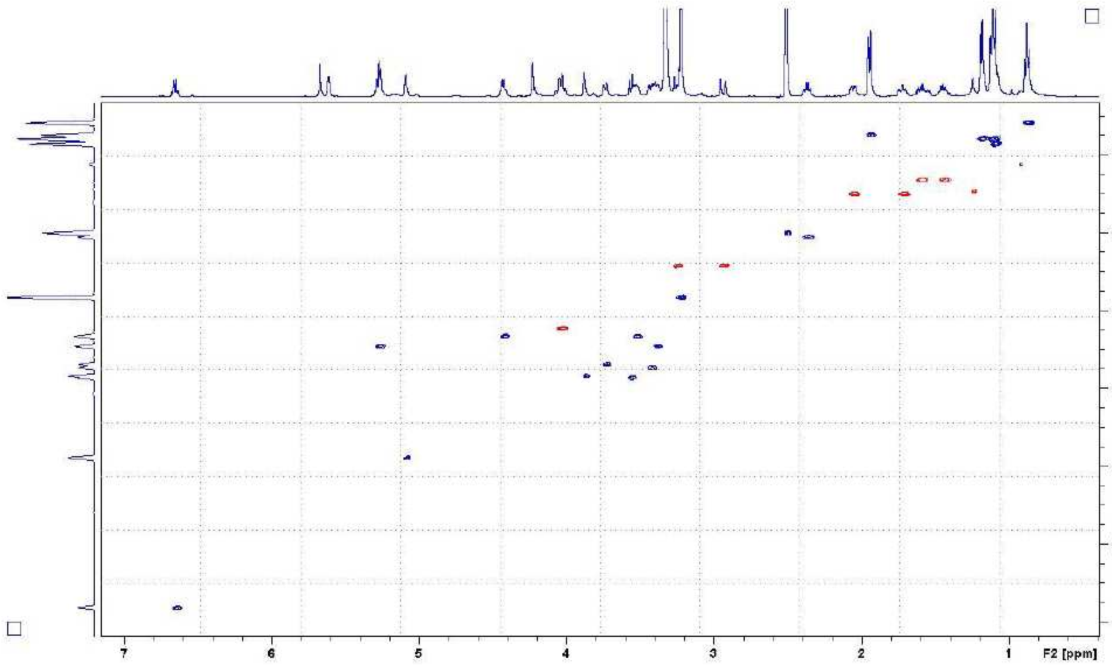

Figure S60. **Expansion of the ^{1}H-^{13}C HMBC spectrum of of 13-*O*-deacetyl-13-*O*-paulyl-paulomycin A (16') (DMSO-*d$_6$*, 500 MHz, 24 °C).** The key correlation between carbon 1'' and protons at position 13 is highlighted in red color. The other HMBC correlations involving this carbonyl with the protons at positions 3'' and 4'' are highlighted in green color. Better S/N is observed in the HMBC spectrum of **15'** for comparison.

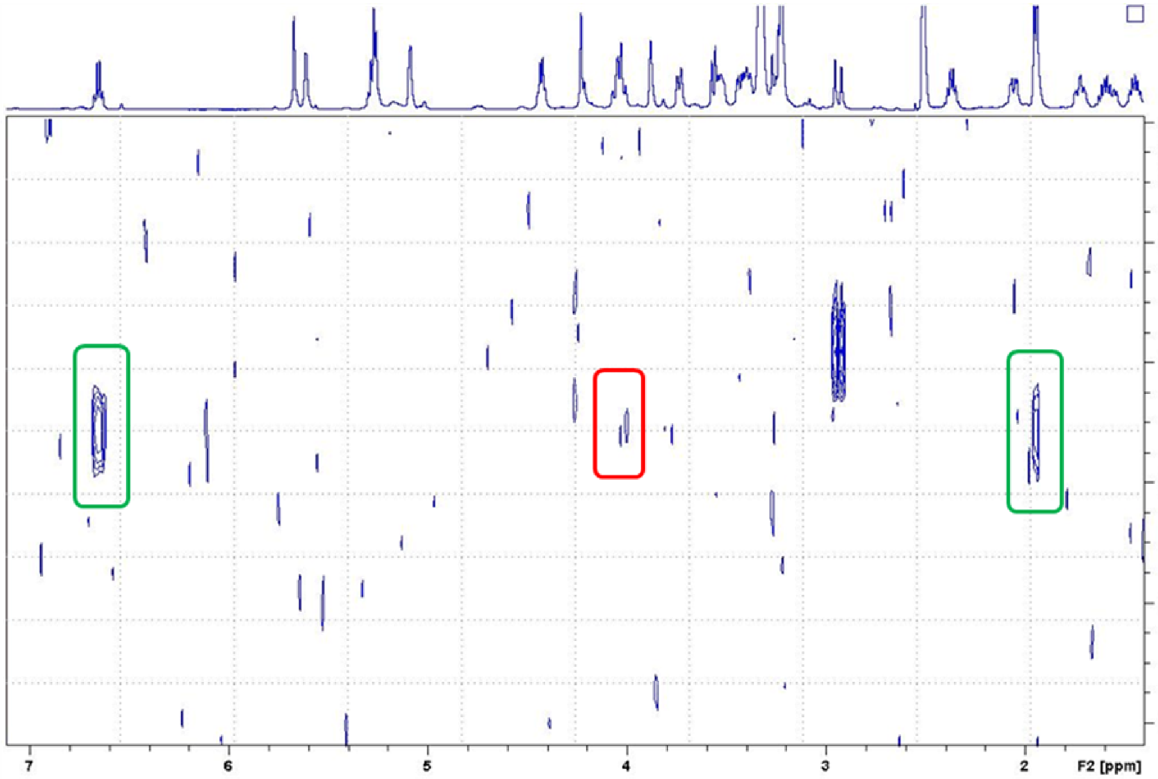

Figure S61. Structure of 13-*O*-deacetyl-13-*O*-paulyl-paulomycin A (16’).

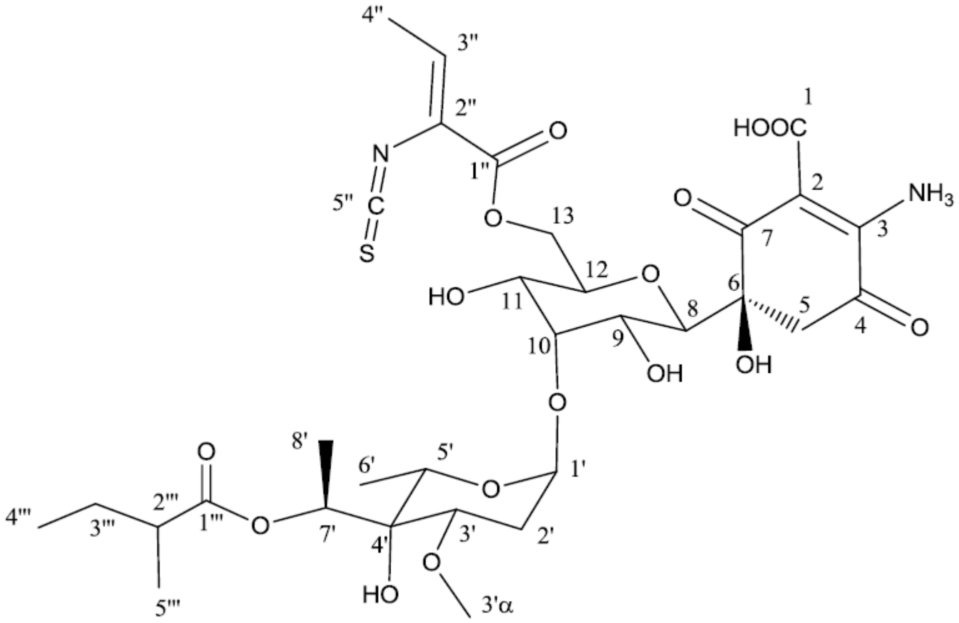

Table S8. 13-*O*-deacetyl-13-*O*-paulyl-paulomycin A (16’) ^{13}C and ^{1}H NMR data (δ in ppm) acquired in DMSO-*d6* (500 MHz, 24 °C).

Position	δ ^{13}C	δ ^{1}H (mult, *J* in Hz)	Position	δ ^{13}C	δ ^{1}H (mult, *J* in Hz)
1	n.d.	-	1’	97.9	5.08 (d, 2.8)
2	99.5	-	2’	30.1	2.04 (dd, 11.9, 4.3)<br>1.71 (td, 12.0, 3.6)
3	159.8	-	3’	74.7	3.42 (dd, 11.6, 4.7)
4	189.0	-	3’α	56.7	3.22 (s)
5	48.6	3.23 (d, 16.1)<br>2.96 (d, 16.1)	4’	73.5	-
6	78.2	-	5’	66.6	4.43 (quart., 6.3)
7	198.5	-	6’	16.0	1.11 (d, 6.2)
8	77.3	3.56 (d, 9.9)	7’	69.4	5.27 (quart., 6.2)
9	69.2	3.38 (m, overlap)	8’	16.0	1.18 (d, 6.7)
10	76.9	3.87 (br t)	1’’	161.1	-
11	66.8	3.52 (br t, 8.0)	2’’	123.0	-
12	73.9	3.74 (br dt, 9.8)	3’’	136.4	6.65 (quart., 7.0)
13	64.7	4.06 (dd, 12.0, 2.8)<br>4.01 (br d, 11.5)	4’’	14.9	1.94 (d, 7.0)
			5’’	n.d.	-
			1’’’	175.9	-
			2’’’	41.2	2.36 (sext. 6.8)
			3’’’	26.4	1.59 (hept. 6.8)<br>1.44 (hept. 6.8)
			4’’’	11.7	0.87 (t, 7.0)
			5’’’	17.2	1.09 (d, 7.0)

δ^{13}C were determined from HSQC and HMBC spectra.

Structural characterization of 3'-*O*-demethyl-paulomycin E (17)

The molecular formula of compound **17**, $C_{28}H_{34}N_2O_{16}S$, was established based on the pseudomolecular ion peak $[M+H]^+$ observed at 687.1708 (Figure S62). As expected, the base peak in the mass spectrum does not correspond to the pseudomolecular ion but to a fragment at *m/z* = 515.0966. The presence of such diagnostic fragment once more confirmed the paulomycin nature of the molecule. Unfortunately in the ^1H and the HSQC NMR spectra (Figures S63-S66) was very difficult to identify characteristic paulomycin like signals because the sample was in small amounts and apparently strongly polymerized (very broad signals dominate the proton spectrum).

Due to the appearance of the spectra it was obvious that NMR based structural elucidation was impossible and thus no additional 2D NMR experiments were acquired. Nevertheless, based on the LC-DAD-HRMS data and the structure of paulomycins found in compounds **18** and **19** (see below) a structural hypothesis could be proposed (Figure S67).

As already mentioned, the base peak in the mass spectrum correspond to the common fragment at *m/z* = "515". This fragment displays identical molecular structure for all known paulomycins, thus assuming a common biosynthetic route, it can be concluded that in compound **17** the "515" fragment will indeed share the same structure as found along the series of known paulomycins. Thus to meet the molecular formula the carbohydrate (paulomycose) unit must display the same connectivity found in paulomycin E. Thus the structure would correspond to what could be named 3'-*O*-demethyl-paulomycin E. Since, according to our data, paulomycin E is proposed to be a precursor of paulomycins A, B and F, and assuming that compounds **17**, **18** (3'-*O*-demethyl-paulomycin B) and **19** (3'-*O*-demethyl-paulomycin A) are produced by the same strain, thus it seems reasonable to think that the structure of compound **17** may

indeed correspond to 3'-*O*-demethyl-paulomycin E (Figure S67), the expected biosynthetic precursor for 3'-*O*-demethyl-paulomycins A and B (compounds **19** and **18** respectively, see below).

Figure S62. **(+) ESI-TOF spectrum of proposed 3'-*O*-demethyl-paulomycin E (17).** The UV (DAD) spectrum of the main component perfectly matches that reported for paulomycins [7].

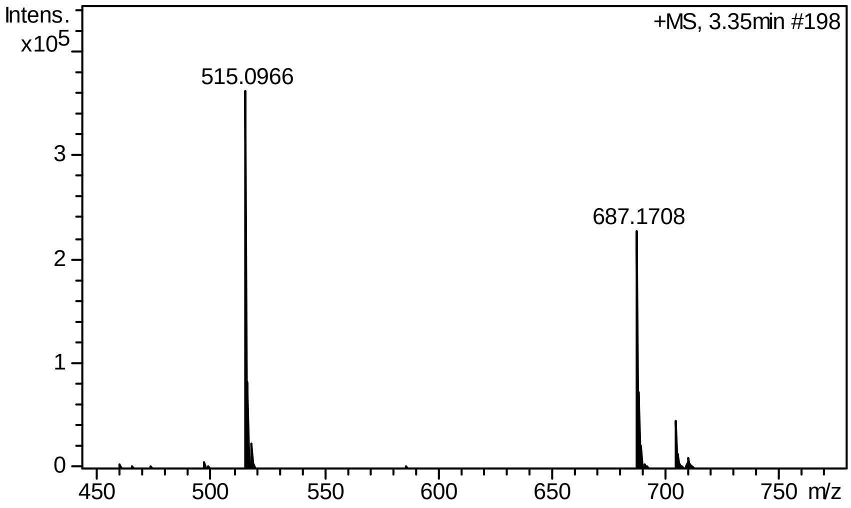

Figure S63. **^{1}H NMR spectrum of proposed 3'-*O*-demethyl-paulomycin E (17) (DMSO-d_6, 500 MHz, 24 °C).**

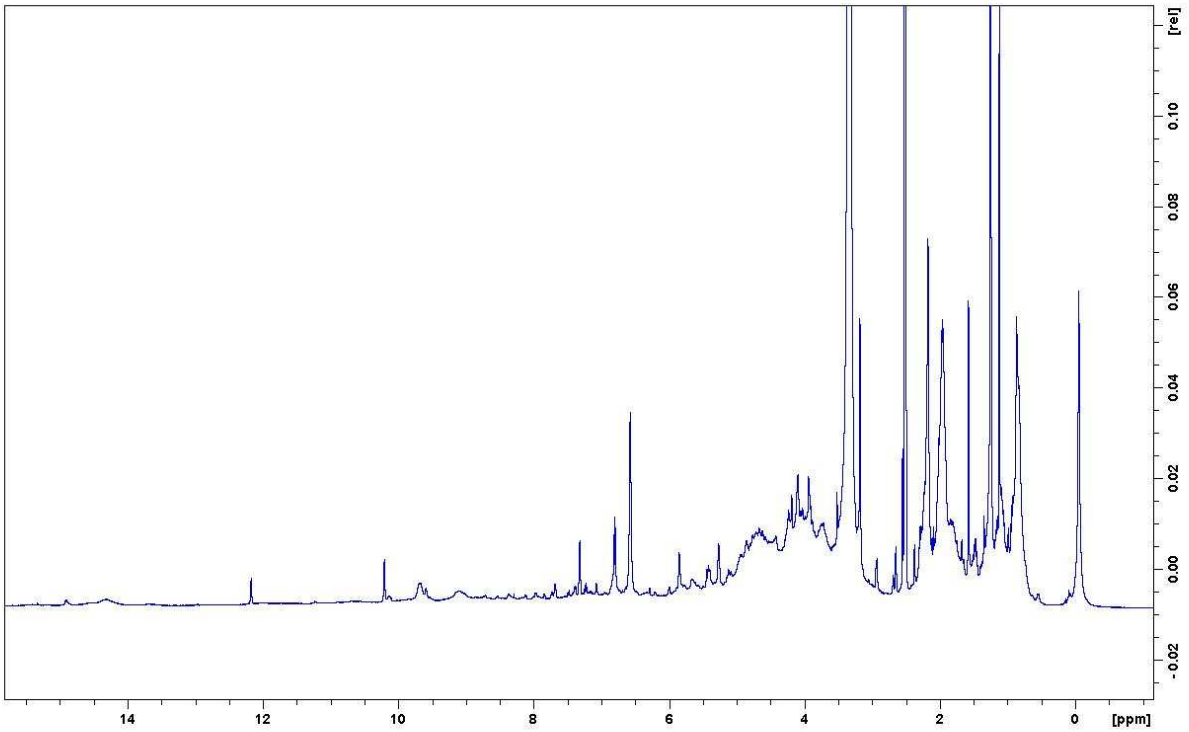

Figure S64. **Expansion of ^{1}H NMR spectrum of proposed 3'-*O*-demethyl-paulomycin E (17) (DMSO-d_6, 500 MHz, 24 °C).**

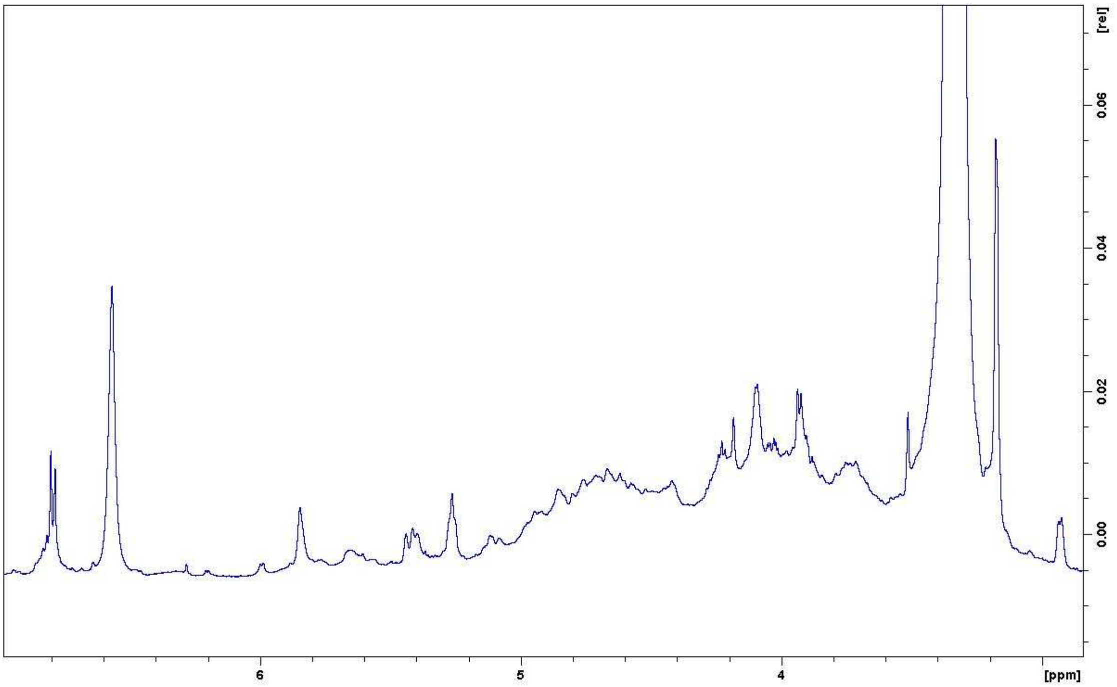

Figure S65. **Expansion of ^{1}H NMR spectrum of proposed 3'-*O*-demethyl-paulomycin E (17) (DMSO-d_6, 500 MHz, 24 °C).**

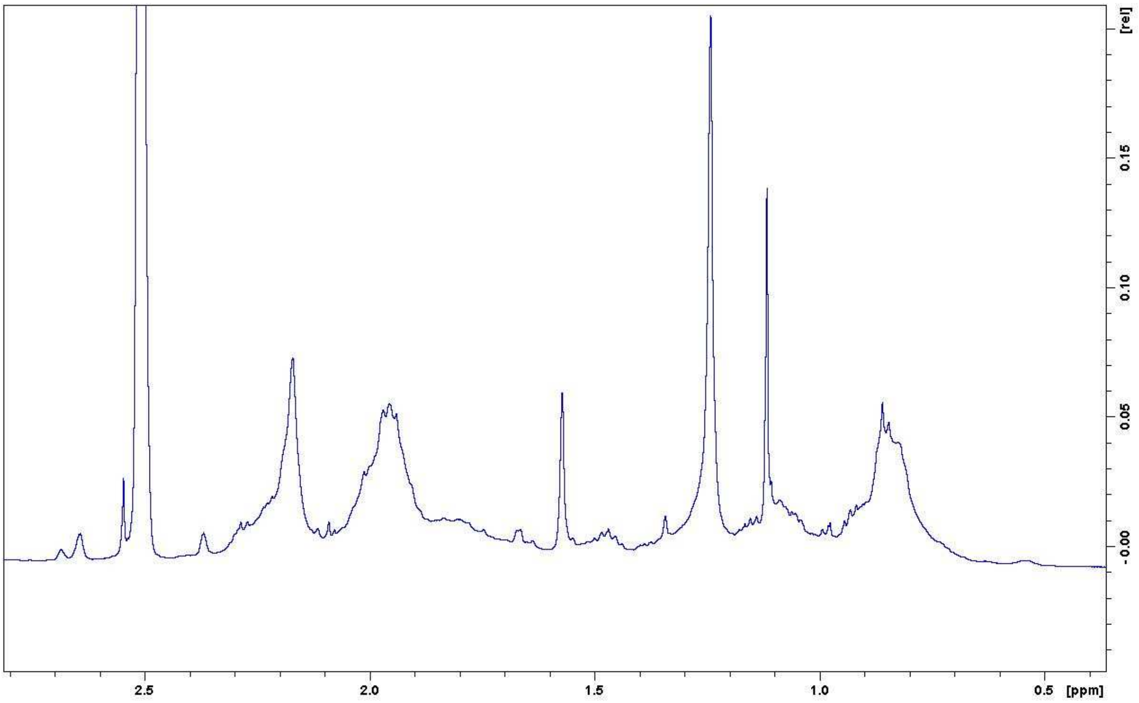

Figure S66. [1]**H-**[13]**C HSQC spectrum of proposed 3'-*O*-demethyl-paulomycin E (17) (DMSO-d_6, 500 MHz, 24 °C).**

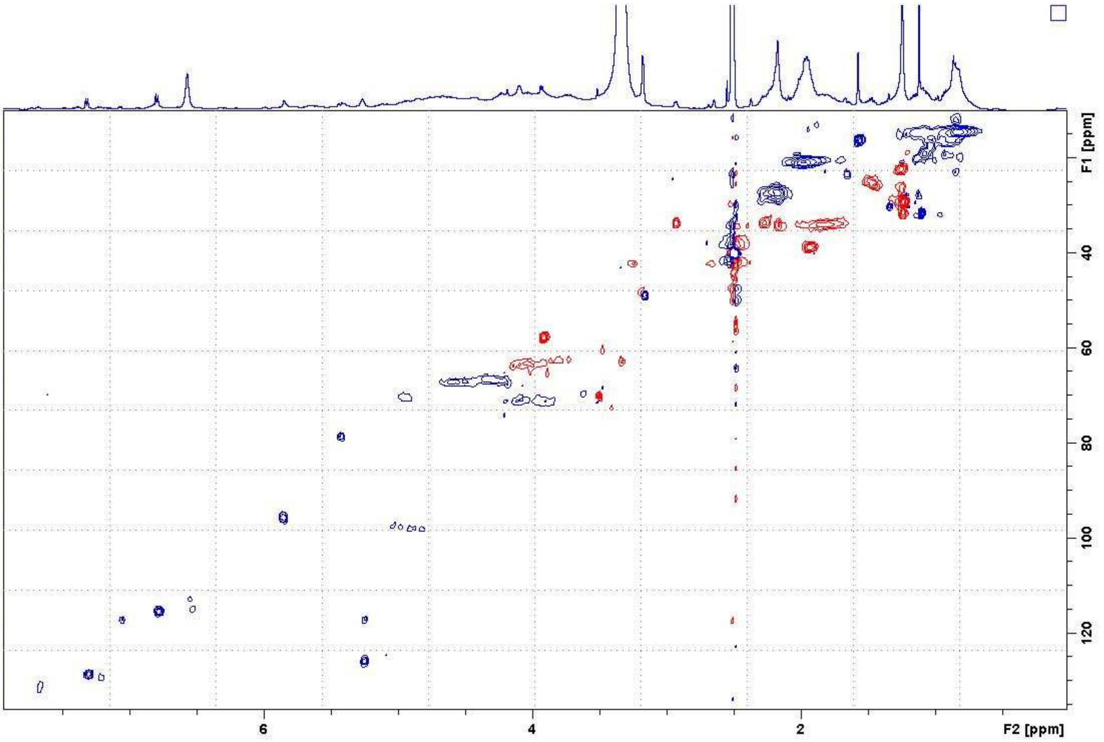

Figure S67. Structure of proposed 3'-*O*-demethyl-paulomycin E (17). Structure of fragment at *m/z* = "515" is shown in blue and the proposal for the paulomycose moiety is drawn in red color.

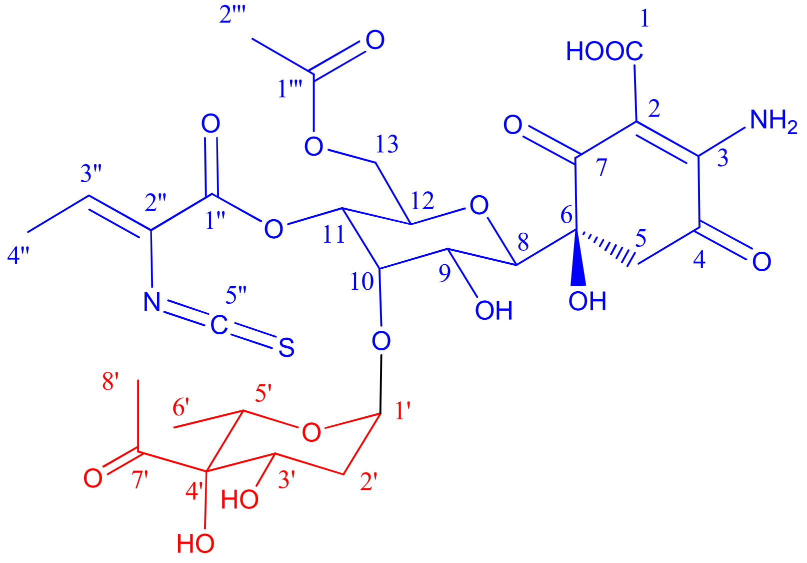

Structural characterization of 3'-*O*-demethyl-paulomycin B (18)

The molecular formula of compound **18**, $C_{32}H_{42}N_2O_{17}S$, was established based on the pseudomolecular ion peak $[M+H]^+$ observed at 759.2291 (Figure S68). The base peak in the mass spectrum at *m/z* = 515.0972 once more confirmed the paulomycin nature of the molecule. The ^{1}H and the HSQC NMR spectra (Figures S69-S73) were almost identical to those of compound **19** (see below) and the differences were only found in the aliphatic signals from the acid that esterifies the carbohydrate at position 7'.

The establishment of the structure required the acquisition of additional 2D NMR spectra including, COSY, TOCSY, NOESY and HMBC. After analysis of such spectral set, the structure of the main compound was determined and corresponds to 3'-*O*-demethyl-paulomycin B (Figure S74). Once again, based on the coupling constants displayed by the proton at position 3' (11.4 and 4.7 Hz) a senfolomycin like configuration at this center of the paulomycose sugar was discarded.

Figure S68. (+) ESI-TOF spectrum of 3'-*O*-demethyl-paulomycin B (18).

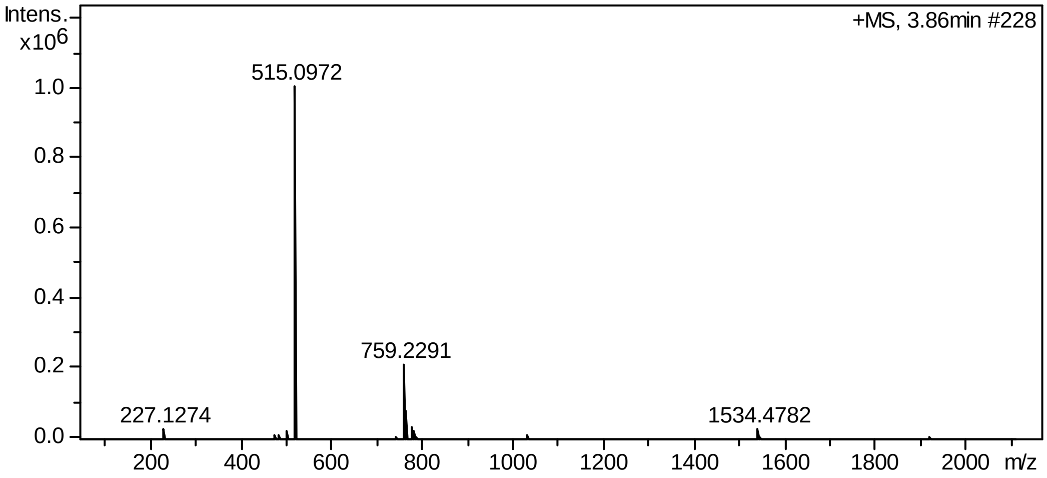

Figure S69. ^{1}**H NMR spectrum of 3'-*O*-demethyl-paulomycin B (18) (DMSO-*d*$_6$, 500 MHz, 24 °C).**

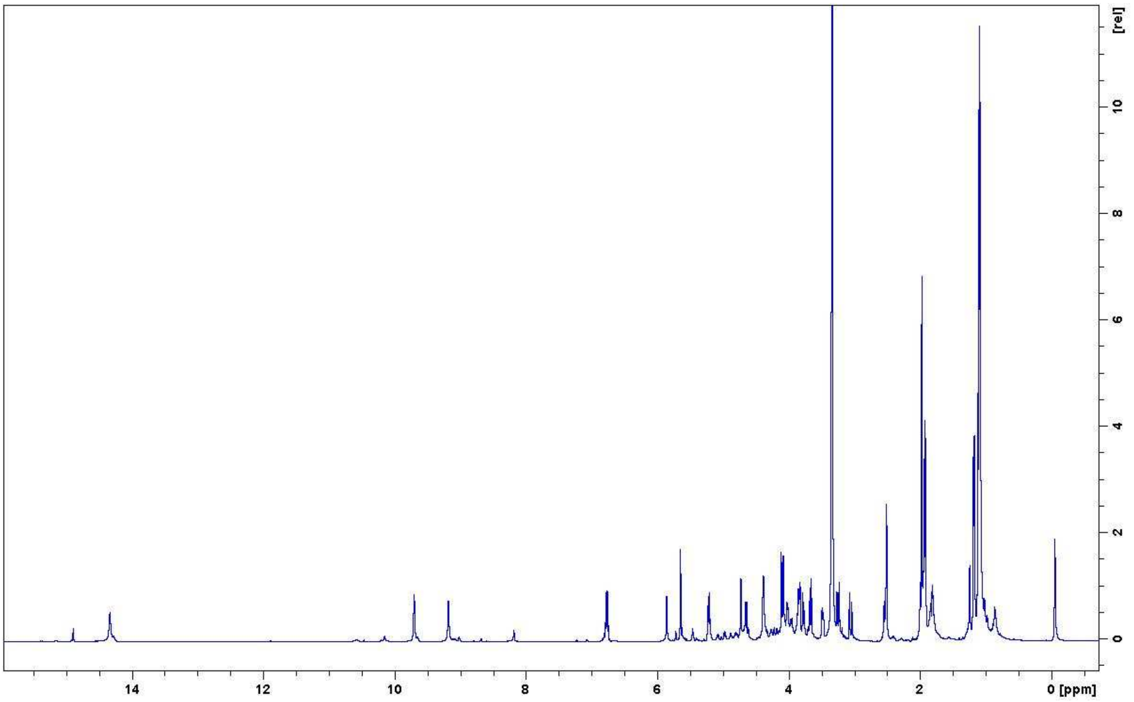

Figure S70. Expansion of ^{1}**H NMR spectrum of 3'-*O*-demethyl-paulomycin B (18) (DMSO-*d*$_6$, 500 MHz, 24 °C).**

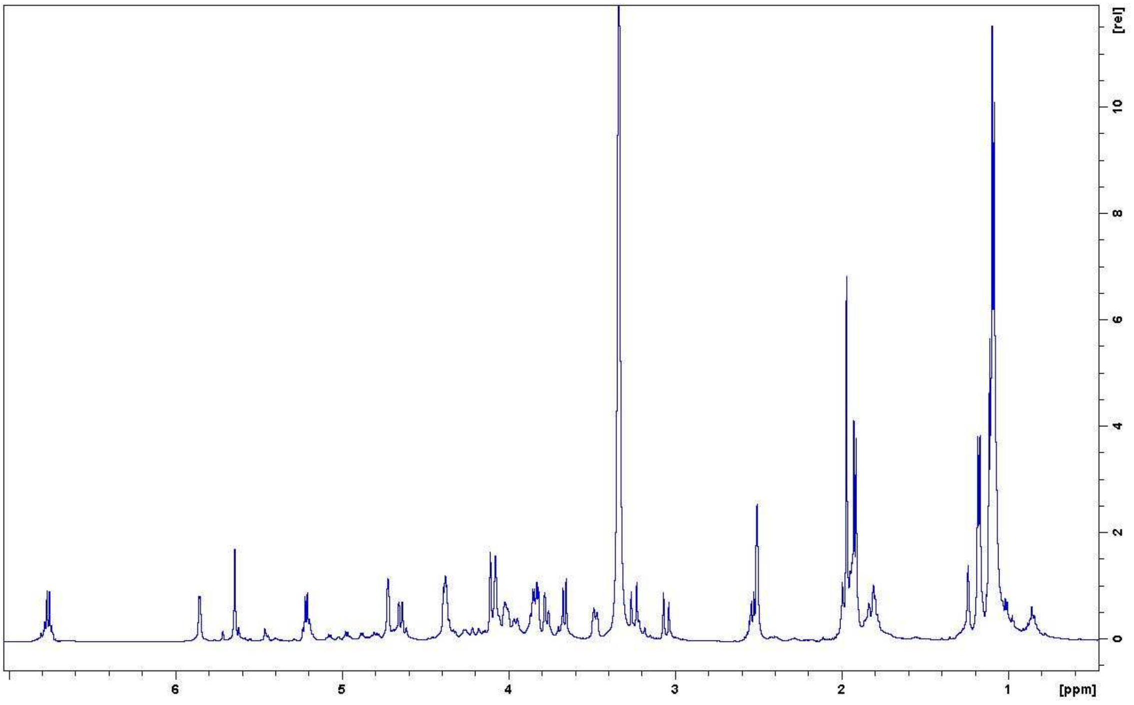

Figure S71. Expansion of ¹H NMR spectrum of 3’-*O*-demethyl-paulomycin B (18) (DMSO-d_6, 500 MHz, 24 °C).

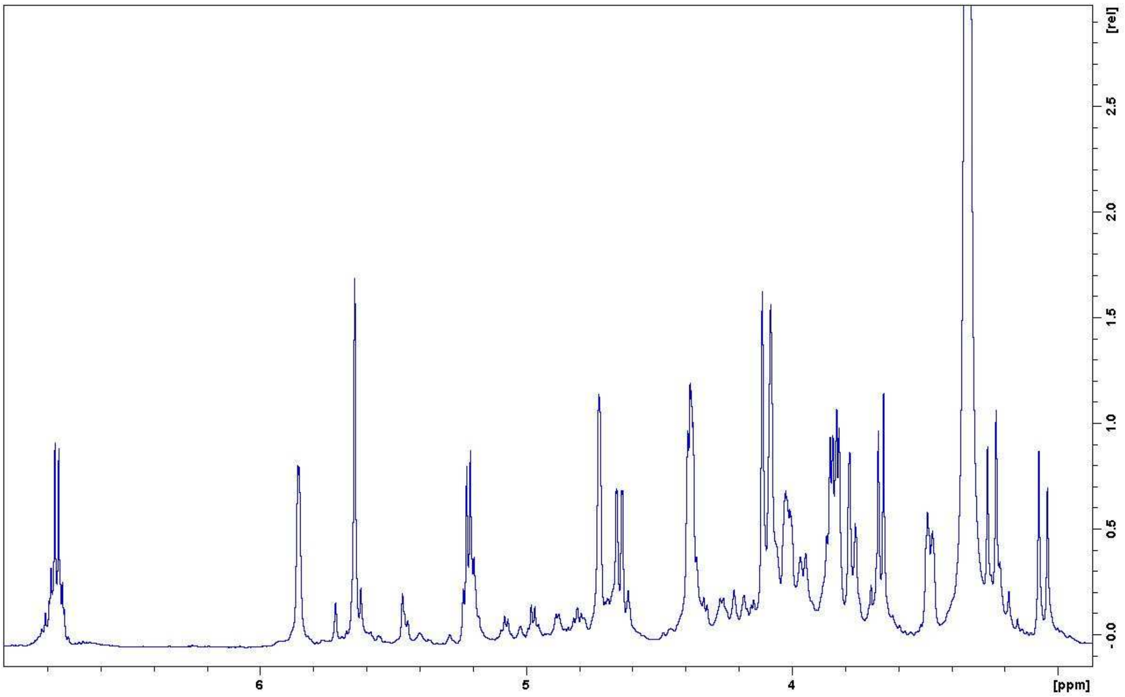

Figure S72. Expansion of ¹H NMR spectrum of 3’-*O*-demethyl-paulomycin B (18) (DMSO-d_6, 500 MHz, 24 °C).

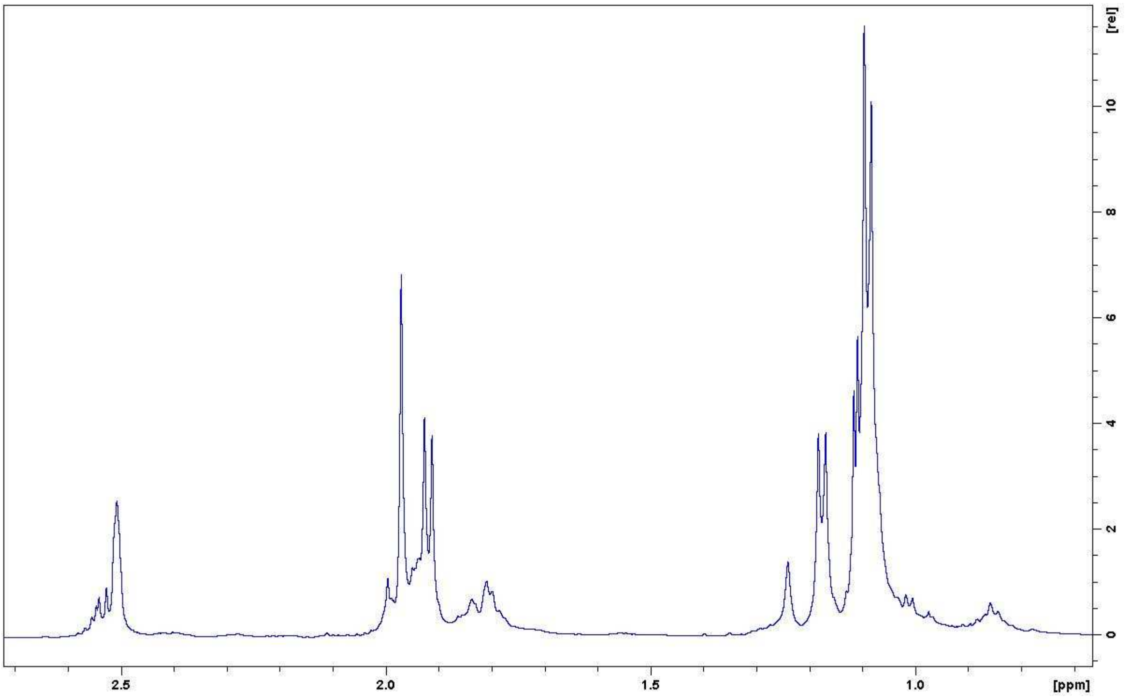

Figure S73. ^{1}H-^{13}C HSQC spectrum of 3'-*O*-demethyl-paulomycin B (18) (DMSO-*d*$_6$, 500 MHz, 24 °C).

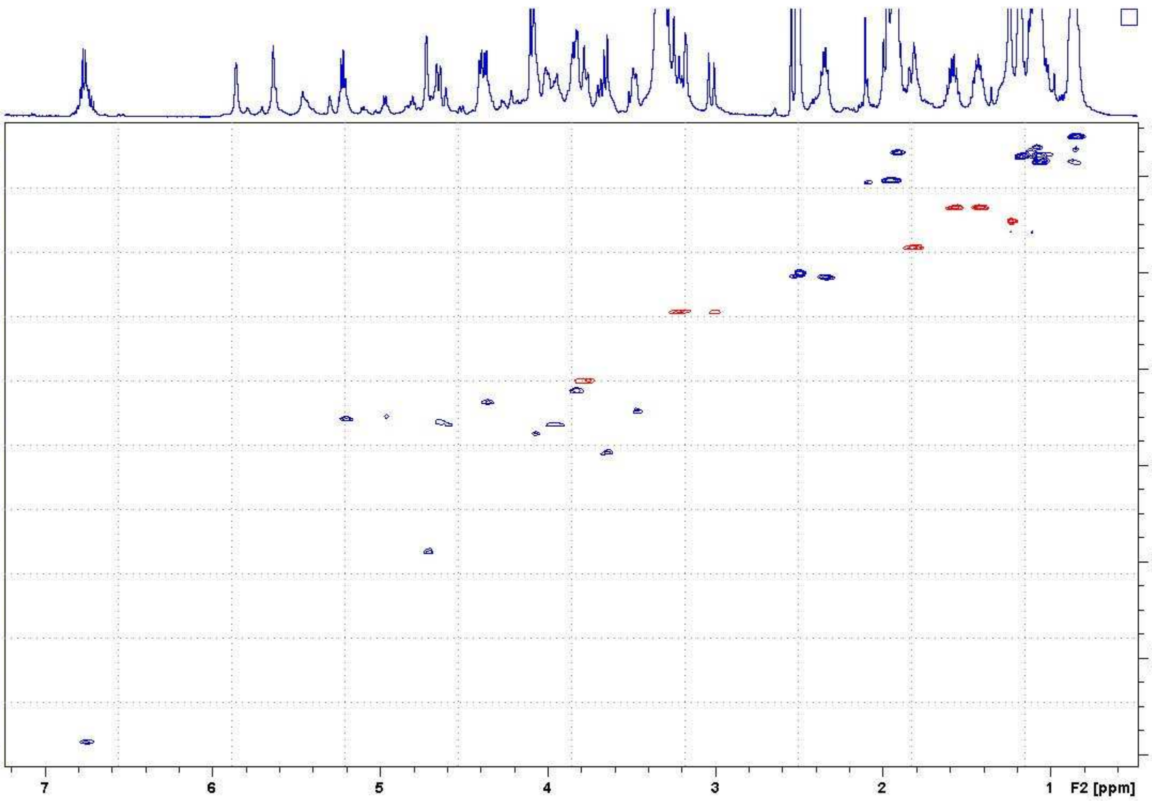

Figure S74. Structure of 3'-*O*-demethyl-paulomycin B (18).

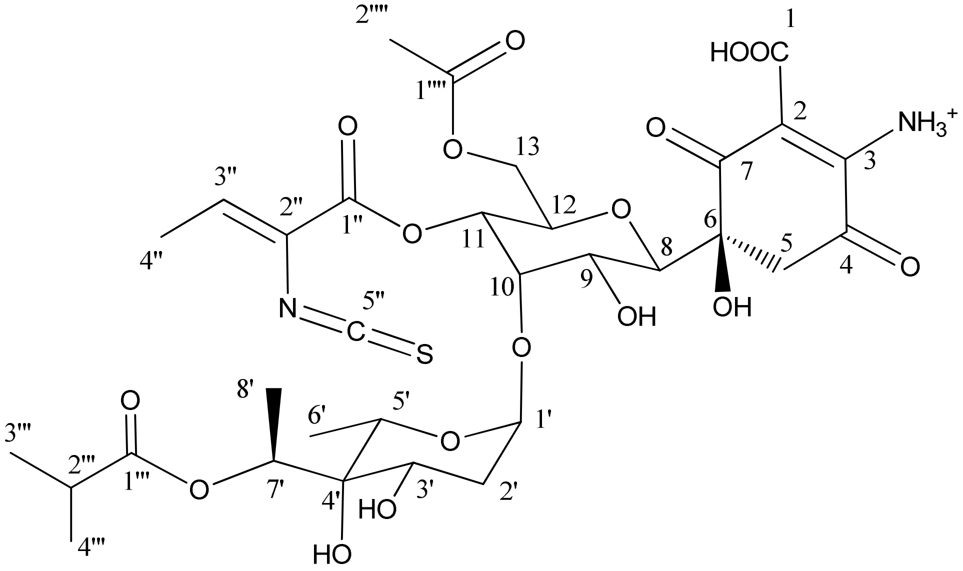

Table S9. 3’-*O*-demethyl-paulomycin B (18) ^{13}C and ^{1}H NMR data (δ in ppm) acquired in DMSO-*d6* (500 MHz, 24 °C).

Pos.	δ (^{13}C)	δ (^{1}H), (mult, *J* in Hz)	Pos.	δ (^{13}C)	δ(^{1}H), (mult, *J* in Hz)
1	n.d.	-	1’’	160.2	-
2	99.6	-	2’’	122.7	-
3	159.5	-	3’’	137.4	6.76 (quart., 7.1)
4	188.9	-	4’’	15.1	1.92 (d, 7.1)
5	48.2	3.25 (d, 16.1)<br>3.05 (d, 16.1)	5’’	141.9	-
6	77.8	-	1’’’	175.7	-
7	198.2	-	2’’’	33.8	2.53 (sext., 6.8)
8	77.3	3.66 (d, 9.8)	3’’’	19.5	1.09 (t, 6.9)
9	68.7	3.48 (br d, 9.5)	4’’’	19.5	1.09 (t, 6.9)
10	73.5	4.08 (m)	1’’’’	170.4	-
11	71.1	4.65 (dd, 10.0, 1.6)	2’’’’	20.8	1.97 (s)
12	71.5	4.01 (m)			
13	62.4	3.83 (m)<br>3.77 (dd, 12.0, 1.7)			
1’	97.9	4.72 (br s)			
2’	34.8	1.82 (m)			
3’	64.5	3.84 (dd, 11.1, 4.7)			
4’	73.7	-			
5’	66.9	4.38 (m)			
6’	15.8	1.09 (d, 6.5)			
7’	70.4	5.21 (quart., 6.7)			
8’	15.9	1.18 (d, 6.6)			

δ^{13}C were determined from HSQC and HMBC spectra.

Structural characterization of 3’-*O*-demethyl-paulomycin A (19)

The molecular formula of compound **19**, $C_{33}H_{44}N_2O_{17}S$, was established based on the pseudomolecular ion peak $[M+H]^+$ observed at 773.2448 (Figure S75). As expected, the base peak in the mass spectrum does not correspond to the pseudomolecular ion but to a fragment at *m/z* = 515.0971. The presence of such diagnostic fragment further confirmed the paulomycin nature of the molecule (the “515” fragment display the same molecular structure in the paulomycin series). The ^{1}H and the HSQC NMR (Figures S76-S80) spectra confirmed the paulomycin-like connectivity and, as expected, many of the observed signals show a strong resemblance to those displayed by paulomycin F (**12**), and compounds 20 and 21 (see below). The presence of the paulic acid moiety was

confirmed in the spepectra. Interestingly the spectra immediately revealed the absence of methoxyl groups in the structure of compound **19**.

The establishment of the structure required the acquisition of additional 2D NMR spectra including, COSY, TOCSY, NOESY and HMBC. After detailed analysis of such spectral set, the structure of the main compound was determined and it corresponds to 3'-*O*-demethyl-paulomycin A (Figure S81). Based on the coupling constants displayed by the proton at position 3' (11.1 and 4.4 Hz), a senfolomycin like configuration (OH in the axial position) was discarded at this center of the paulomycose sugar in a similar manner as it was determined for paulomycin F (**12**).

Figure S75. (+) ESI-TOF spectrum of 3'-*O*-demethyl-paulomycin A (19).

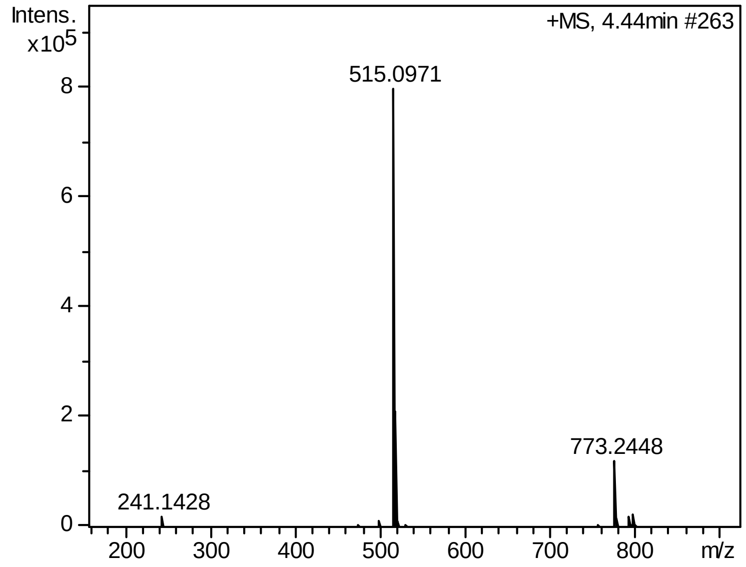

Figure S76. ^{1}H NMR spectrum of 3’-*O*-demethyl-paulomycin A (19) (DMSO-d_6, 500 MHz, 24 °C).

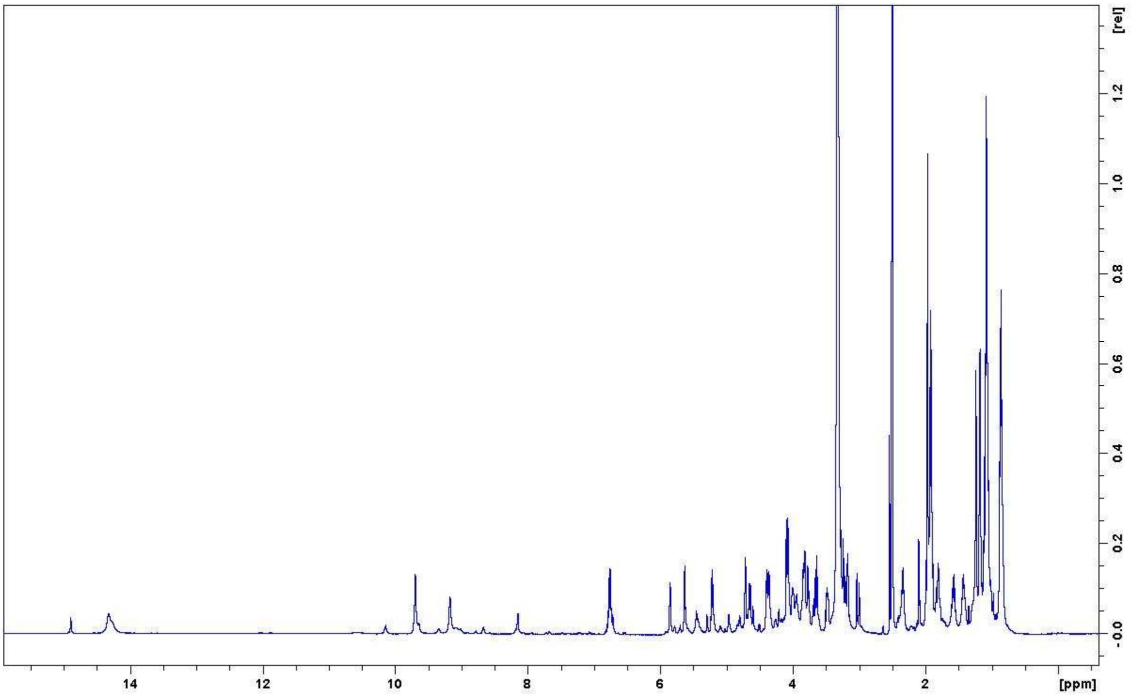

Figure S77. Expansion of ^{1}H NMR spectrum of 3’-*O*-demethyl-paulomycin A (19) (DMSO-d_6, 500 MHz, 24 °C).

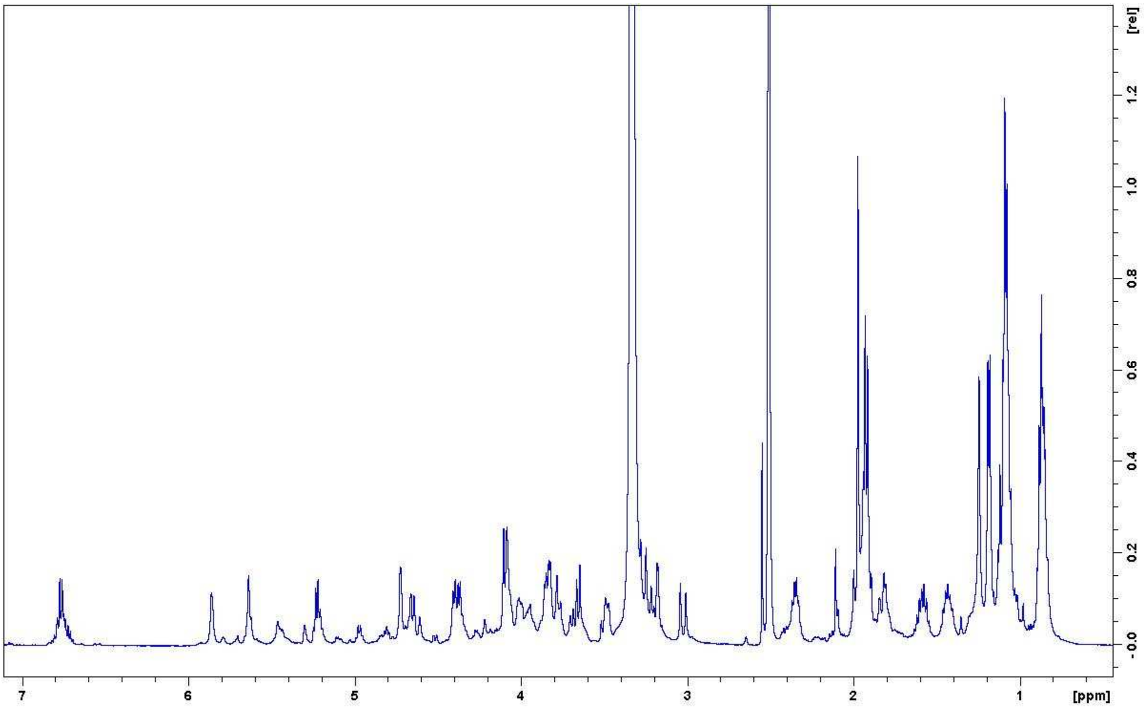

Figure S78. Expansion of ^{1}H NMR spectrum of 3'-*O*-demethyl-paulomycin A (19) (DMSO-d_6, 500 MHz, 24 °C).

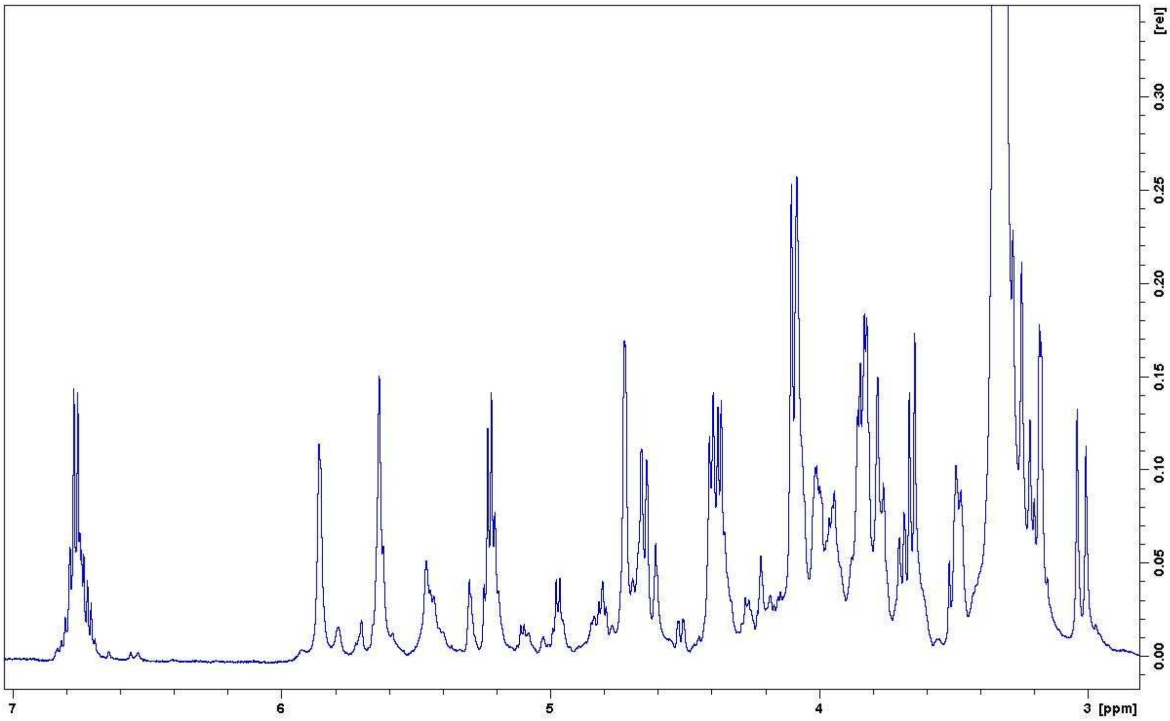

Figure S79. Expansion of ^{1}H NMR spectrum of 3'-*O*-demethyl-paulomycin A (19) (DMSO-d_6, 500 MHz, 24 °C).

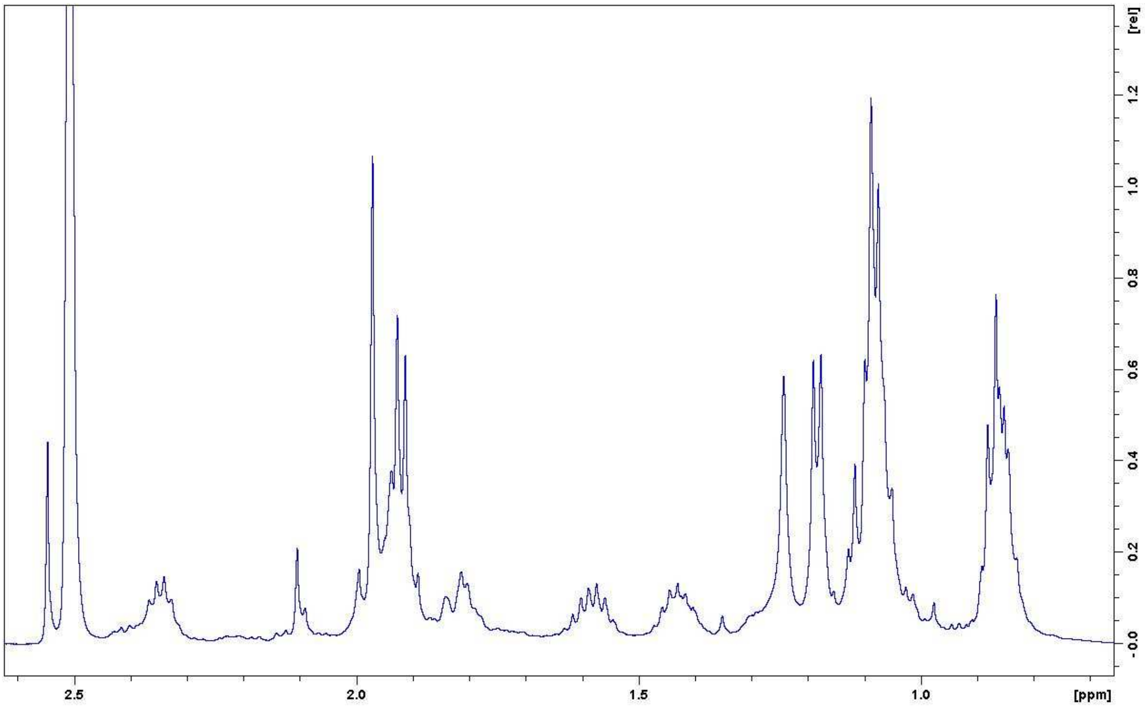

Figure S80. ^{1}H-^{13}C **HSQC spectrum of 3'-*O*-demethyl-paulomycin A (19) (DMSO-*d*$_6$, 500 MHz, 24 °C).**

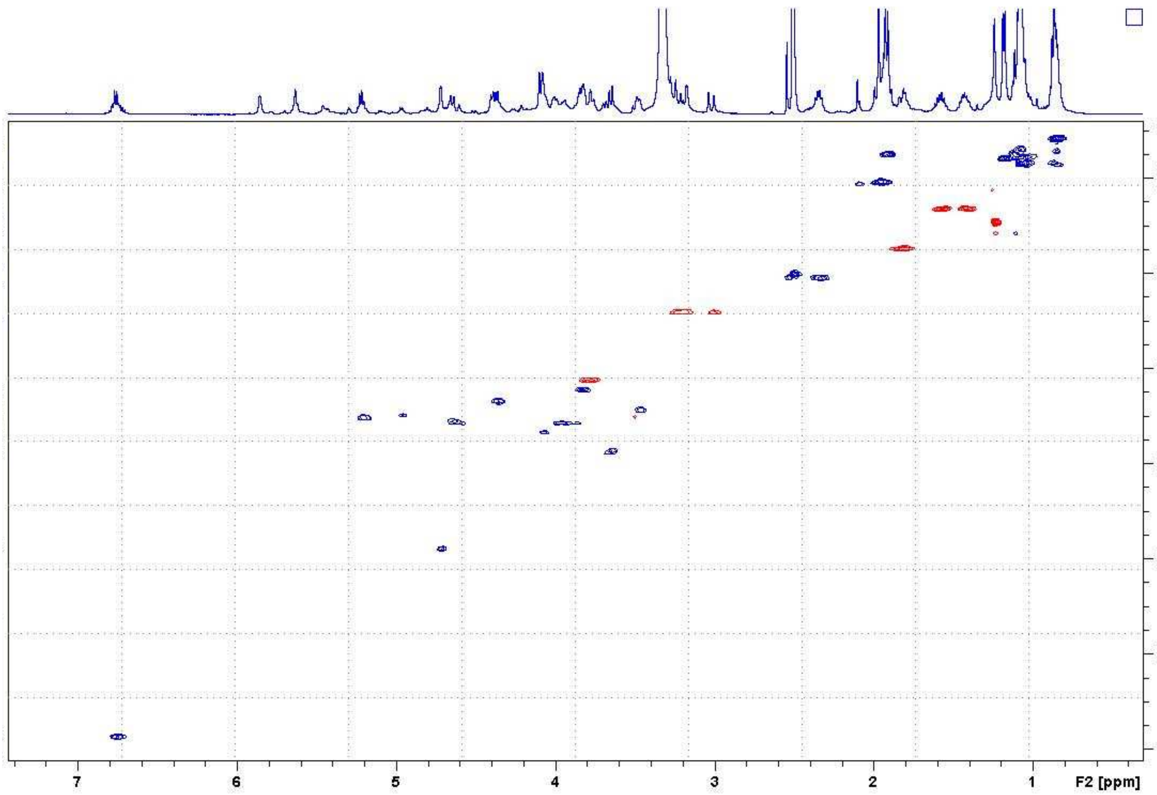

Figure S81. Structure of 3'-*O*-demethyl-paulomycin A (19).

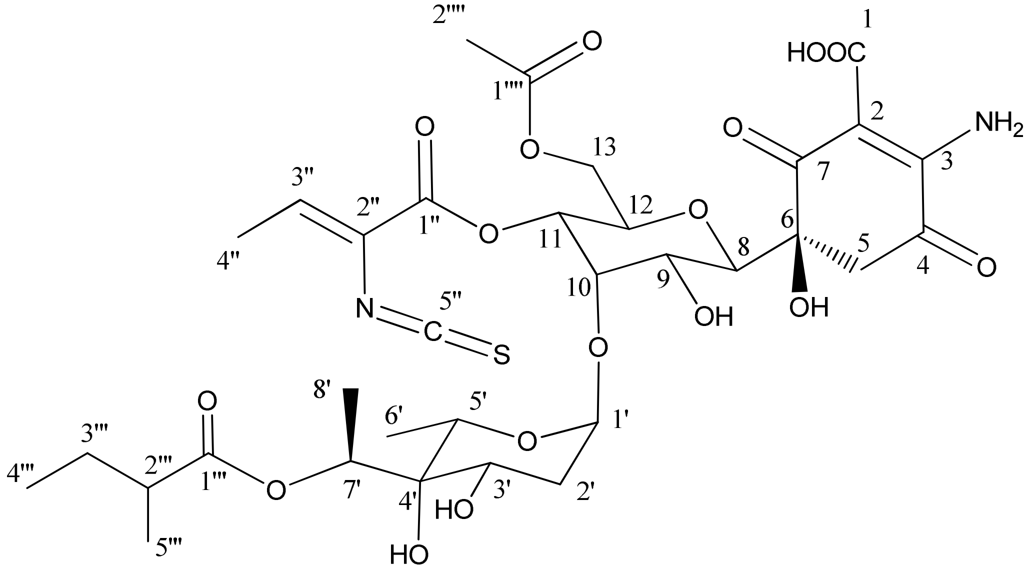

Table S10. 3'-*O*-demethyl-paulomycin A (19) ^{13}C and ^{1}H NMR data (δ in ppm) acquired in DMSO-*d6* (500 MHz, 24 °C).

Pos.	δ (^{13}C)	δ (^{1}H), (mult, *J* in Hz)	Pos.	δ (^{13}C)	δ(^{1}H), (mult, *J* in Hz)
1	n.d.	-	1''	160.1	-
2	n. d.	-	2''	122.7	-
3	159.5	-	3''	137.4	6.76 (quart., 7.1)
4	188.8	-	4''	15.1	1.92 (d, 7.1)
5	48.2	3.23 (m)<br>3.02 (d, 16.2)	5''	141.8	-
6	77.9	-	1'''	175.2	-
7	198.1	-	2'''	41.0	2.34 (sext., 6.8)
8	77.3	3.65 (d, 9.8)	3'''	26.5	1.58 (m)<br>1.43 (m)
9	68.7	3.48 (br d, 9.3)	4'''	11.8	0.86 (t, 6.9)
10	73.5	4.08 (m)	5'''	17.0	1.07 (d, 6.5)
11	71.1	4.65 (br d, 9.9)	1''''	170.4	-
12	71.5	4.01 (m)	2''''	20.8	1.97 (s)
13	62.4	3.82 (m)<br>3.78 (m)			
1'	97.9	4.72 (br s)			
2'	34.8	1.82 (m)			
3'	64.5	3.84 (dd, 11.1, 4.4)			
4'	73.7	-			
5'	66.9	4.37 (m)			
6'	15.8	1.08 (d, 6.5)			
7'	70.4	5.21 (quart., 6.7)			
8'	15.9	1.18 (d, 6.6)			

δ^{13}C were determined from HSQC and HMBC spectra.

Structural characterization of 3'-demethoxyl-paulomycin A (20)

The molecular formula of compound **20**, $C_{33}H_{44}N_2O_{16}S$, was established based on the pseudomolecular ion peak $[M\text{+H}]^+$ observed at 757.2485 (Figure S82). The base peak in the mass spectrum does not correspond to the pseudomolecular ion but to a fragment at *m/z* = 515.0964. Such a behavior is typical for paulomycins (at least in our equipment and under the ionization conditions employed) and it was already found for paulomycin F (**12**). This observed in-source fragmentation is due to the easy breakage of the glycosidic bond of paulomycins. The mentioned "515" fragment displays the same molecular structure in the paulomycin series. The ^{1}H and the HSQC NMR spectra (Figures S83-S87) confirmed the paulomycin-like connectivity and, as expected, many

of the observed signals show a strong resemblance to those displayed by paulomycin F (**12**). The presence of the paulic acid moiety was confirmed.

The establishment of the structure required the acquisition of additional 2D NMR spectra including, COSY, TOCSY, NOESY and HMBC. After detailed analysis of such spectral set, the structure of the main compound was determined. The structure is identical to paulomycin A but lacking the methoxyl group at position 3' (Figure S88) which now is a methylene carbon resonating as multiplets at 1.55 and 1.93 in the ^{1}H NMR spectrum.

Figure S82. (+) ESI-TOF spectrum of 3'-demethoxyl-paulomycin A (20).

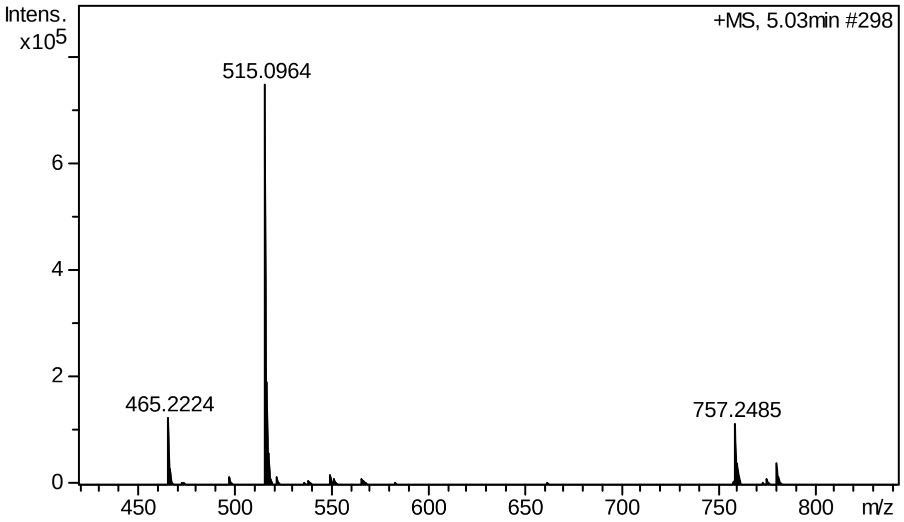

Figure S83. ^{1}H NMR spectrum of 3'-demethoxyl-paulomycin A (20) (DMSO-d_6, 500 MHz, 24 °C).

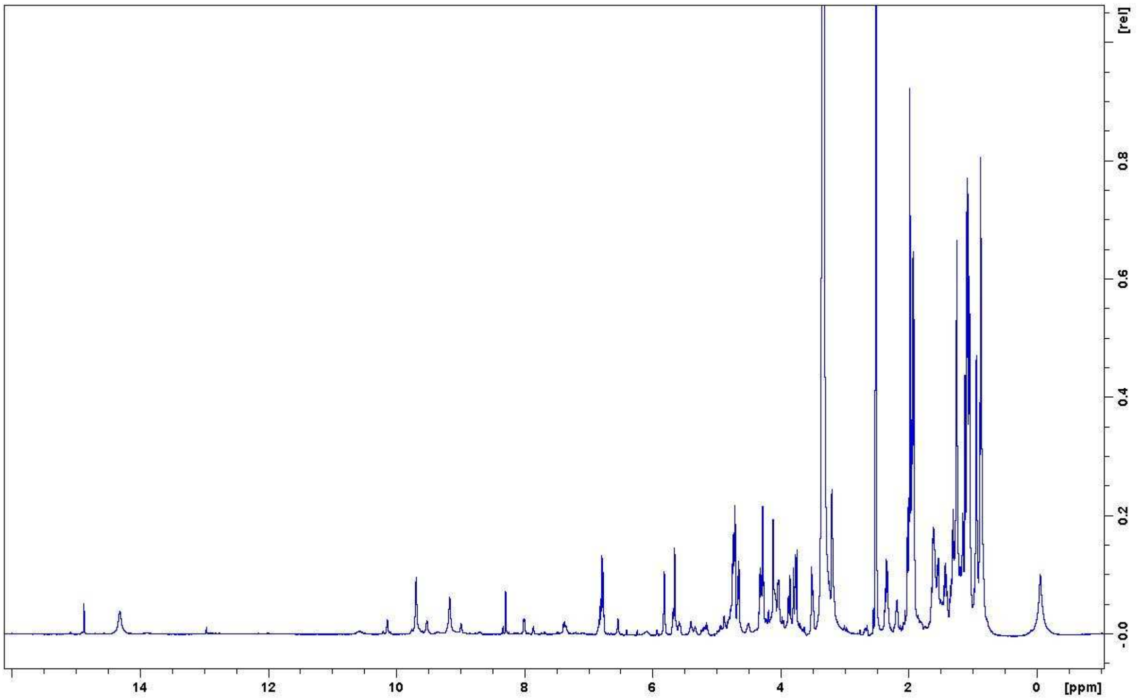

Figure S84. Expansion of ^{1}H NMR spectrum of 3'-demethoxyl-paulomycin A (20) (DMSO-d_6, 500 MHz, 24 °C).

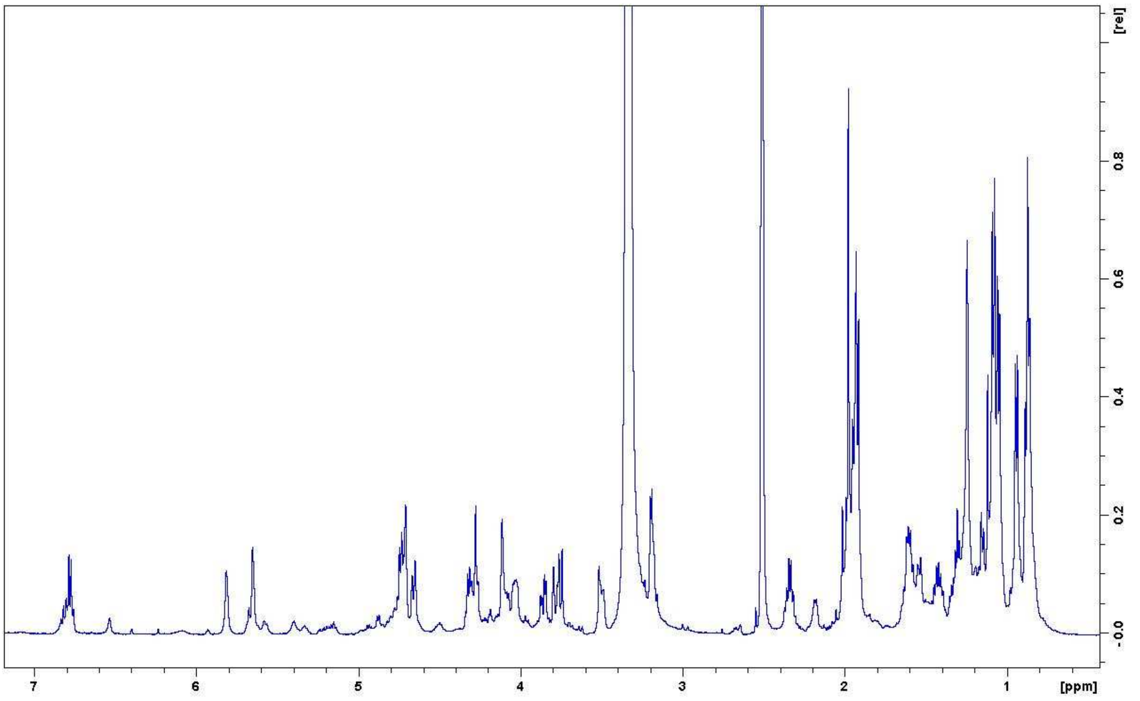

Figure S85. Expansion of ^{1}H NMR spectrum of 3'-demethoxyl-paulomycin A (20) (DMSO-d_6, 500 MHz, 24 °C).

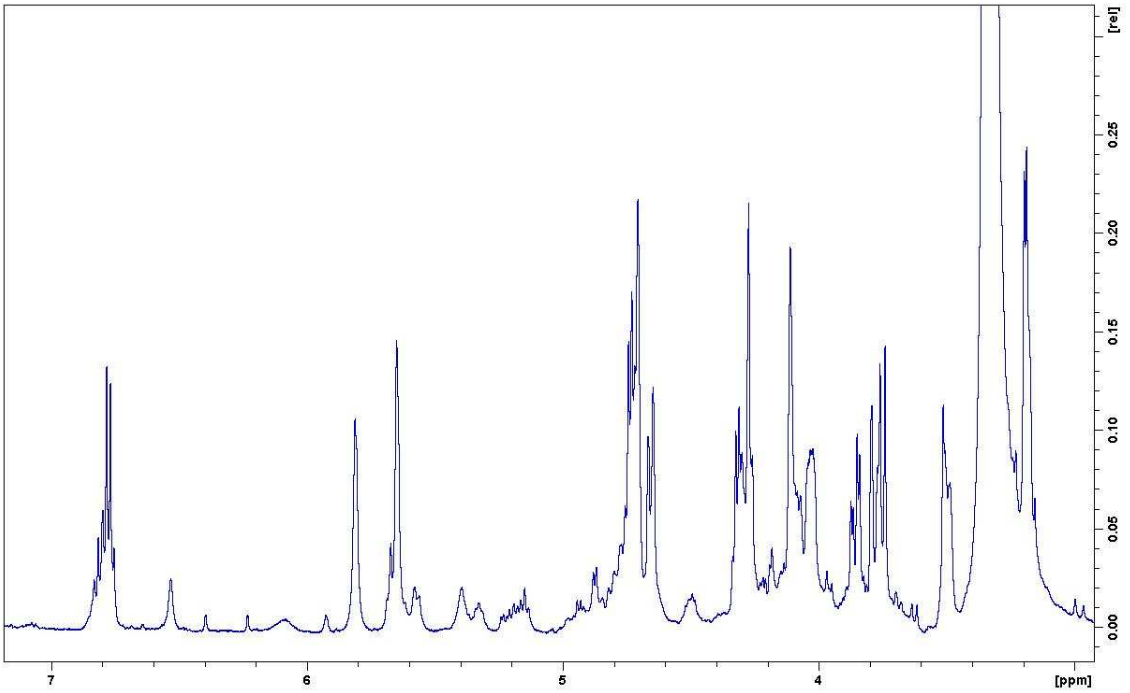

Figure S86. Expansion of ^{1}H NMR spectrum of 3'-demethoxyl-paulomycin A (20) (DMSO-d_6, 500 MHz, 24 °C).

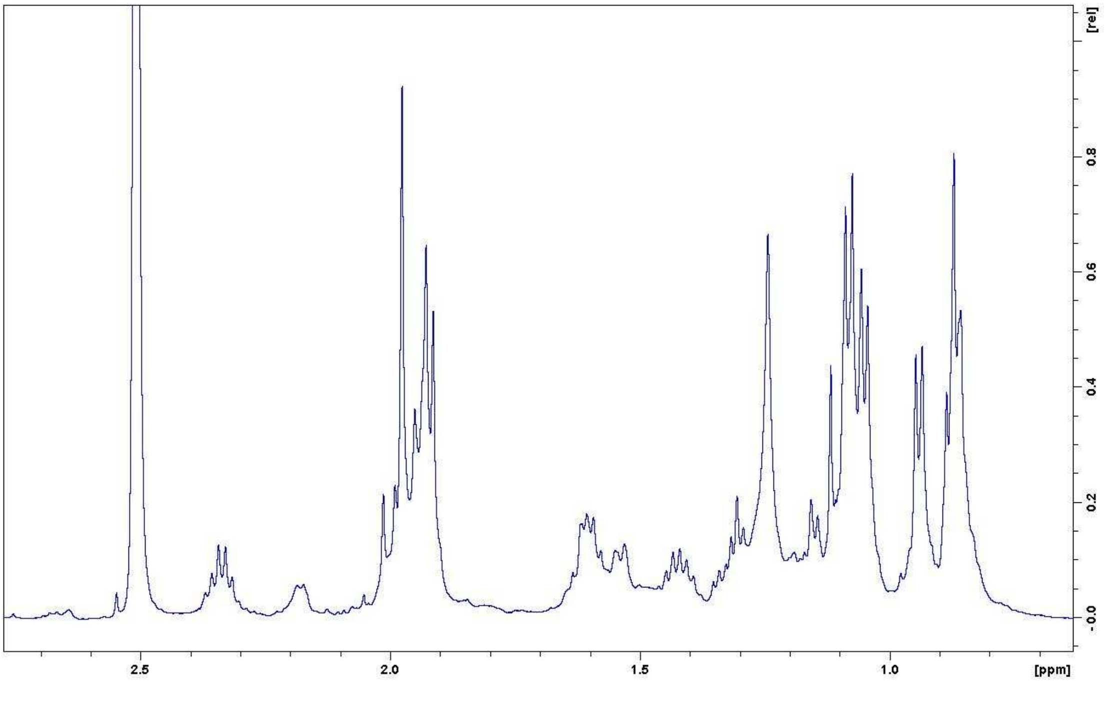

Figure S87. ^{1}H-^{13}C HSQC spectrum of 3'-demethoxyl-paulomycin A (20) (DMSO-d_6, 500 MHz, 24 °C).

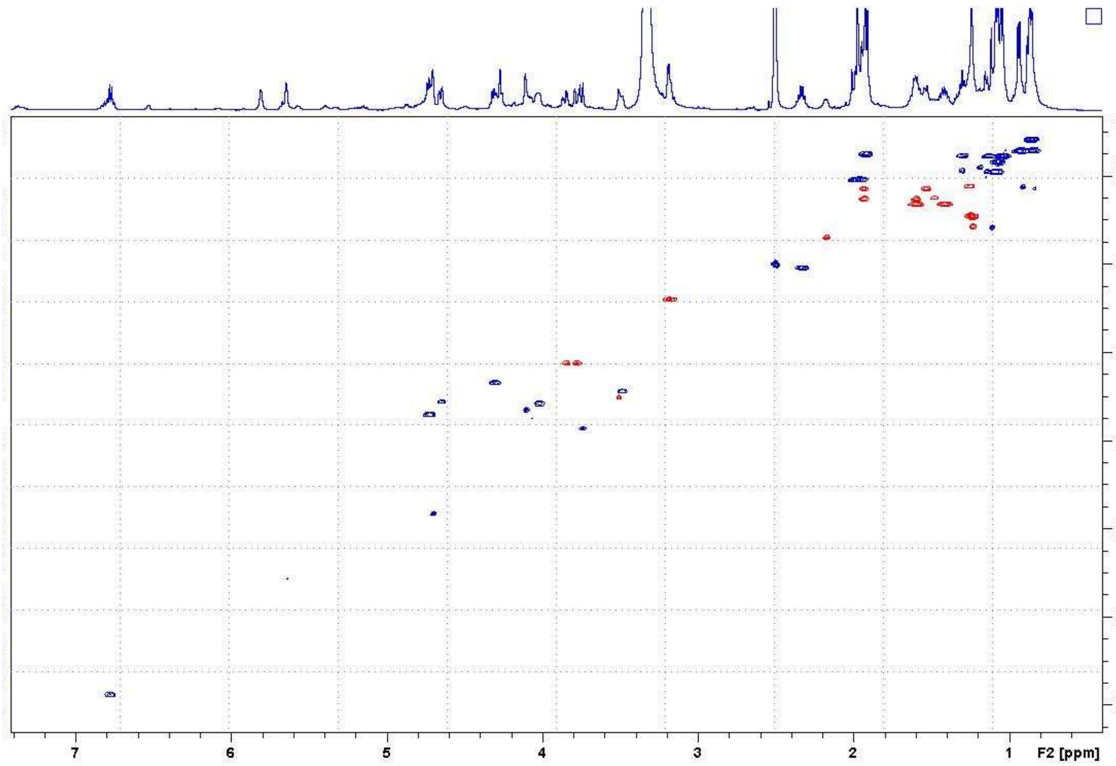

Figure S88. Structure of 3'-demethoxyl-paulomycin A (20).

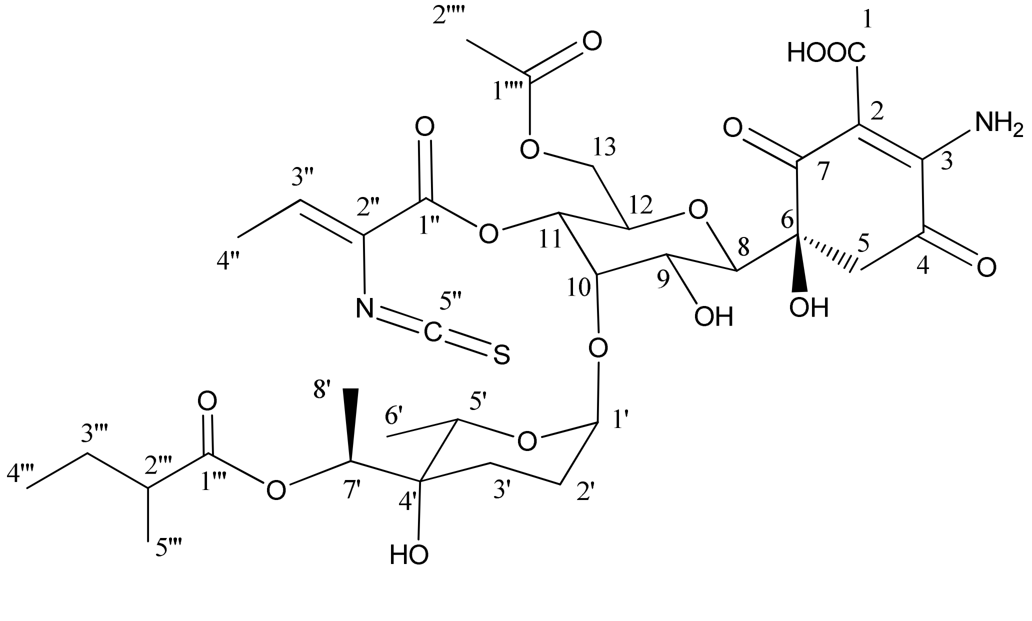

Table S11. 3’-demethoxyl-paulomycin A (20) ^{13}C and ^{1}H NMR data (δ in ppm) acquired in DMSO-*d6* (500 MHz, 24 °C).

Pos.	δ (^{13}C)	δ (^{1}H), (mult, *J* in Hz)	Pos.	δ (^{13}C)	δ(^{1}H), (mult, *J* in Hz)
1	n.d.	-	1’’	160.2	-
2	n. d.	-	2’’	122.7	-
3	n. d.	-	3’’	137.7	6.78 (quart., 7.2)
4	189.0	-	4’’	15.1	1.92 (d, 7.1)
5	48.1	3.19 (m)	5’’	141.7	-
6	77.9	-	1’’’	175.4	-
7	198.3	-	2’’’	41.0	2.34 (sext., 6.8)
8	77.3	3.76 (d, 10.0)	3’’’	26.4	1.60 (m)<br>1.41 (m)
9	68.8	3.50 (br d, 9.2)	4’’’	11.7	0.87 (t, 6.8)
10	73.0	4.11 (m)	5’’’	16.8	1.07 (d, 6.7)
11	71.3	4.66 (br d, 10.0)	1’’’’	170.4	-
12	71.6	4.03 (m)	2’’’’	20.8	1.98 (s)
13	62.5	3.85 (dd, 11.7, 4.6)<br>3.77 (br d, 11.9)			
1’	96.7	4.71 (br s)			
2’	25.2	1.94 (m)<br>1.60(m)			
3’	23.0	1.93 (m)<br>1.55 (m)			
4’	71.2	-			
5’	66.9	4.31 (quart., 6.4)			
6’	14.3	0.94 (d, 6.3)			
7’	74.1	4.73 (quart., 6.5)			
8’	15.5	1.05 (d, 6.5)			

δ^{13}C were determined from HSQC and HMBC spectra.

Structural characterization of 3’-demethoxyl-paulomycin B (21).

The molecular formula of compound **21**, $C_{32}H_{42}N_2O_{16}S$, was established based on the pseudomolecular ion peak $[M+H]^+$ observed at 743.2336 (Figure S89). As expected, the base peak in the mass spectrum does not correspond to the pseudomolecular ion but to a fragment at *m/z* = 515.0969. The presence of such diagnostic fragment further confirmed the paulomycin nature of the molecule (the “515” fragment display the same molecular structure in the paulomycin series). The ^{1}H and the HSQC NMR (Figure S90-S4) spectra were almost identical to those of compound **20** and the differences were

only found in the aliphatic signals from the acid that esterifies the carbohydrate at position 7'.

The establishment of the structure (essentially the nature of the fatty acid esteryfying position 7') required the acquisition of additional 2D NMR spectra including, COSY, TOCSY, NOESY and HMBC. After analysis of such spectral set, the structure of the main compound was determined. The structure is identical to compound **20** but lacking the methyl group at position 4''' to render a isobutyric acid moiety. The structure is thus identical to paulomycin B but lacking the methoxyl group at position 3' which now is a methylene carbon (Figure S95).

Figure S89. (+) ESI-TOF spectrum of 3'-demethoxyl-paulomycin B (21).

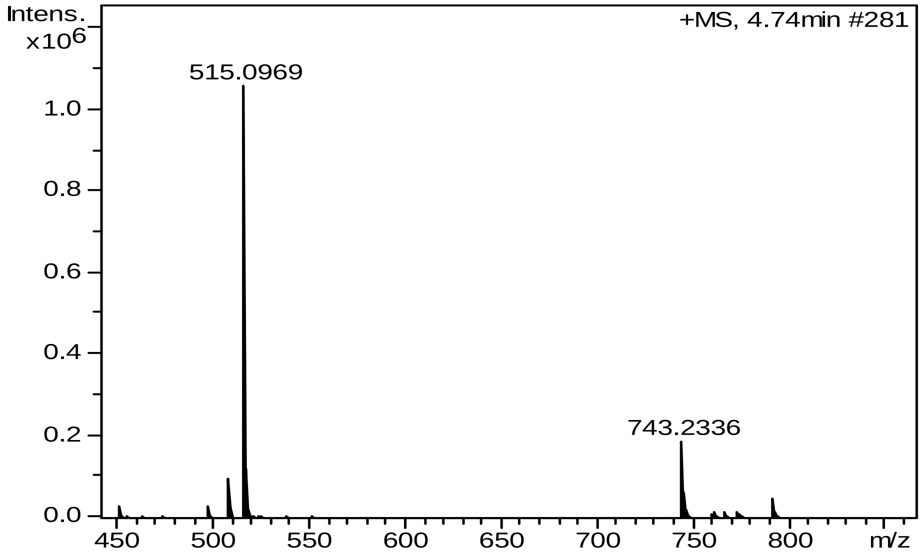

Figure S90. ^{1}H NMR spectrum of 3'-demethoxyl-paulomycin B (21) (DMSO-d_6, 500 MHz, 24 °C).

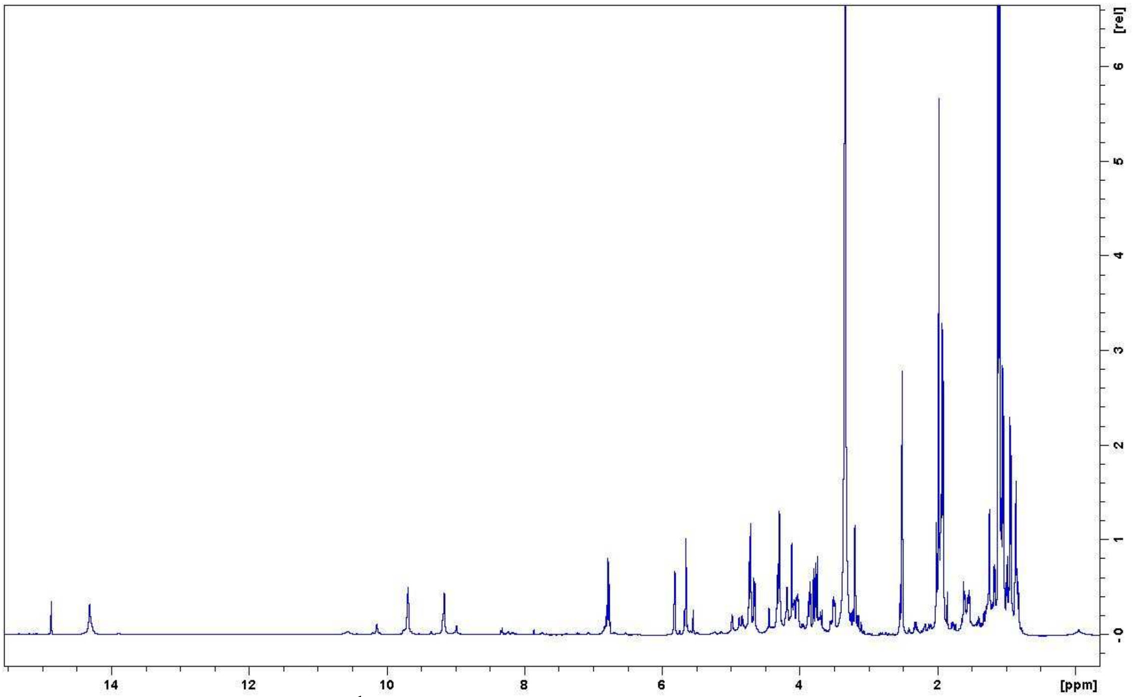

Figure S91. Expansion of ^{1}H NMR spectrum of 3'-demethoxyl-paulomycin B (21) (DMSO-d_6, 500 MHz, 24 °C).

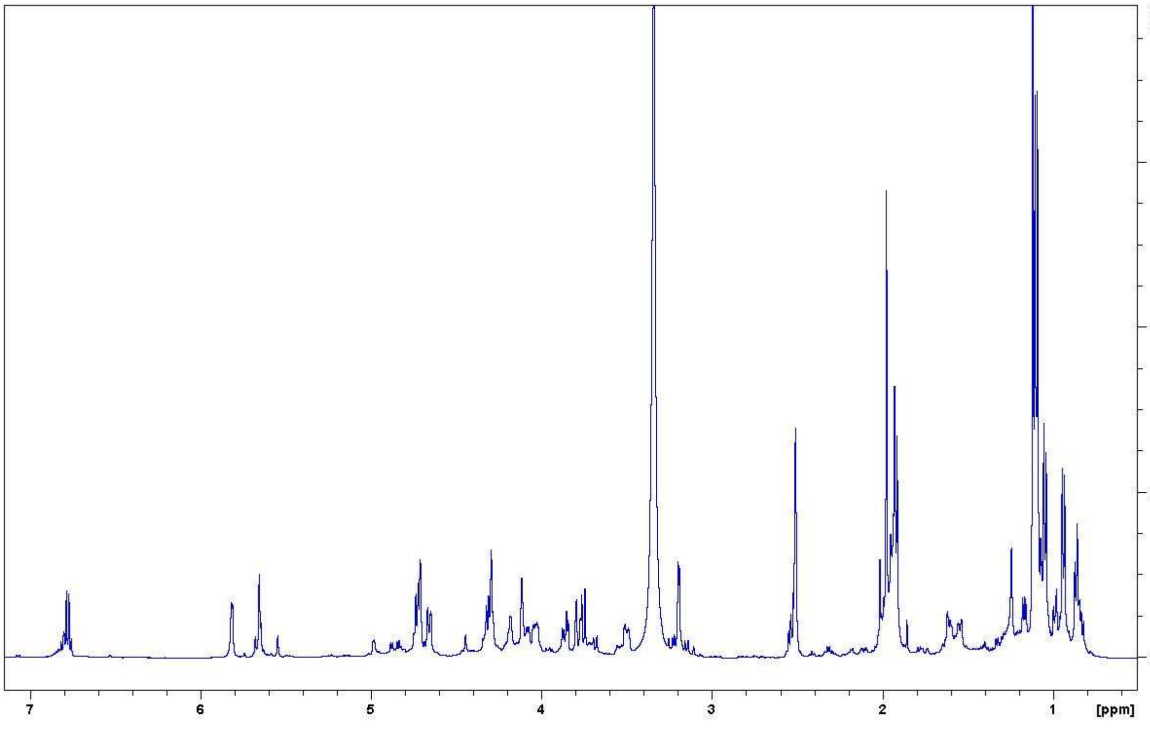

Figure S92. Expansion of ^{1}H NMR spectrum of 3'-demethoxyl-paulomycin B (21) (DMSO-d_6, 500 MHz, 24 °C).

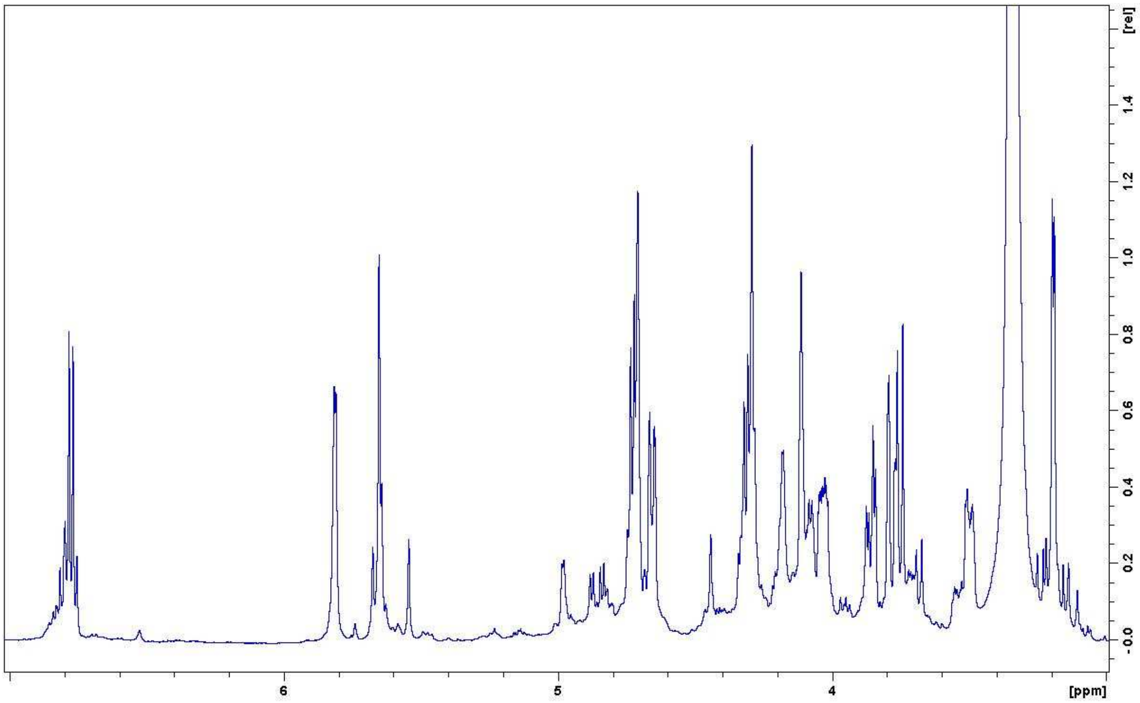

Figure S93. Expansion of ^{1}H NMR spectrum of 3'-demethoxyl-paulomycin B (21) (DMSO-d_6, 500 MHz, 24 °C).

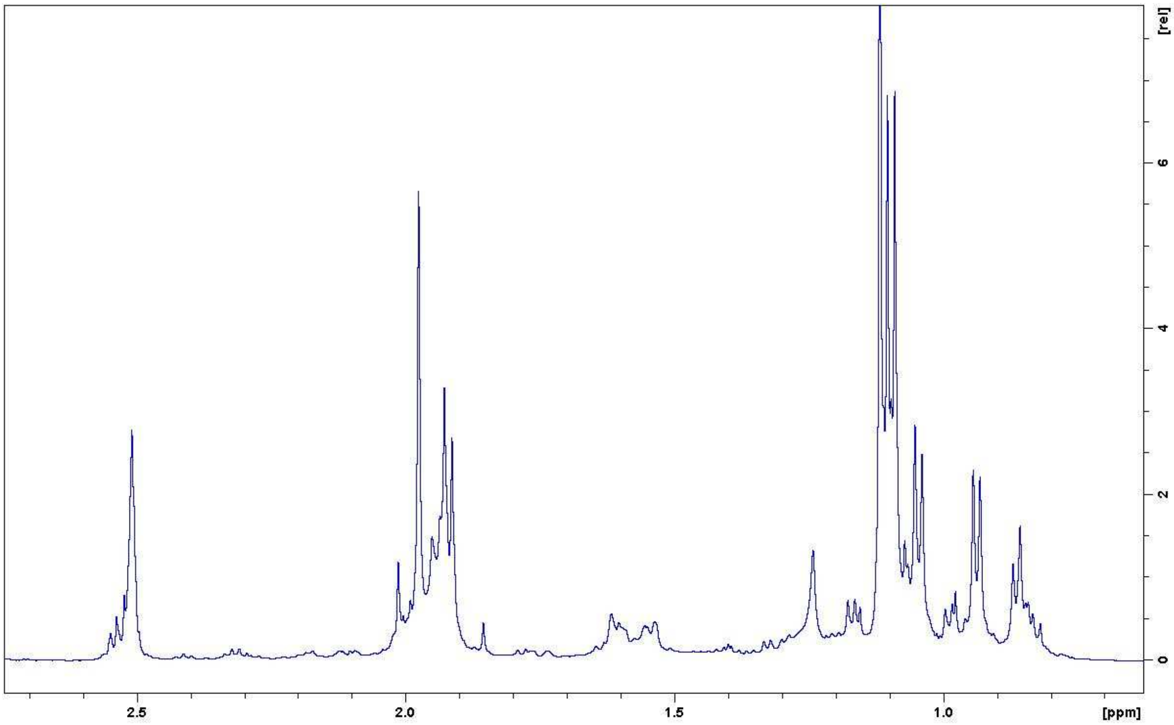

Figure S94. ^{1}H-^{13}C **HSQC spectrum of 3'-demethoxyl-paulomycin B (21) (DMSO-*d*$_6$, 500 MHz, 24 °C).**

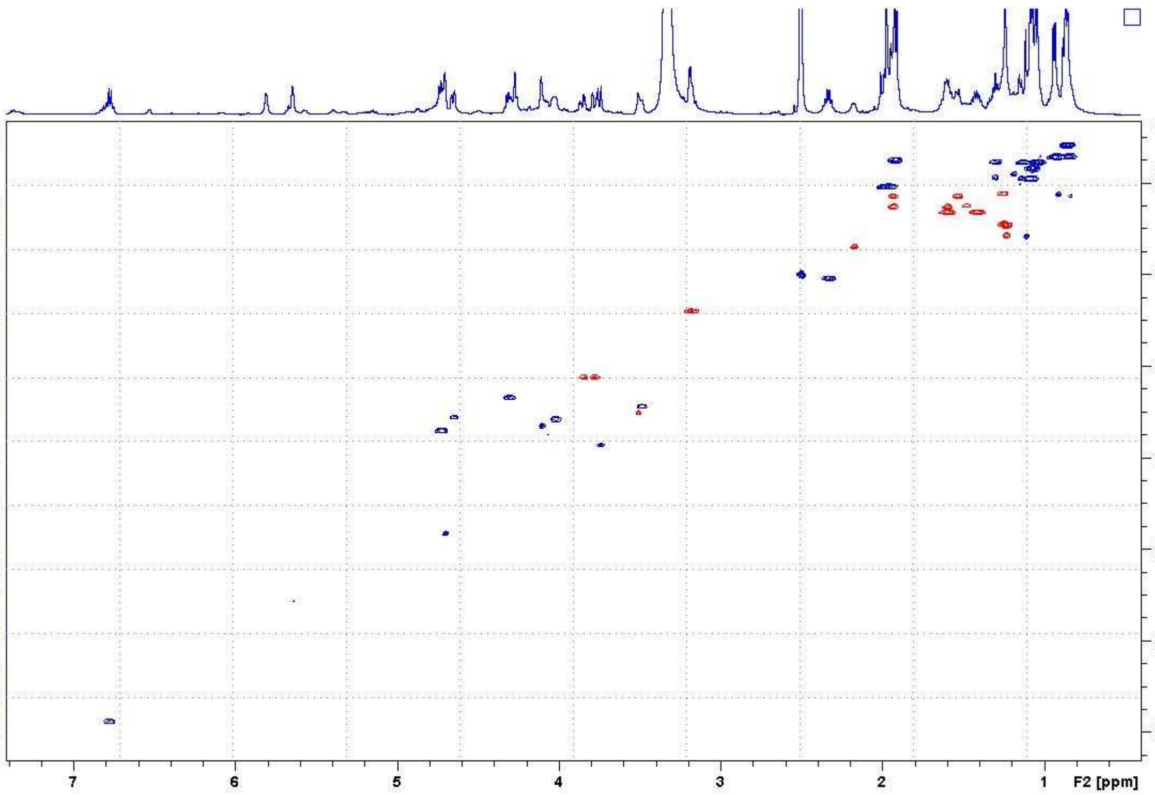

Figure S95. Structure of 3'-demethoxyl-paulomycin B (21).

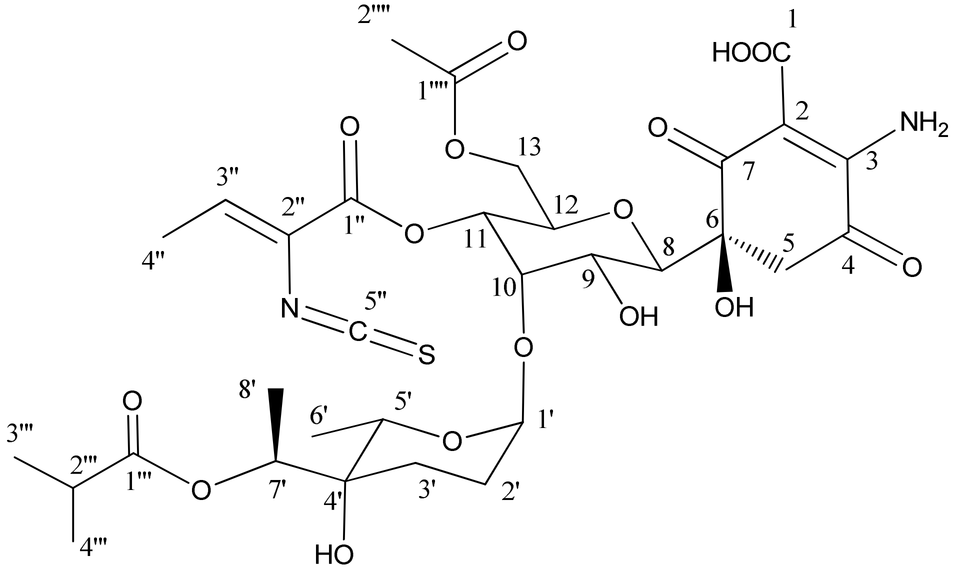

Table S12. 3'-demethoxyl-paulomycin B (21) ^{13}C and ^{1}H NMR data (δ in ppm) acquired in DMSO-*d6* (500 MHz, 24 °C).

Pos.	δ (^{13}C)	δ (^{1}H), (mult, *J* in Hz)	Pos.	δ (^{13}C)	δ(^{1}H), (mult, *J* in Hz)
1	n.d.	-	1''	160.2	-
2	n. d.	-	2''	122.7	-
3	n. d.	-	3''	137.7	6.78 (quart., 7.2)
4	189.0	-	4''	15.1	1.92 (d, 7.1)
5	48.1	3.19 (m)	5''	141.7	-
6	77.9	-	1'''	175.9	-
7	198.3	-	2'''	33.8	2.52 (sext., 6.8)
8	77.3	3.76 (d, 10.0)	3'''	19.5	1.09 (t, 6.9)
9	68.8	3.50 (br d, 9.2)	4'''	19.5	1.09 (t, 6.9)
10	73.0	4.11 (m)	1''''	170.4	-
11	71.3	4.66 (br d, 10.0)	2''''	20.8	1.98 (s)
12	71.6	4.03 (m)			
13	62.5	3.85 (dd, 11.7, 4.6)<br>3.77 (br d, 11.9)			
1'	96.7	4.71 (br s)			
2'	25.2	1.94 (m)<br>1.60(m)			
3'	23.0	1.93 (m)<br>1.55 (m)			
4'	71.2	-			
5'	66.9	4.31 (quart., 6.4)			
6'	14.3	0.94 (d, 6.3)			
7'	74.1	4.73 (quart., 6.5)			
8'	15.5	1.05 (d, 6.5)			

δ^{13}C were determined from HSQC and HMBC spectra.

References

1. Argoudelis AD, Brinkley TA, Brodasky TF, Buege JA, Meyer HF, Mizsak SA. Paulomycins A and B. Isolation and characterization. J Antibiot. 1982;35:285-294.
2. Argoudelis AD, Baczynskyj L, Mizsak SA, Shilliday FB. O-demethylpaulomycins A and B, U-77,802 and U-77,803, paulomenols A and B, new metabolites produced by *Streptomyces paulus*. J Antibiot. 1988;41:1316-1330.
3. Argoudelis AD, Baczynskyj L, Mizsak SA, Shilliday FB, Wiley PF. Structural relationships between senfolomycins and paulomycins. J Antibiot. 1988;41:1212-1222.
4. Guan SH, Sattler I, Lin WH, Guo DA, Grabley S. *p*-Aminoacetophenonic acids produced by a mangrove endophyte: *Streptomyces griseus* subsp. J Nat Prod. 2005;68:1198-1200.
5. Wang F, Xu M, Li Q, Sattler I, Lin W. *p*-Aminoacetophenonic acids produced by a mangrove endophyte *Streptomyces* sp. (strain HK10552). Molecules. 2010;15:2782-2790.
6. Buckingham J. Dictionary of Natural Products on DVD, version 22.2. Taylor & Francis/CRC Press, London; 2013.
7. Argoudelis AD, Baczynskyj L, Haak WJ, Knoll WM, Mizsak SA, Shilliday FB. New paulomycins produced by *Streptomyces paulus*. J Antibiot. 1988;41:157-169.
8. Wiley PF, Mizsak SA, Baczynskyj L, Argoudelis AD, Duchamp DJ, Watt W. The structure and chemistry of paulomycin. J Org Chem. 1986;51:2493-2499.
