# Supplementary material for: New insights into paulomycin biosynthesis pathway in Streptomyces albus J1074 and generation of novel derivatives by combinatorial biosynthesis
Source: Microb Cell Fact. 2016 Mar 21;15:56. doi: 10.1186/s12934-016-0452-4 (PMC4802897; doi:10.1186/s12934-016-0452-4)

Bioactivity testing of paulomycins

The antibacterial activity of paulomycin A (**1**), paulomycin B (**2**), paulomycin E (**3**), paulomycin F (**12**), 13-*O*-deacetyl-13-*O*-paulyl-paulomycin E (**14'**), 13-*O*-deacetyl-13-*O*-paulyl-paulomycin B (**15'**), 13-*O*-deacetyl-13-*O*-paulyl-paulomycin A (**16'**), 3'-*O*-demethyl-paulomycin B (**18**), 3'-*O*-demethyl-paulomycin A (**19**), 3'-demethoxyl-paulomycin A (**20**) and 3'-demethoxyl-paulomycin B (**21**) was analyzed via antibiotic disc diffusion assay against *Escherichia coli*, *Pseudomonas aeruginosa*, *Serratia marcenscens*, *Klebsiella pneumoniae*, *Micrococcus luteus*, *Staphylococcus aureus*, *Staphylococcus epidermidis*, and *Streptococcus agalactiae*. To do this, paper disks of 5 mm in diameter were used. Each disk contained a solution of 2 μg of each compound dissolved in 15 μl *ter*-butanol/water (1:1). Fifteen microliters of *ter*-butanol/water were used as negative control (Figure S96). The antibiotics were allowed to diffuse from the disk at 4°C during 2 hr and then the plates were incubated at 30°C for 24 hr.

Figure S96. Antibacterial activity test of paulomycins and their derivatives. Antibacterial activity of paulomycin A (**1**), paulomycin B (**2**), paulomycin E (**3**), paulomycin F (**12**), 13-*O*-deacetyl-13-*O*-paulyl-paulomycin E (**14’**), 13-*O*-deacetyl-13-*O*-paulyl-paulomycin B (**15’**), 13-*O*-deacetyl-13-*O*-paulyl-paulomycin A (**16’**), 3'-*O*-demethyl-paulomycin B (**18**), 3'-*O*-demethyl-paulomycin A (**19**), 3'-demethoxy-paulomycin A (**20**) and 3'-demethoxy-paulomycin B (**21**), assessed against: A) *Micrococcus luteus*, B) *Staphylococcus aureus*, C) *Staphylococcus epidermidis* and D) *Streptococcus agalactiae*. 2 µg of each compound dissolved in 15 µl of *ter*-butanol/water were used. The diameter (mm) of the inhibition-growth halo is indicated in parenthesis. 15 µl of *ter*-butanol/water were used as negative control.

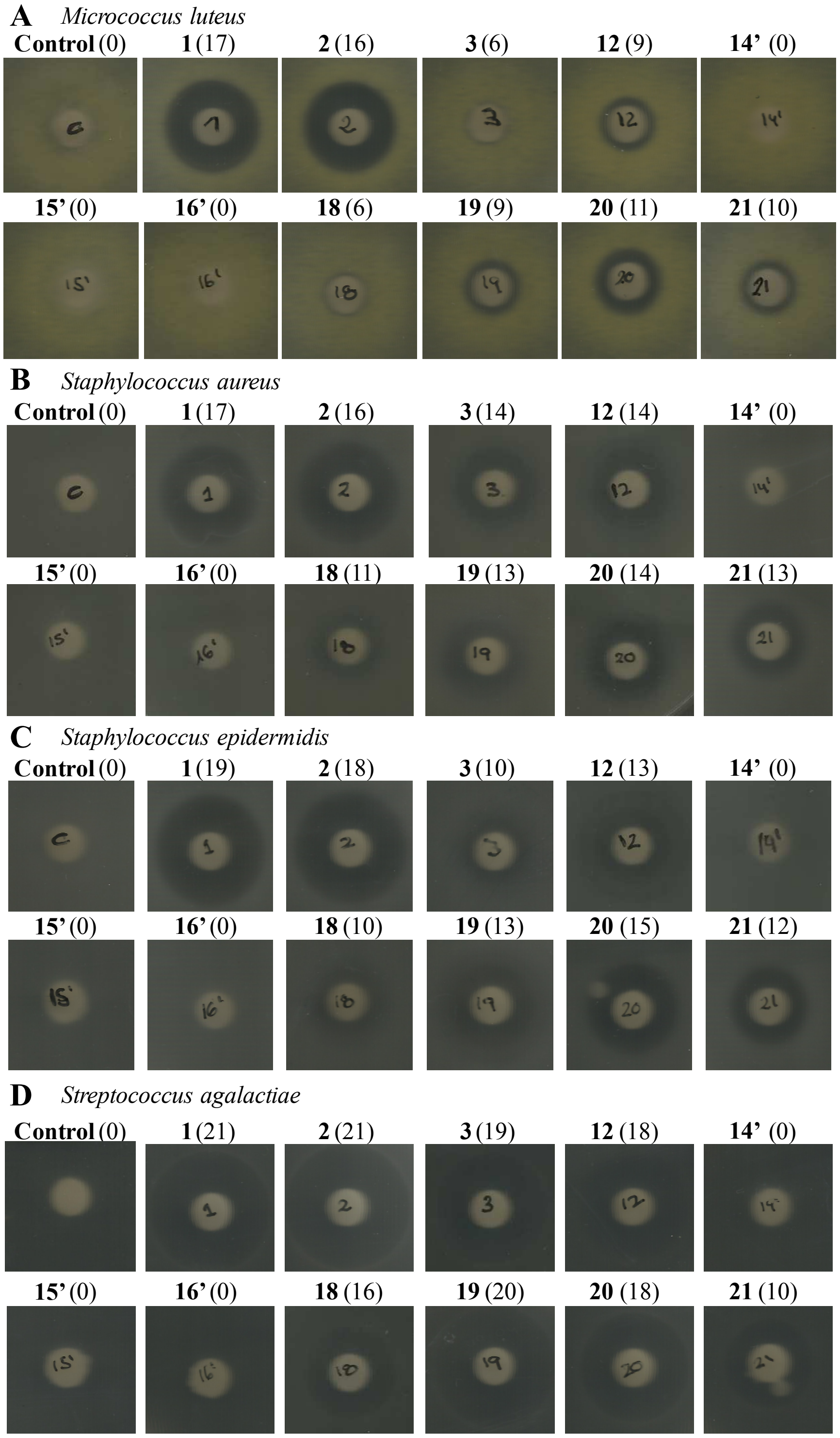

Supplement: Supplementary file 4 — 10.1186/s12934-016-0452-4 Methods. Bioactivity testing of paulomycins. Figure S96. Antibacterial activity test of paulomycins and their derivatives. Format: PDF. [file 12934_2016_452_MOESM4_ESM.pdf]
